# Boosting the Reactivity of Bis-Lactones to Enable Step-Growth Polymerization at Room Temperature

**DOI:** 10.1021/acs.macromol.3c02527

**Published:** 2024-03-22

**Authors:** Marta Ximenis, Julien Monot, Elena Gabirondo, Janna Jeschke, Blanca Martín-Vaca, Didier Bourissou, Haritz Sardon

**Affiliations:** † POLYMAT, University of the Basque Country UPV/EHU, Joxe Mari Korta Center Avda. Tolosa 72, 20018 Donostia-San Sebastian, Spain; ‡ Laboratoire Hétérochimie Fondamentale et Appliquée (UMR 5069), Université de Toulouse (UPS), CNRS, 118 Route de Narbonne, F-31062 Toulouse, France; § POLYMAT and Department of Polymers and Advanced Materials/Physics, Chemistry and Technology, University of the Basque Country UPV/EHU, Joxe Mari Korta Center Avda. Tolosa 72, 20018 Donostia-San Sebastian, Spain

## Abstract

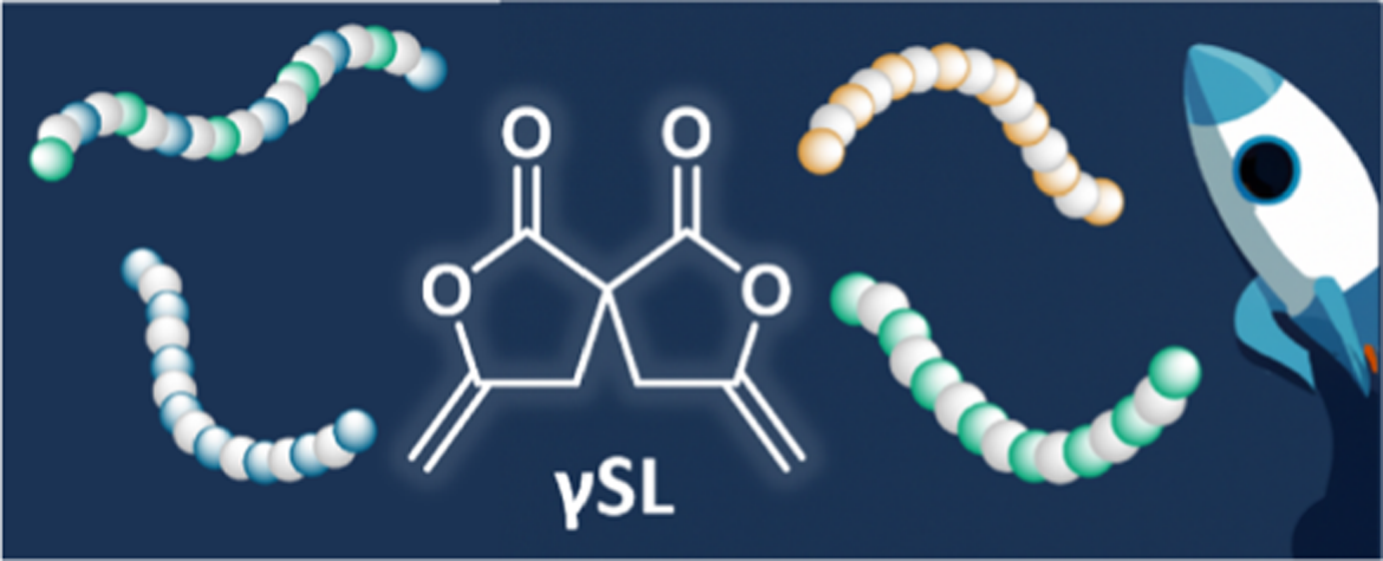

The development of new sustainable polymeric materials endowed with improved performances but minimal environmental impact is a major concern, with polyesters as primary targets. Lactones are key monomers thanks to ring-opening polymerization, but their use in step-growth polymerization has remained scarce and challenging. Herein, we report a powerful bis(γ-lactone) (**γSL**) that was efficiently prepared on a gram scale from malonic acid by Pd-catalyzed cycloisomerization. The γ-exomethylene moieties and the spiro structure greatly enhance its reactivity toward ring-opening and enable step-growth polymerization under mild conditions. Using diols, dithiols, or diamines as comonomers, a variety of regioregular (AB)_*n*_ copolymers with diverse linkages and functional groups (from oxo-ester to β-thioether lactone and β-hydroxy-lactame) have been readily prepared. Reaction modeling and monitoring revealed the occurrence of an original *trans*-lactonization process following the first ring-opening of **γSL**. This peculiar reactivity opens the way to regioregular (ABAC)_*n*_ terpolymers, as illustrated by the successive step-growth polymerization of **γSL** with a diol and a diamine.

## Introduction

Plastics have become pivotal to human life, with a world production as high as 390.7 Mt in 2021.^1^ Even though the use of plastics keeps increasing in a broad range of applications, bringing social, technological, and economic benefits, their extensive use and poor end-of-life management induce waste of resources and environmental damage. In recent years, there has been a rising concern to mitigate this impact.^2,3^ Several strategies are nurtured, such as the use of biorenewable sources as counterparts of fossil-fuel-based feedstocks,^4,5^ the recycling/upcycling of commodity polymers,^6−8^ and the design of plastics made to be recycled.^9−12^

In this regard, polyesters are most attractive and occupy a forefront position. Their C(=O)–O linkages are relatively easy to form and cleave, making polymerization and degradation/depolymerization not very demanding energetically.^6,13−15^ As for the preparation of polyesters, two routes are widely used: polycondensation and ring-opening polymerization (ROP). Polycondensation of diacids or diesters with diols (or hydroxy acids) is the most attractive industrially as it uses readily available monomers, is more robust, and does not require inert conditions.^16−18^ However, it often requires harsh polymerization conditions and high vacuum to remove condensate coproducts and achieve high molecular weights. On the other hand, the ROP of lactones usually takes place under milder conditions^19−21^ and enables better control of the polymer structure, molar masses, and chain ends. The efficiency of the ROP strongly depends on the ring size and ring strain of the lactone, with δ- and ε-lactones (6- and 7-membered rings) being the most reactive and used ones.^22−24^ One of the drawbacks of the ROP of lactones is the limited variety of monomers and the synthetic efforts required to develop new ones, in particular when the monomer synthesis relies on cyclization, which is inherently in competition with intermolecular reactions. In this regard, γ-lactones (5-membered rings) are the easiest to prepare, but the ROP of these nonstrained lactones is challenging, and despite some recent noticeable achievements,^25,26^ it remains far from general and difficult to apply on a large scale.^27,28^

A possible alternative to the polycondensation and ROP routes is the step-growth polymerization of bifunctional cyclic monomers with diols and, more generally, bis-nucleophiles. This is a very attractive approach as it may combine key advantages, in particular, easy and broad structural diversity (by varying the bis-nucleophile partner) and mild conditions/full atom economy (by involving kinetically accessible and thermodynamically favorable ring-opening). However, this approach has only been very scarcely investigated, and the results obtained so far point out severe limitations.

**Scheme 1:**
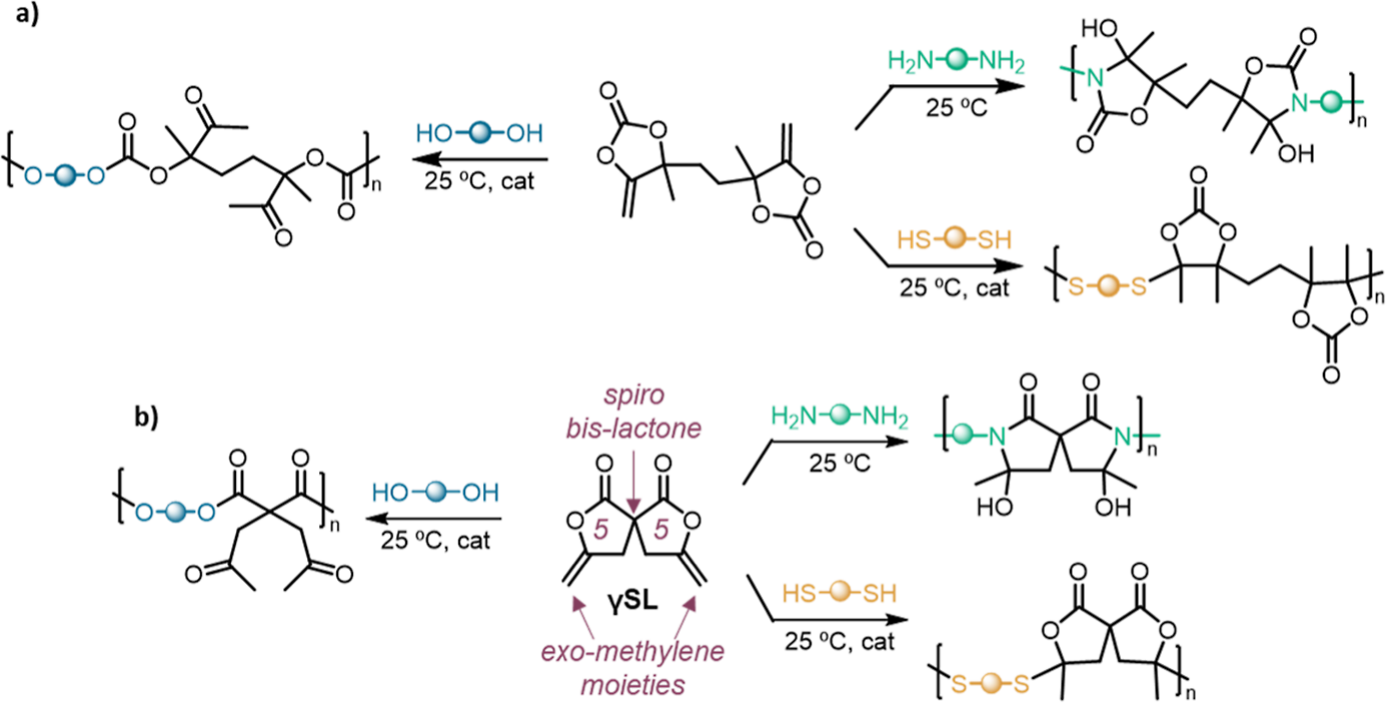
(a) Model Bis(α-exovinylene cyclic carbonate) Developed by Detrembleur et al. and Derived Polymers; (b) Spiro Bis(γ-exomethylene γ-lactone) **γSL** Designed and Targeted in This Work as a Way to Achieve Step-Growth Polymerization under Mild Conditions and Access Polyesters, Poly(spiro bis(β-thioether lactones)), and Poly(spiro bis(β-hydroxy-lactames))

Indeed, when bis-lactones were used, the alcohol functionality released upon ring-opening reacted further and led to side-processes. As a result, bis-lactones have rather been applied as reticulating agents in the ROP of lactones.^29−32^ Another possibility is to employ bis-anhydrides, which are easily ring-opened by diols at room temperature. However, carboxylic acids are generated, and only low molecular weights have been achieved using this route, even at long reaction times.^33^ In fact, the bottleneck for such step-growth polymerization is to identify suitable bifunctional cyclic monomers with appropriate reactivity. To this end, we were inspired by the recent work of Detrembleur et al. on five-membered cyclic carbonates. They showed that the presence of an exocyclic C=C double bond significantly increases the reactivity toward ring-opening and could achieve step-growth polymerization at room temperature (Scheme 1a).^34−36^ On this basis, we designed the spiro bis-lactone monomer **γSL** (Scheme 1b).^37^ The γ-exomethylene moiety was surmised to increase the reactivity of the lactone toward ring-opening (kinetically) due to the better leaving group character of the enol. In the case of γ-lactones, it is also expected to make the ring-opening thermodynamically favored (despite the 5-membered ring) thanks to the enol/methyl ketone tautomerization. The latter process would have the additional advantage to prevent ring-opening of another lactone and, thus, avoiding branching/reticulation. The spiro structure was meant to further increase the ring-opening reactivity, as observed by Endo and Ousaka for six-membered cyclic carbonates.^38^ Access to **γSL** was envisioned by our Pd-catalyzed cycloisomerization of alkynoic acids,^39,40^ starting from malonic acid. Upon step-growth polymerization of **γSL**, we hoped to obtain not only polyesters (using diols) but also other sets of copolymers such as polythioesters (using dithiols) and polyamides (using diamines).

Here, we demonstrate that the presence of an γ-exomethylene moiety indeed makes γ-lactones prone to ring-opening and enables step-growth polymerization under mild conditions. The spiro bis-lactone **γSL** is shown to be readily accessible and to smoothly react with diols, dithiols, and diamines to afford a variety of functional regioregular (AB)_*n*_ copolymers. Model reactions were carried out and thoroughly analyzed to shed light on the reactivity of the spiro bis-lactone **γSL** and obtain reference spectroscopic data to unambiguously authenticate the structure of the copolymers obtained by step-growth polymerization. These studies revealed the occurrence of an original *trans*-lactonization process after the first ring-opening of **γSL**. This peculiar reactivity offers the possibility to prepare regioregular (ABAC)_*n*_ terpolymers, as substantiated by reacting **γSL** successively with a diol and a diamine.

## Results and Discussion

### Ring-Opening of a γ-Exovinylene Lactone with Alcohols

To evaluate the impact of the γ-exomethylene moiety on the reactivity of γ-lactones toward alcohols, we performed a kinetic study on the ring-opening of a model lactone, namely, **1b**, which was readily prepared from the corresponding alkynoic acid following a reported procedure (Scheme S1).^40,41^ Different organocatalysts (DBU, TBD, DMAP, DABCO, and MSA) were explored to promote the ring-opening reaction (Figure S1a). The most active was then used to compare the reactivity of **1b** with that of five- and six-membered lactones free of the α-exovinylene moiety (γ-BL and δ-VL, respectively), as well as with the analogous unsubstituted exovinylene lactone (**1a**) and an analogue α-exomethylene cyclic carbonate (**CC1**).

The reaction kinetics were determined by ^1^H NMR spectroscopy carrying out the ring-opening with *n-*butanol in DMF-*d*_7_ at 25 °C in the presence of the different organocatalysts (Figure S2 for the reaction with DBU as catalyst). As an example, for **1b**, the reaction progress was analyzed by the disappearance of the characteristic signals of the exocyclic double bond (δ 4.78 and 4.48 ppm), together with the diastereotopic methylene hydrogens (δ 3.42 and 3.03 ppm, C*H*_*2*_–C(CH_3_)CO_2_Et) and the appearance of the new signals corresponding to the ring-opened adduct at δ 4.11 ppm (C*H*_*2*_O–CO), 3.70 ppm (C*H*_*2*_–CO–CH_3_), and 2.17 ppm (CH_2_–CO–C*H*_*3*_), a diagnostic signal that confirms the formation of a pendant methyl ketone. Of note, complete chemoselectivity for ring-opening was observed, and no reaction occurred on the exocyclic methyl ester under these conditions. The reaction was found to be highly catalyst-dependent, with DBU being by far the most active (complete ring-opening of **1b** within ca 3 h at 5 mol % loading). The reaction with TBD was approximately 10 times slower, and no reaction was observed without a catalyst or using weak bases or acids.

**Figure 1:**
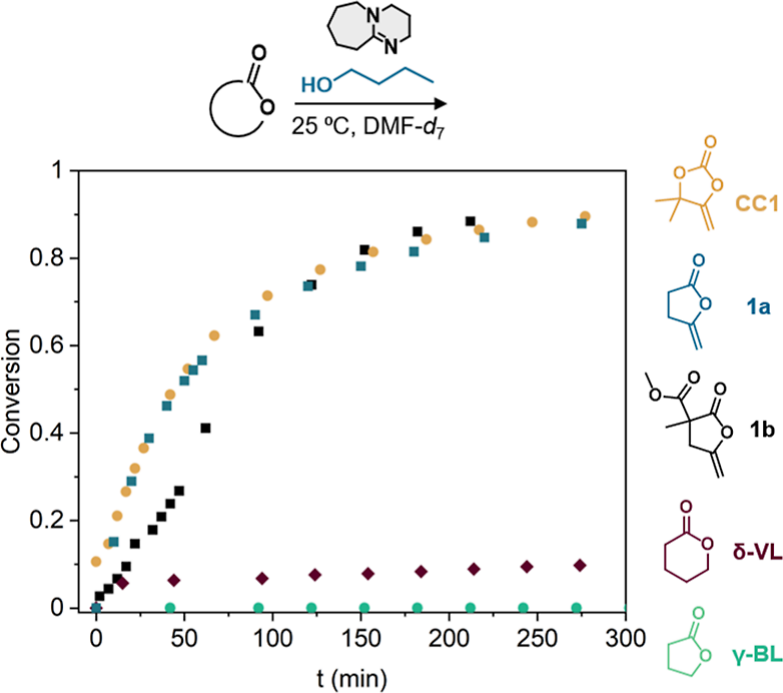
Time–conversion curves of cyclic carbonate **CC1**, 5-methylenelactone **1a**, lactone **1b**, δ-valerolactone (δ-VL), and γ-butyrolactone (γ-BL) with n-butanol with 5 mol % DBU in DMF-*d*_7_ at 25 °C.

After selecting DBU as the best catalyst for the ring-opening of **1b**, we compared its reactivity with that of γ-BL, δ-VL, **1a**, and **CC1** (Figure 1). All reactions proceeded following second-order kinetics, and the corresponding kinetic constants were determined as described in the Supporting Information (Figures S1b and S2–S6).^41^ For an initial concentration of 1 mol·L^–1^, the obtained rate constants are 1.5, 1.3, 0.5, and 0.01 mol·L^–1^.h^–1^ for **CC1**, lactone **1a**, lactone **1b**, and δ-VL, respectively. Under these conditions, γ-BL showed no conversion, in line with its thermodynamically unfavorable ring-opening (Δ*H*_p_ = −2.4 kcal.mol^–1^).^23^ Thus, the presence of the exomethylene moiety significantly enhances the reactivity of the δ-lactones, making **1a** and **1b** prone to ring-opening. The substituted lactone **1b** shows slower ring-opening kinetics than its counterpart **1a**, likely due to steric effects. Although **CC1** and **1a** react faster than lactone **1b**, the ring-opening of the three monomers reaches almost full conversion within 3 h.

### Step-Growth Polymerization of a Spiro Bis γ-Exovinylene Lactone with Diols

**Scheme 2:**
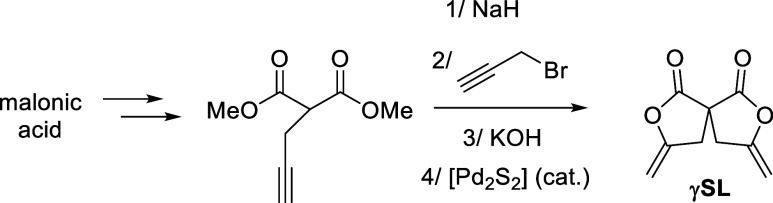
Synthesis of the Spiro Bis(γ-exomethylene γ-lactone) **γSL**

Based on the superior ring-opening reactivity observed from the model lactone **1**, we designed the bis-lactone monomer **γSL** deriving from malonic acid, a biobased precursor. After functionalization of the malonic dimethylester in the α position with propargyl bromide (Scheme 2),^41^ the corresponding bis-alkynoic acid was obtained by ester hydrolysis. Finally, double cycloisomerization catalyzed by a Pd pincer complex produced spiro bis(γ-exomethylene γ-lactone) **γSL**.

**Figure 2:**
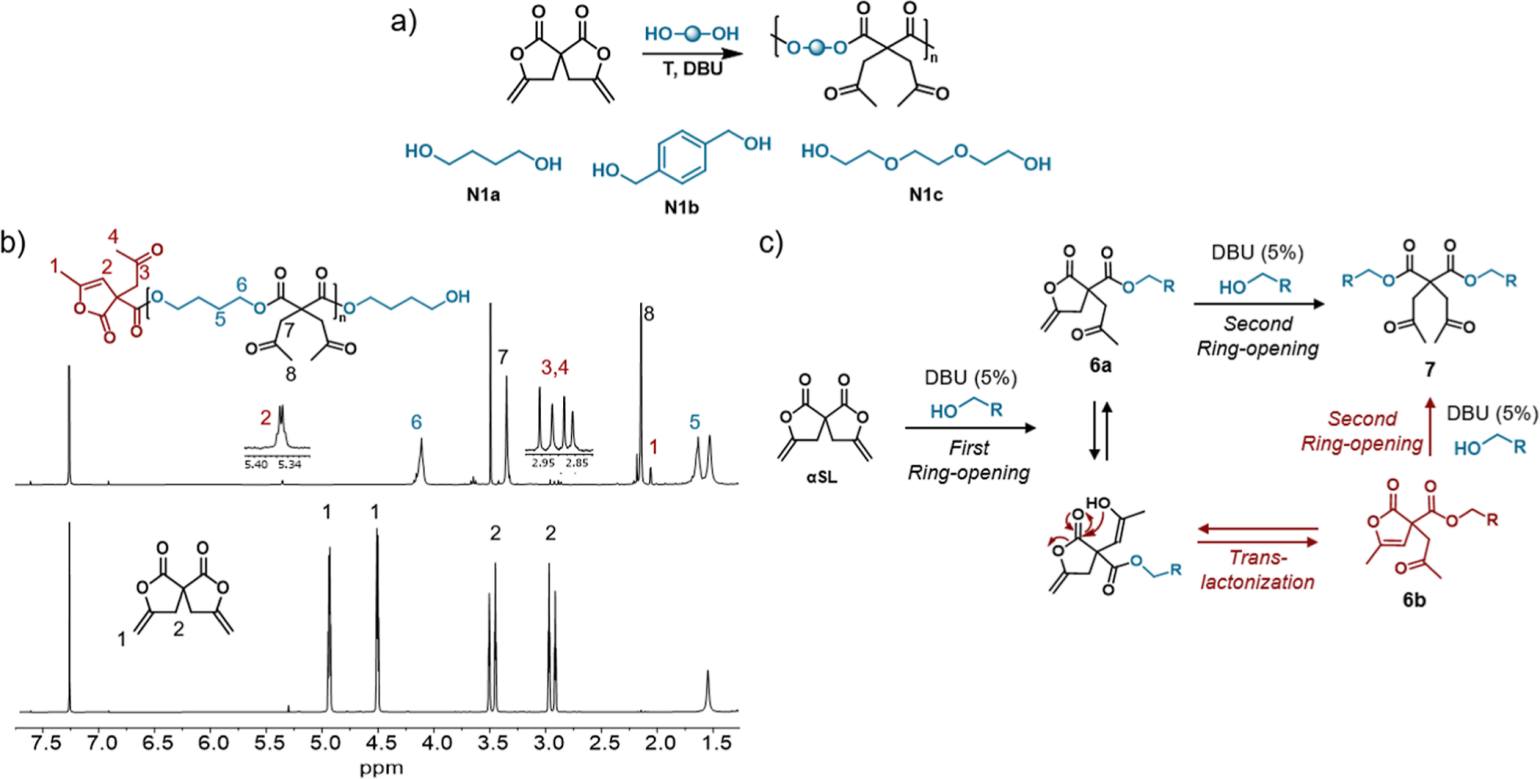
(a) Step-growth polymerization of **γSL** with different diols. (b) Stacked ^1^H NMR spectra of **γSL** (down) and polyester P(N1a) (up) (Table 1, entry 1) in CDCl_3_. (c) Tentative mechanism of ring-opening and *trans*-lactonization leading to the lactone with an endocyclic C=C double bond.

The step-growth polymerization of **γSL** with 1,4-butanediol (**N1a**) was then explored using 5 mol % of catalyst (DBU) in DMF at a concentration of 0.4 mol·L^–1^ (Table 1, entry 1, and Figure 2a). The full conversion of **γSL** and the formation of a polyester were confirmed by ^1^H NMR spectroscopy, as shown in Figure 2b. The characteristic methylene signals of butanediol ester (δ 1.63 and 4.11 ppm, e and f, respectively) together with the bis-oxopropyl residues (δ 3.35 and 2.14 ppm, g and h, respectively) confirm the successful polymerization of **γSL**. In the ^13^C NMR spectrum (Figure S8a), both ester and ketone carbons can be distinguished (signals at δ 170 and 205 ppm, respectively). Unfortunately, the obtained molecular weights were quite low (*M*_w_ = 2500 g·mol^–1^).

**Table 1:**
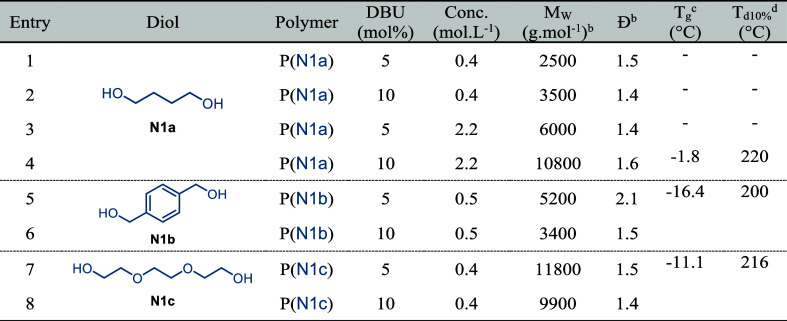
Screening of the Reaction Conditions for Polyester Synthesis with Diols^a^

a Reaction conditions: DMF, 25 °C, 24 h.

b Determined by GPC in THF and PMMA calibration.

c Determined by DSC analysis.

d Determined by TGA.

Carefully looking at the olefinic region of the ^1^H NMR spectrum, we realized that the olefinic protons shifted from δ 4.5–5.0 ppm for **γSL** to δ 5.34 ppm (signal b in Figure 2b). In addition, some unexpected signals appeared at δ ∼ 2.85–2.95 ppm (signal a). At first glance, these signals may be assigned to end groups derived from γ-exomethylene bis-lactone, but their high chemical shifts rule out this hypothesis. To better understand the outcome of the polymerization and the structure of the ensuing copolymers, we studied the reactivity of **γSL** with two equivalents of *n*-butanol as a monofunctional analogue of the diol **N1a**. The reaction was performed in DMF-*d*_7_ and monitored by NMR spectroscopy (Figures S9 and S10). Accordingly, the signals of the exomethylene unit (δ 4.5–5.0 ppm) were found to disappear immediately, indicating that the first ring-opening of the spiro bis-lactone is extremely fast. After 3 min of reaction, the double ring-opening adduct **7** (signal c in Figure S9b, bottom) is obtained in 58% yield, while the mono ring-opening adduct (**6a**, signal a) is detected in only 3% yield, with characteristic signals at δ 4.74–4.44 ppm (C=CH_2_ moiety). The isomerized lactone (**6b**, signal b), as authenticated by the signals at δ 5.46 ppm (C*H*–C=C–CH_3_) and δ 2.19 ppm (CH–C=C–C*H*_*3*_), accounts for the remaining 24%. Mechanistically, after the first ring-opening upon addition of *n*-butanol (Figure 2c), the formation of **6b** may result from a *trans*-lactonization of **6a** involving the pendant enol moiety (before it tautomerizes into methyl ketone). A double proton shift between the pendant enol and exomethylene moieties of **6a** is also conceivable, but it seems less likely as it involves an 8-membered transition state.^42^ Then, both **6a** and **6b** may react with a second molecule of *n*-butanol to give the double-addition product **7**. The lower reactivity of the lactone with the endocyclic C=C double bond, as substantiated by its presence as a terminal group, may explain the low *M*_w_ obtained.

With the aim of increasing the reactivity and obtaining higher molecular weights, we then evaluated the effects of reaction conditions. The results are summarized in Table S1.^41^ Changing the solvent (entries 1–4), reaction time (entries 7 and 8), catalyst loading (entries 6 and 8), and temperature (entries 9–10) did not significantly affect the molecular weight of the obtained copolymers. However, increasing the concentration (from 0.4 to 2.2 mol·L^–1^) induced a noticeable increase of the molecular mass up to 10,800 g·mol^–1^ when using 10 mol % DBU as catalyst (Table 1, entry 4). We hypothesize that the higher concentration has a positive impact either by increasing the reaction rate of the ring-opening of the isomerized lactone **6b** or/and by reducing the extent of *trans*-lactonization, thus favoring the direct polymerization and giving higher molecular weights.

Considering the expansion of the reaction scope with other diols, we explored 1,4-benzenedimethanol (**N1b**) and triethylene glycol (**N1c**) (Table 1). The polymerization was performed at a concentration of 0.4 mol·L^–1^ in order to compare directly with 1,4-butanediol **N1a** and avoid viscosity issues. All polymer structures were unequivocally confirmed by ^1^H and ^13^C NMR spectroscopy (Figures S7 and S8),^41^ and the molecular weights were determined by GPC in THF (Table 1 and Figure S13a). The polymerization performance observed for **N1b** led to *M*_w_ slightly greater than those observed for 1,4-butanediol, reaching molar masses around 5000 g·mol^–1^ (Table 1, entry 5). To better understand the polymerization results, we tested reactions with the model alcohols 3,5-dimethoxybenzyl alcohol (DMBA) (Figures S9 and S11) and 2-methoxyethanol (Figures S9 and S11). As *n*-butanol, the two alcohols led to instantaneous consumption of **γSL** and the formation of the isomerized lactone as an intermediate, which is also converted into the expected final diester.^43^ When comparing the reactivity of the different alcohols, 2-methoxyethanol showed faster monomer conversion than *n*-butanol and DMBA, which suggests some neighboring group effect in the rate-determining step. This result is in line with the performance observed in the polymerization reaction with **N1c**, which resulted in the highest *M*_w_ (∼10–12,000 g·mol^–1^, Table 1, entries 7 and 8). Similar neighboring group effects have been recently described for covalent adaptable networks where the participation of side nucleophiles accelerates bond formation.^44^

Overall, relatively modest molecular weights (*M*_w_ ≤ 11,800 g·mol^–1^) were obtained upon copolymerization of **γSL** with diols. At this stage, it is difficult to identify the limiting factor(s). High sensitivity to stoichiometry and parasitic reactivity of the chain ends (including backbiting) may be invoked.

### Step-Growth Polymerization with Dithiols and Diamines

The good polymerization performance observed for the reaction with diols prompted us to explore the reactivity of other nucleophiles, such as dithiols and diamines. As for the reaction with alcohols, initial studies were carried out on the model lactone **1** to explore the reaction conditions, and then with the spiro bis-lactone **γSL**, using 2 equiv of the protic nucleophile. The reaction of **1** with benzyl mercaptan required the presence of DBU as a catalyst to proceed efficiently. With 2.5 mol % of DBU, a full and clean conversion of **1** in the corresponding β-oxo-thioester **11** was observed in less than 25 min (Scheme S6).^41^ When the reaction was allowed to continue, slow evolution of this kinetic product could be observed over 48 h toward β-thioether lactone **12**, the thermodynamic product resulting from the elimination and readdition of the thiol to the exomethylene moiety instead of ring-opening of the lactone (Scheme S7). The behavior of **γSL** toward dithiols parallels that reported for **bisCC**.^35,41^

When the reaction was carried out with **γSL** and 2 equiv of benzyl mercaptan using 5 mol % of DBU (0.2 mol·L^–1^ in CDCl_3_), instantaneous ring-opening of the bis-lactone was observed (as apparent from the disappearance of the ^1^H NMR signals associated with the C=CH_2_ moiety at δ 4.50 and 4.90 ppm), to yield a mixture of products resulting from the concomitant occurrence of β-oxo-thioester into β-thioether lactone conversion of the first ring-opened motif and ring-opening of the second *exo*-methylene lactone. However, stirring the reaction for only 4 h led to the clean formation of the spiro bis(β-thioether lactone) **13** as a mixture of diastereomers (Scheme S8).^41^ Thus, the spiro structure of the bis-lactone **γSL** impacts the relative reactivity of the two lactone moieties in a way that impedes the clean formation of the bis(β-oxo-thioester), but it readily and efficiently affords the thermodynamic product, i.e., the spiro bis(β-thioether lactone), under mild conditions.

It is worth noting that, unlike what was observed for the reaction with diols, no sign of *trans*-lactonization was detected with thiols.

**Scheme 3:**
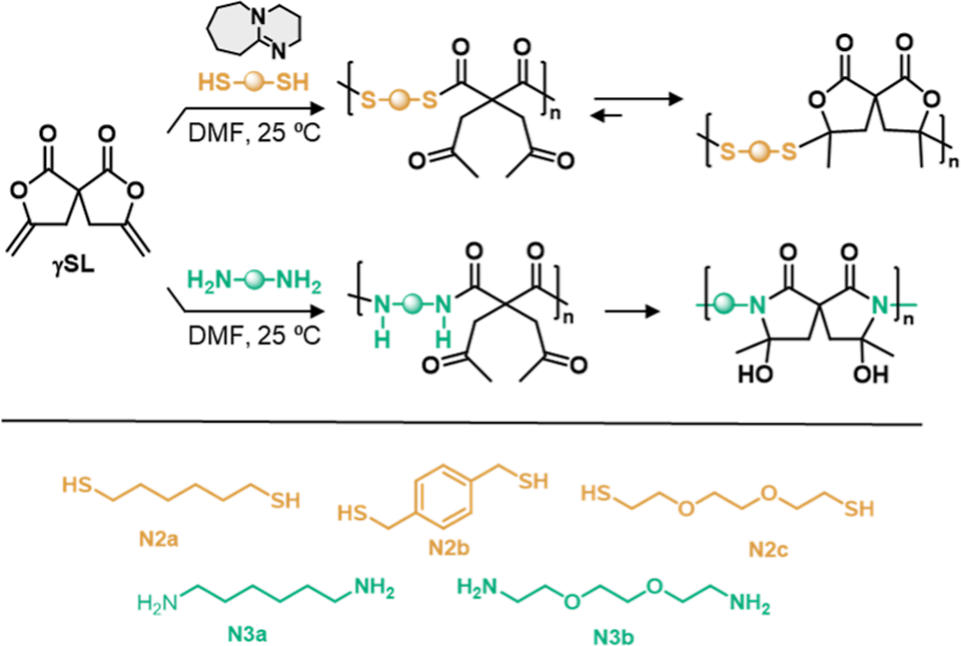
General Scheme of the Polymerizations of **γSL** with Dithiols **N2a-c** and Diamines **N3a-b**

**Figure 3:**
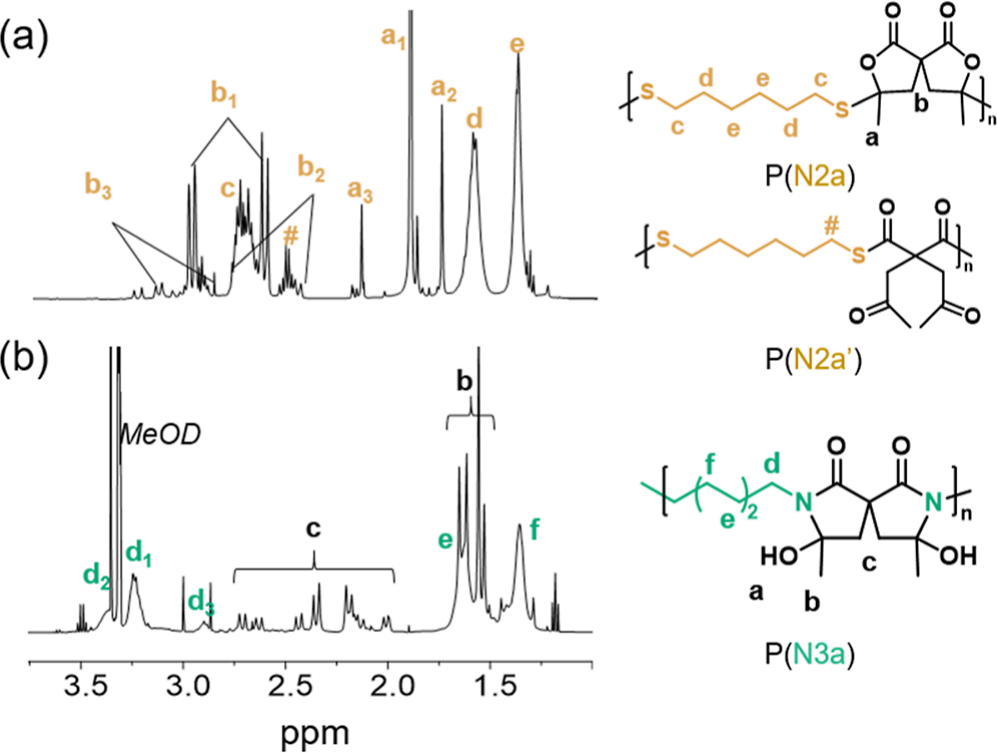
Representative ^1^H NMR spectra of polymers obtained from the step-growth polymerization of **γSL** with various nucleophiles. (a) P(**N2a**) in CDCl_3_, # corresponds to the minor poly(β-oxo-thioester) copolymer P(**N2a′**) (7% approx.) and (b) P(**N3a**) in MeOD.

In analogy with the polymerization with diols, we explored then the polymerization performance with selected aliphatic (**N2a**), aromatic (**N2b**), and ethylene glycol-derived (**N2c**) dithiols (Scheme 3 and Table 2). The polymerization conditions were selected according to the best performance observed for diols, and the results are summarized in Table 2. After 24 h of reaction, the spiro bis-lactone **γSL** was fully consumed in all cases, and the resulting copolymers were predominantly in their thermodynamic form [with spiro bis(β-thioether lactone) rather than bis(β-oxo-thioester) units] according to ^1^H NMR spectroscopy (Figures 3a and S14).^41^ For example, in **P(N2a)**, the methylene protons corresponding to the β-thioether lactone form (*c*, δ 2.65 ppm) can be clearly distinguished from the methylene proton corresponding to the thioester form (#, δ 2.50 ppm, obtained in 7% approx.) (Figure 3a). In the cases of **P(N2b)** and **P(N2c)**, the thioester form was observed in less than 3% (Figure S14). For all polymers, a mixture of stereoisomers is obtained (Figure S12, labeled protons *a*_1_, *a*_2_, and *a*_3_). The ^13^C NMR data for the three copolymers is provided in the Supporting Information (Figure S15).^41^ GPC results show similar *M*_w_ as those obtained for the related diols (Table 2 and Figure S16a). Indeed, the ethylene glycol-derived dithiol shows the highest mass, reaching 10,400 g·mol^–1^. In all cases, the dispersity is around 2 or higher and larger than those found for polyesters, probably due to the formation of thioester and thioether linkages.

**Table 2:** Copolymers Obtained from Step-Growth Polymerization of **γSL** with Dithiols **N2a–c** and Diamines **N3a–b**.^a^

entry	nucleophile	DBU (%)	*M*_W_ (g mol^–1^)^b^	*D̵*	*T*_g_^c^ (°C)	*T*_d10%_^d^ (°C)
**P(N2a)**	**N2a**	5	8200	2.4	15.7	232
**P(N2b)**	**N2b**	5	6500	1.8	4.5	235
**P(N2c)**	**N2c**	5	10,400	2.6	10.3	233
**P(N3a)**	**N3a**		e		43.3	192^f^
**P(N3a)**		5	f			
**P(N3b)**	**N3b**		10,300	2.2	–11.6	150^f^
**P(N3b)**		5	10,000	2.4		

a Reaction conditions: DMF, 25 °C, 24 h, 2 mol·L^–1^.

b Determined by GPC in DMF with LiBr with PSt calibration.

c Determined by DSC analysis.

d Determined by TGA.

e Poor solubility and measurement not possible.

f Around 20 wt % loss, followed by a plateau until 400 °C.

In marked contrast with alcohols and thiols, the ring-opening reaction with amines does not require the presence of DBU, although the reaction is rather slow at room temperature (full conversion of **1** requires 24 h in 0.2 mol·L^–1^ CDCl_3_).^41^ Clean formation of the corresponding β-oxo-amide **14** was observed, and addition of DBU (2.5 mol %) at this stage of the reaction led to the rapid formation of the β-hydroxy-lactame **15** (as a mixture of diastereomers), resulting from the intramolecular nucleophilic addition of the secondary amide moiety to the methyl ketone (Schemes S9 and S10, respectively).^34,41,45^ The reaction of **γSL** and 2 equiv of benzylamine substantiates again the impact of the spiro structure, as ring-opening of the first lactone ring occurred in less than 1 h, leading to a mixture of compounds. Again, the concomitant occurrence of the β-hydroxylactame formation for the first ring-opened motif and the partial ring opening of the second *exo*-methylene lactone may explain the mixture. Subsequent addition of DBU results in the formation of the spiro bis(β-hydroxy-lactame) **16** as a major product. Of note, when DBU was added from the beginning of the reaction, compound **16** was obtained in cleaner form within only 2 h (Scheme S11).^41^ Following the model reactions, we explored the step-growth polymerization of **γSL** with selected primary diamines: **N3a** and **N3b**. Similar conditions were employed for dithiols, and the effect of the catalyst was explored. The corresponding results are summarized in Table 2. After 24 h, the conversion of the spiro bis-lactone was complete, and the resulting copolymers were characterized by ^1^H and ^13^C NMR spectroscopy (Figures S17 and S18).^41^ In agreement with the model reactions, poly(spiro bis(β-hydroxylactames) are obtained from **N3a** and **N3b**. In both cases, different stereoisomers can be identified derived from the spiro form (Figure 3b: *d*_*1*_, *d*_*2*_, and *d*_*3*_ signals). GPC analyses could not be performed on **P(N3a)** because of a lack of solubility, but for **P(N3b)**, a relatively high *M*_w_ value was found, around 10,000 g·mol^–1^ (Figure S19a). No noticeable difference was observed between the polymerizations carried out in the absence or presence of DBU. The dispersities (*D̵* ∼ 2.3) are in agreement with the step-growth polymerization mechanism.

### Thermal Characterization of the Polymers

The thermal properties of representative samples of the three types of polymers were evaluated by thermogravimetric analysis (TGA) and differential scanning calorimetry (DSC). The results are collected in Tables 1 and 2 and Figures S13, S16, and S19. Polyesters **P(N1a-c)** showed decomposition temperatures at 10 wt % loss ranging from 200 to 220 °C. Among them, **P(N1b)**, which contains the aromatic diol, exhibited the lowest thermal stability (200 °C). Although this could be attributed to its lower molar mass, this observation is in line with previous reports on polycarbonates derived from **bisCC** that showed close *T*_d10_ values (216 °C) with the same diol.^46^ Polymers derived from dithiols, **P(N2a-c)**, showed slightly higher thermal stabilities than the polyesters (decomposition temperatures at 10 wt % loss in the range 232–235 °C). The thermal behavior of diamine-derived polymers **P(N3a,b)** is more peculiar, as a two-step degradation is observed for the two samples. A first degradation occurs at approximately 150–190 °C, followed by a plateau, and then a second degradation at around ∼400 °C. This behavior is reminiscent of that reported by Detrembleur et al. for the poly(hydroxoy-oxazolidone)s derived from **bisCC** and diamines.^45^ The first weight loss was attributed to DMF evaporation and dehydration of the hemiaminal moiety. A similar situation may explain the pattern observed for **P(N3a,b)**.

Regarding the DSC analyses, none of the copolymers exhibit crystalline behavior. The polyesters **P(N1a-c)** have lower *T*_g_ than the poly(spiro bis(β-thioether lactone)) **P(N2a-c)** (−11.1–1.8 vs 4.5–15.7 °C, respectively). Overall, the modest and different values of *M*_w_ do not enable drawing any general trend. Poly(spiro bis(β-hydroxy-lactame)) **P(N3a)** was the polymer exhibiting the highest *T*_g_ (43.3 °C), but the low solubility of the polymer prevented GPC analysis.

### Terpolymerization

**Scheme 4:**
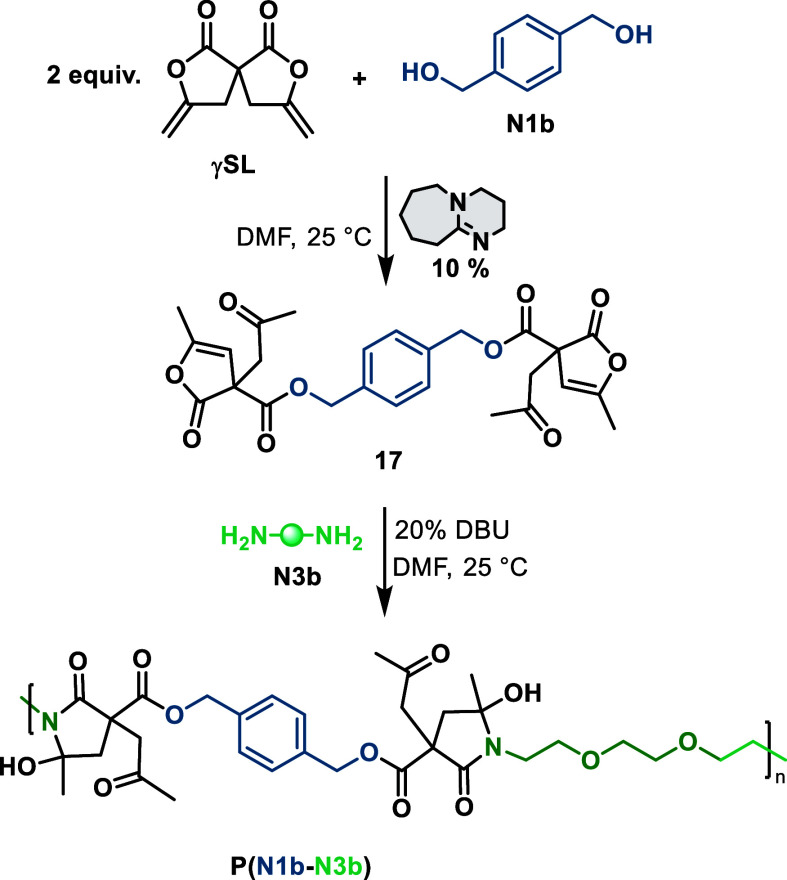
Sequential Ring-Opening of the Spiro Bis-Lactone **γSL** with Two Different Dinucleophiles, Diol **N1b** and Diamines **N3b**, to Afford a Regioregular Terpolymer

The *trans*-lactonization process evidenced in the reaction of **γSL** with diols slows the second ring-opening step, and as such, it represents a limitation, in particular, when working with aliphatic diols. However, this may be turned into an advantage if the strain imparted by the spiro structure enables selective ring-opening of one lactone. Adjusting the amount of nucleophile, i.e., using one equivalent dinucleophile for two bis-lactones, it may indeed be possible to prepare bis-lactones, which could then be engaged in step-growth polymerization with another dinucleophile to obtain (ABAC)_*n*_ terpolymers with perfect control of the comonomer sequence.^47^ The feasibility of such sequential step-growth terpolymerization was evaluated and substantiated (Scheme 4).

First, two equivalents of **γSL** were reacted with one equivalent of diol **N1b** in the presence of DBU. Gratifyingly, the corresponding bis-lactone **17** was thereby obtained and isolated (Figure S20 for ^1^H NMR spectra).^41^ Subsequent treatment with the diamine **N3b** in the presence of DBU induced step-growth terpolymerization to give a terpolymer of rather high molecular weight (*M*_w_ ∼ 18,000 g·mol^–1^, *D̵* ∼ 2.6, see Figure S21).^41^ The ^1^H and ^13^C NMR spectra show the characteristic signals for the ring-opening products of **γSL** with both **N1a** and **N3b** (Figures S22 and S23 for ^1^H and ^13^C NMR spectra),^41^ supporting the formation of a regioregular terpolymer with alternating diol and diamine moieties linked by ester-aminal units.

## Conclusions and Perspectives

In summary, we have demonstrated in this work that the bis-lactone **γSL** featuring γ-exomethylene groups and a spiro structure displays enhanced reactivity toward protic nucleophiles such as alcohols, thiols, or primary amines at room temperature. Using bifunctional comonomers, step-growth polymerization becomes achievable under mild conditions, leading to regioregular (AB)_*n*_ copolymers with diverse linkages and functional groups. Compared to ROP, this approach enables the preparation of a broad variety of copolymers from a unique bis-lactone monomer, **γSL**.

With diols as comonomers and DBU as organocatalyst, polyesters featuring methyl ketones as pendant groups were obtained, with *M*_w_ ranging from 5000 to 10,000 g·mol^–1^. Thorough reaction monitoring revealed the occurrence of a *trans*-lactonization process after the first ring-opening of **γSL**. This is detrimental to the polymerization behavior but could be mitigated by increasing the reaction concentration or using nucleophiles prone to neighboring-group effects, which react faster. Dithiols (stronger and softer nucleophiles than diols) show higher reactivity than diols toward **γSL** and enable, in the presence of DBU, the selective preparation of poly(spiro bis(β-thioether lactones)) as the thermodynamic products. Moreover, the step-growth copolymerization of **γSL** with primary diamines proceeds equally well with and without the DBU catalyst, affording poly(spiro bis(β-hydroxy-lactame)) with *M*_w_ of about 10,000 g·mol^–1^. Remarkably, the *trans*-lactonization process could be leveraged to access a regioregular (ABAC)_*n*_ terpolymer by sequential ring-opening of **γSL** with a diol and a diamine.

This last point is probably the main difference between **γSL** and **bisCC**. The spiro structure of **γSL** and the *trans*-lactonization process it undergoes desynchronize the ring opening of the two rings with alcohols, enabling selective preparation and isolation of the mono ring-opened products. On the contrary, **γSL** and **bisCC** behave similarly toward dithiols and diamines. The poly(oxo-thioesters) and poly(oxo-amide)s resulting from the reaction of **γSL** with dithiols and diamines (with DBU as catalyst) cannot be isolated, and only the thermodynamic forms, poly(bis(β-thioether lactone)) and poly(bis(β-hydroxy-lactame)) could be isolated.

Overall, the incorporation of γ-exomethylene groups was shown to enable the step-growth polymerization of bis-lactones under mild conditions. Accordingly, bis(γ-exomethylene γ-lactones) stand as readily accessible and very powerful monomer platforms for accessing a broad range of functional (AB)_*n*_ copolymers and even (ABAC)_*n*_ terpolymers. While the spiro structure was initially meant to enhance further the reactivity toward ring-opening, the results obtained with the model monolactones **1a**–**b** show that the γ-exomethylene substitution is enough to achieve ring-opening at room temperature. Thus, the approach developed here is certainly generalizable to bis(γ-exomethylene γ-lactones) without a spiro junction but a tether between the two rings. Future studies in our groups will explore such a possibility and aim to further develop the terpolymerization approach.

## Supporting Information Available

Experimental procedures and spectral data (PDF)
