# Supplementary material for: Boosting the Reactivity of Bis-Lactones to Enable Step-Growth Polymerization at Room Temperature
Source: Macromolecules. 2024 Mar 22;57(7):3319–27. doi: 10.1021/acs.macromol.3c02527 (PMC11008534; doi:10.1021/acs.macromol.3c02527)
Supplement: Supplementary file 1 — ma3c02527_si_001.pdf [file ma3c02527_si_001.pdf]

Supporting Information

Boosting the Reactivity of Bis-lactones to Enable Step-Growth Polymerization at Room Temperature

Marta Ximenis,[†,§] Julien Monot,[‡,§] Elena Gabirondo,[†] Janna Jeschke,[†] Blanca Martín-Vaca,*[‡] Didier Bourissou,*[‡] Haritz Sardon*[†]

[†] *POLYMAT, University of the Basque Country UPV/EHU, Joxe Mari Korta Center Avda. Tolosa 72, 20018 Donostia-San Sebastian, Spain.*

[‡] *Laboratoire Hétérochimie Fondamentale et Appliquée (UMR 5069), Université de Toulouse (UPS), CNRS, 118 route de Narbonne, F-31062 Toulouse, France.*

Table of contents

1 Materials S3

2 Characterization methods S3

3 Experimental procedures S4

3.1 Synthesis of the disubstituted γ-methylene-γ-lactone (**1b**) and spiro-bislactone (**γSL**) S4

3.2 Kinetic studies of model reactions with *n*-butanol S6

3.3 Synthesis and characterization of the polyesters S9

3.4 Model reactions of the bislactone **γSL** and monofunctional alcohols S11

3.5 Model reactions of **1** or bislactone **γSL** and monofunctional thiols and amines S15

3.6 Synthesis and characterization of the poly(spiro-bis(β-thioether lactone))s and poly(spiro-bis(β-hydroxy-lactame))s S19

3.7 Synthesis and characterization of an $(ABAC)_n$ terpolymer S22

4 NMR spectra of the molecular compounds S25

1. Materials

All reactions and manipulations were carried out under argon atmosphere using standard Schlenk techniques unless otherwise stated. Dry, oxygen–free solvents were employed. All organic reagents were obtained from commercial sources and used as received or prepared from known literature procedures. The complex $[(SCS)^{iPr}Pd]_2$ and lactone **1a** were synthesized using the method previously reported.[1] Catalyst loadings are given relative to the effective Pd content.

2. Characterization methods

Nuclear magnetic resonance (NMR) spectroscopy. ^{1}H and ^{13}C spectra were obtained on a Bruker Avance 300, 400 or 500 MHz. Chemical shifts are given in ppm relative to residual solvent as an internal standard. Unless otherwise stated, NMR spectra were recorded at 293 K.

Gel permeation chromatography (GPC). Gel permeation chromatography (GPC) was performed on a Waters equipment provided with refractive index (RI) and ultraviolet (UV) detectors. For this, 100 μL of 0.1 (wt/vol) sample solution in THF or DMF with LiBr (depending on the polymer structure) was injected and the analysis was performed at a flow-rate of 0.5 mL min^{-1}. HR5E and HR2 Waters linear Styragel columns (7.8 mm × 300 mm, pore size 103 –104 Å) packed with cross-linked polystyrene (PS) and protected with a pre-column were used. Molar mass averages and distributions were calculated against poly(methyl methacrylate) (PMMA) standards.

High Resolution Mass Spectrometry (HRMS): Mass spectra were recorded on a Waters GCT premier apparatus and Waters Xevo G2 QTOF apparatus.

Differential scanning calorimetry (DSC). DSC analyses were performed with DSC 25 TA instruments under Nitrogen atmosphere at a heating and cooling rate of 10 ºC.min^{-1}. Measurements were performed, depending on the sample, in a range of temperature from -80 ºC to 150 ºC.

Thermogravimetric analyses (TGA). TGAs were performed with a TGA/Q500 TA instrument under Nitrogen atmosphere in a platinum crucible at a heating rate of 10 ºC.min-1 from 25 ºC to 800 ºC.

[1] N. Á. Espinosa-Jalapa, D. Ke, N. Nebra, L. Le Goanvic, S. Mallet-Ladeira, J. Monot, B. Martin-Vaca, D. Bourissou, *ACS Catal.* 2014, **4**, 3605–3611.

3. Experimental procedures

3.1 Synthesis of the disubstituted γ-methylene-γ-lactone (1b) and spiro-bislactone (γSL)

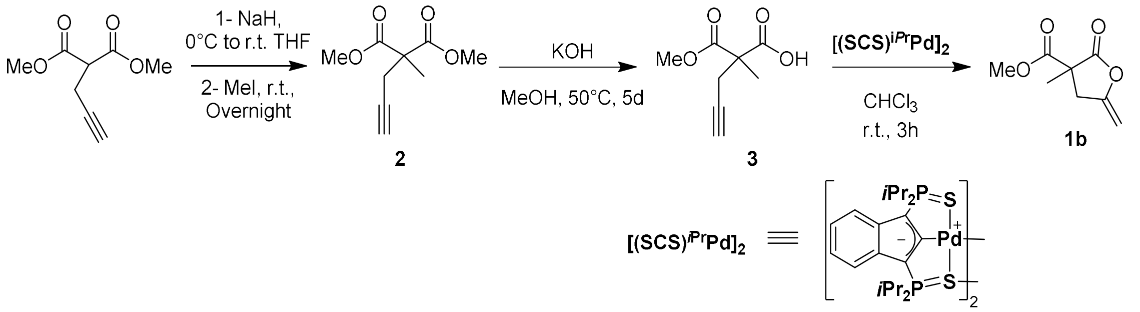

Scheme S1. Preparation of lactone γ-methylene-γ-lactone **1**

Preparation of **2**. To a suspension of NaH (472 mg, 11.8 mmol, 1 equiv.) in THF (30 mL), Dimethyl propargylmalonate (1.79 mL, 11.8 mmol) was added dropwise at 0 °C. After stirring for 30 min while warming to rt, the suspension was cooled to 0 °C and iodomethane (1.1 equiv) was added at this temperature. The reaction mixture was stirred while being allowed to warm up to r.t. overnight. Then, solvent was evaporated and the residue was dissolved in ethyl acetate (20 mL), washed with water (15 mL), brine (15 mL) and dried over Na_2SO_4. After filtration, the solvents were removed in vacuo. The crude product was purified by column chromatography (Pentane/EtOAc: 95/5) affording **2** as a pure colorless oil (1.185 g, 55%).

^{1}H NMR (300 MHz, Chloroform-*d*) δ 3.74 (d, J = 0.9 Hz, 6H), 2.79 (dd, J = 2.7, 0.9 Hz, 2H), 2.01 (d, J = 2.7 Hz, 1H), 1.56 (s, 3H). All the spectroscopic data were in accordance with the literature.[2]

Preparation of **3**. In a round bottom flask, **2** (1.185 g, 6.43 mmol, 1.0 equiv.) and KOH (397 mg, 7.1 mmol, 1.1 equiv.) were dissolved in MeOH (13 mL) and stirred at 50°C for 5 days. Aqueous saturated $NaHCO_3$ solution (20 mL) was added and the aqueous phase was washed with Et_2O (3×20 mL) and acidified with HCl (aq., 5 M) to pH = 1. CH_2Cl_2 (20 mL) was added and the organic phase was separated. The aqueous phase was extracted with CH_2Cl_2 (2×20 mL), the combined organic phases were dried over Na_2SO_4 and the solvent was removed in vacuo. Product **3** (831 mg, 76%) was obtained as a white solid without any further purification.

[2] E. Benedetti, A. Simonneau, A. Hours, H. Amouri, A. Penoni, G. Palmisano, M. Malacria, J.-P. Goddard, L. Fensterbank, *Adv. Synth. Catal.* 2011, **353**, 1908-1912.

^{1}H NMR (300 MHz, Chloroform-*d*) δ 3.77 (s, 3H), 2.93 – 2.68 (m, 2H), 2.05 (t, J = 2.7 Hz, 1H), 1.58 (s, 3H). All the spectroscopic data were in accordance with the literature.[3]

*Preparation of **1b**.* Alkynoic acid **3** (831 mg, 4.88 mmol) was added to a solution of $[(SCS)^{iPr}Pd]_2$complex**Erreur ! Signet non défini.** (25 mg, 0.5%mol) in chloroform (35 mL). The reaction was stirred at room temperature for 3h. Then, the reaction mixture was concentrated and the residue was purified by flash chromatography (Pentane/EtOAc: 80/20) to afford **1b** as a pure colorless oil (820 mg, 99%).
^{1}H NMR (300 MHz, Chloroform-*d*) δ 4.82 (q, J = 2.3 Hz, 1H), 4.38 (q, J = 2.3 Hz 1H), 3.78 (s, 3H), 3.36 (dt, J = 16.4, 1.9 Hz, 1H), 2.76 (dt, J = 16.4, 2.0 Hz, 1H), 1.57 (s, 3H). All the spectroscopic data were in accordance with the literature.[4]

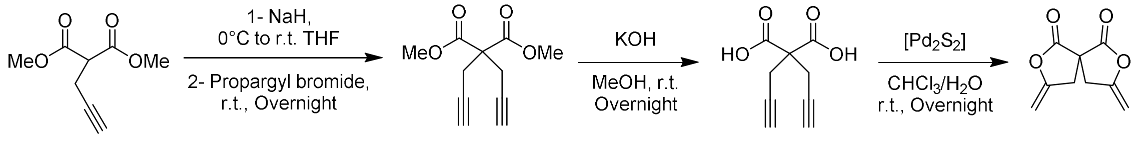

4
5
γSL

Scheme S2. Preparation of lactone spiro-bislactone **γSL**

*Preparation of **4**.* To a suspension of NaH (472 mg, 11.8 mmol, 1 equiv.) in THF (30 mL), Dimethyl propargylmalonate (1.79 mL, 11.8 mmol) was added dropwise at 0 °C. After stirring for 30 min while warming to rt, the suspension was cooled to 0 °C and Propargyl bromide (2.62 mL, 80% in toluene, 2 equiv.) was added at this temperature. The reaction mixture was stirred while being allowed to warm up to r.t. overnight. Then, solvent ere evaporated and the residue was dissolved in ethyl acetate (20 mL), washed with water (15 mL), brine (15 mL) and dried over Na_2SO_4. After filtration, the solvents were removed in vacuo. The obtained solid was suspended in pentane, filtered and washed three times with pentane to afford pure product **4** (1.36g, 55%) as a white solid.
^{1}H NMR (300 MHz, Chloroform-*d*) δ 3.77 (s, 6H), 3.00 (d, J = 2.7 Hz, 4H), 2.04 (t, J = 2.6 Hz, 2H). All the spectroscopic data were in accordance with the literature.[5]

*Preparation of **5**.* In a round bottom flask, compound **4** (1.36 g, 1.0 equiv., 6.53 mmol) was dissolved in methanol (17 mL). An aqueous solution of potassium hydroxide (1.83

[3] A. Arcadi, A. Burini, S. Cacchi, M. Delmastro, F. Marinelli, B. Rosa Pietroni, *J. Org. Chem.* 1992, **57**, 3, 976–982.
[4] E. Tomás-Mendivil, P. Y. Toullec, J. Díez, S. Conejero, V. Michelet, V. Cadierno, *Org. Lett.* 2012, **14**, 10, 2520–2523
[5] A. S. K. Hashmi, T. Häffner, M. Rudolph, F. Rominger, *Chem. Eur. J.* 2011, **17**, 8195–8201.

g, 5.0 equiv.) was prepared with a minimal amount of water and added to the methanol solution. The reaction was allowed to stir overnight at room temperature. Then, the reaction was concentrated in vacuo to dryness. The residue was dissolved in HCl 3M and the aqueous phase was washed DCM (3 × 30 mL). The aqueous phase was, then, evaporated until dryness. The residue was extracted with MeOH from the KCl salt, then, concentrated and extracted a second time with DCM. DCM was finally evaporated to yield the di-acid **5** as a pure white solid (900 mg, 76%) and was used without further purification.

^{1}H NMR (300 MHz, Deuterium Oxide) δ 7.18 (s, 4H), 5.23 (s, 2H). All the spectroscopic data were in accordance with the literature.[6]

Preparation of **γSL**. **5** (900 mg, 5 mmol) was dissolved in a biphasic medium $CHCl_3/H_2O$ (10mL/10mL). $[(SCS)^{iPr}Pd]_2$ complex[1] was added and the reaction mixture was heavily stirred overnight. Then, the orange organic phase was separated and the aqueous phase was extracted with DCM (2 × 10 mL). The combined organic phase were concentrated in vacuo to yield an orange solid. This residue was dissolved in a minimum of DCM and then pentane was added to render a white precipitate. The solid was filtered and washed with pentane to afford the spiro dilactone **γSL** as a pure white solid (850 mg, 94%).

^{1}H NMR (300 MHz, Chloroform-d) δ 4.93 (dt, J = 3.2, 2.0 Hz, 2H), 4.50 (dt, J = 3.3, 1.8 Hz, 2H), 3.47 (dt, J = 16.6, 1.8 Hz, 2H), 2.94 (dt, J = 16.6, 2.0 Hz, 2H). All the spectroscopic data were in accordance with the literature.[6]

3.2 Kinetic studies of model reactions with n-butanol

All model reactions were carried out at 25 °C in DMF-d_7 and monitored by ^{1}H NMR spectroscopy. In a representative experiment, in a glovebox, 0.6 mmol of lactone and 0.6 mmol of *n*-butanol were added to a reaction tube. The reactants were solubilized in 0.5 ml of anhydrous DMF and 0.1 ml of a DBU (or the corresponding catalyst) solution in DMF (C = 0.6 M) was added to the reaction medium (0.03 mmol, 0.05 eq).and spectra were recorded at different reaction times.

The second-order kinetics were calculated as the following: $-d[\mathbf{1}]/dt = k[\mathbf{1}]^2$, (where [**1**] = [lactone] = [*n*-butanol]), which was confirmed by the linear relationship between t and 1/(1 − p), being *t* the time and *p* the fraction of the ring-opened adduct. Rate constants, *k*, were calculated for each kinetic model.

[6] J. Alemán, V. del Solar, C. Navarro-Ranninger, *Chem. Commun.* 2010, **46**, 454-456

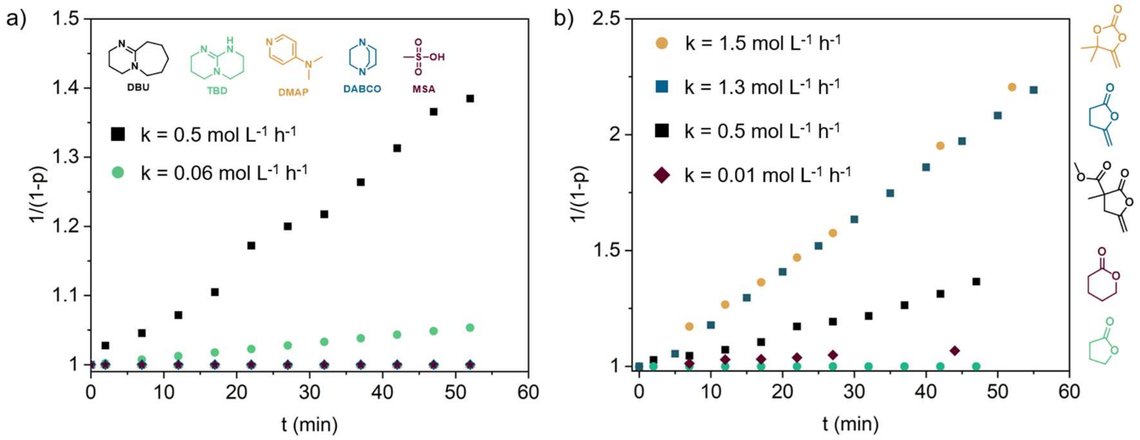

Figure S1. a) Alcoholysis of 1 M α-exovinylene lactone **1b** with *n*-butanol and 5% catalyst in DMF-d_7 at 25 ºC. b) Kinetics plot from the alcoholysis of various lactones and a related cyclic carbonate with *n*-butanol and 5% DBU in DMF-d_7 at 25 ºC, 1M.

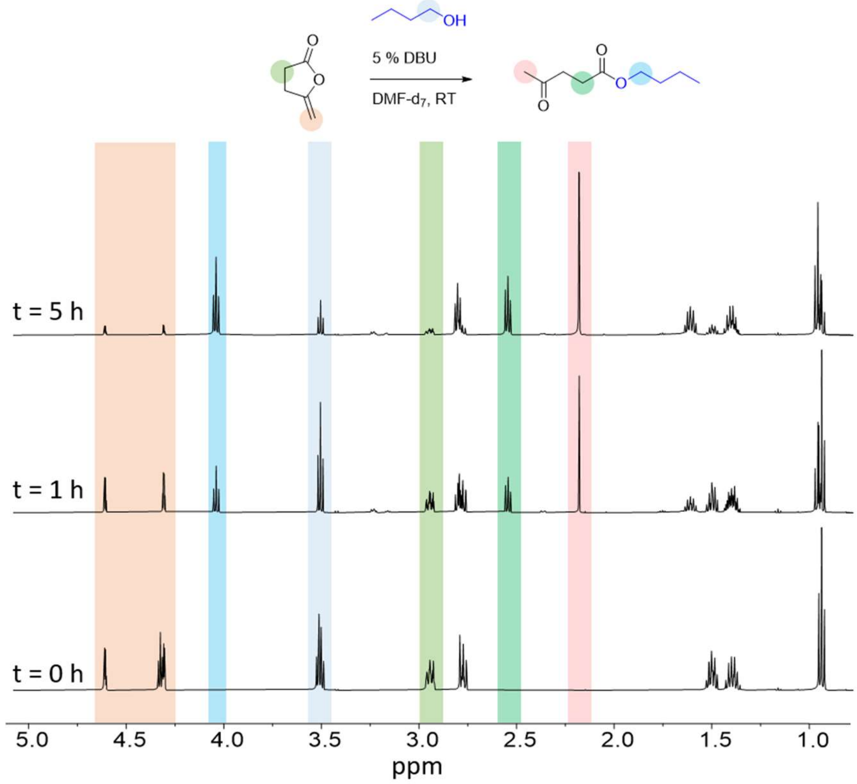

Figure S2. ^{1}H-NMR evolution over time of alcoholysis of 1 M 5-methylene lactone **1a** with *n*-butanol (25 ºC). *For t = 0 no catalyst is added.

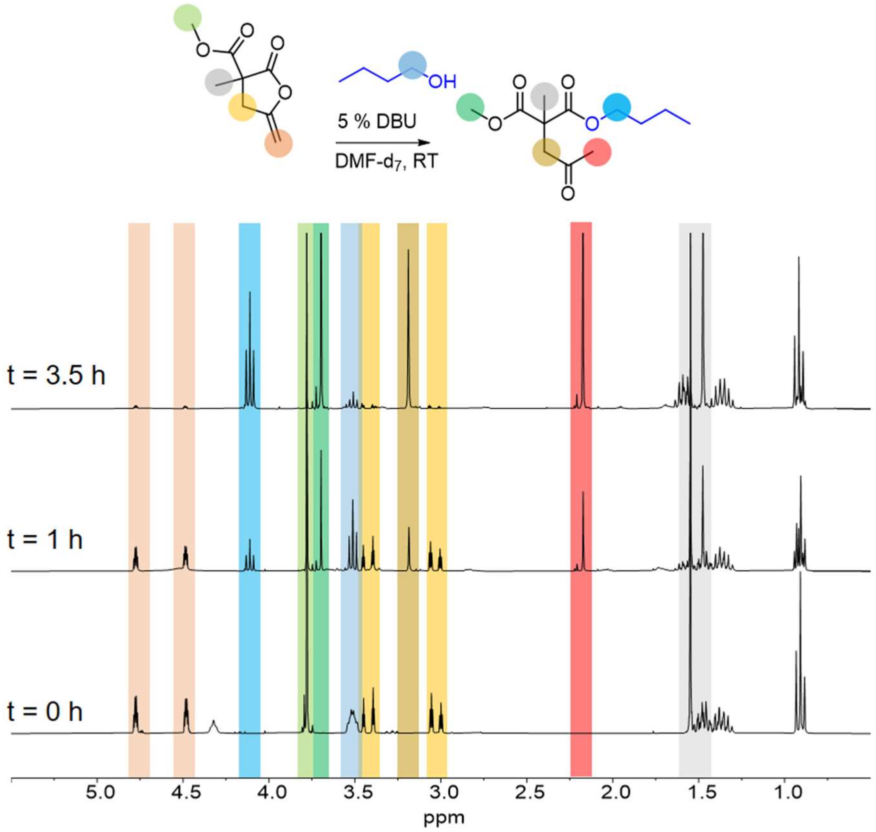

Figure S3. ^{1}H-NMR evolution over time of alcoholysis of 1 M lactone **1b** with *n*-butanol (25 ºC).*For t = 0 no catalyst is added.

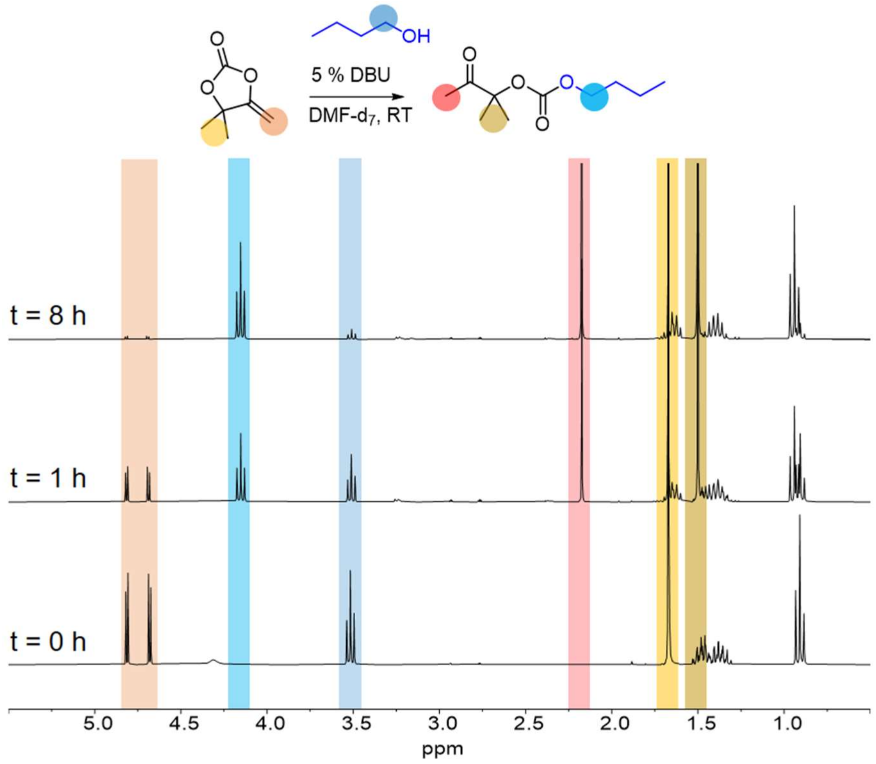

Figure S4. ^{1}H-NMR evolution over time of alcoholysis of 1 M cyclic carbonate **CC1** with n-butanol (25 ºC). *For t = 0 no catalyst is added.

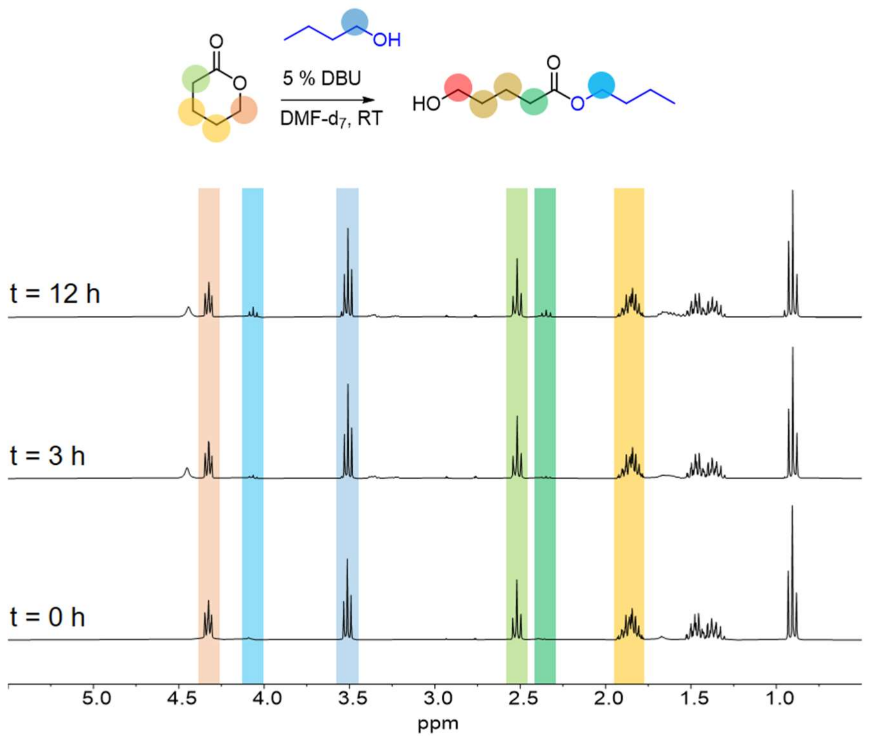

Figure S5. ^{1}H-NMR evolution over time of alcoholysis of 1 M δ-valerolactone (**δ-VL**) with *n*-butanol (25 ºC). *For t = 0 no catalyst is added.

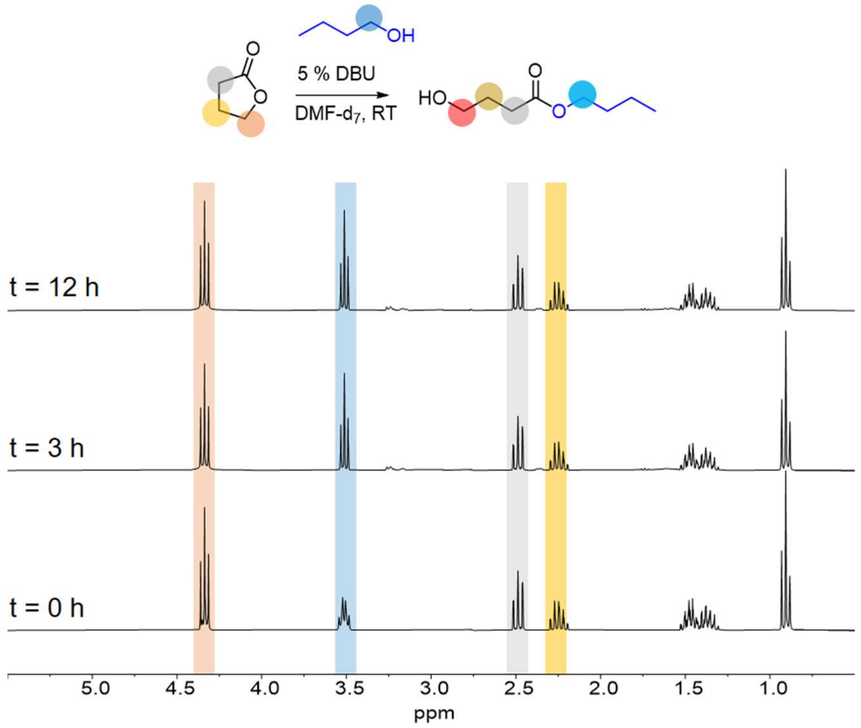

Figure S6. ^{1}H-NMR evolution over time of alcoholysis of 1 M γ-butyrolactone (**γ-BL**) with *n*-butanol (25 ºC). *For t = 0 no catalyst is added.

3.3 Synthesis and characterization of the polyesters

All reactions were carried out inside an N_2-filled glove box. 45 mg (0.25 mmol, 1 eq.) of **γSL** and the corresponding diol were added to a glass vial. Next, 30 or 60 µL of a DBU solution (76 mg/mL, DMF or $CHCl_3$, 5 % or 10 % DBU) were added, and DMF or $CHCl_3$ was added until a final volume of 0.75 mL (0.4 M) or 0.12 mL (2.2 M). After stirring for 24 h at 25 °C, an aliquot was taken out for conversion calculation and then precipitated in H_2O (from DMF) or hexanes (from $CHCl_3$). The polymers were precipitated two times, isolated by centrifugation and dried under a vacuum.

Table S1. Screening of the reaction conditions for polyester synthesis with diols.

Entry	Polymer	Solvent	DBU (%)	Temp. (º C)	Time (h)	Conc. (mol L^{-1})	MW (g mol^{-1})[a]	*Đ*
1	P(N1a)	DMF	5	25	24	0.4	2500	1.5
2	P(N1a)	DMF	10	25	24	0.4	3500	1.4
3	P(N1a)	$CHCl_3$	5	25	24	0.4	2700	1.4
4	P(N1a)	$CHCl_3$	10	25	24	0.4	2200	1.6
5	P(N1a)	DMF	5	25	24	2.2	6000	1.4
6	P(N1a)	DMF	10	25	24	2.2	**10800**	1.6
7	P(N1a)	DMF	5	25	48	2.2	6900	1.4
8	P(N1a)	DMF	10	25	48	2.2	**10400**	1.5
9	P(N1a)	DMF	5	60	24	0.4	2800	1.2
10	P(N1a)	DMF	10	60	24	0.4	2500	1.3
11	P(N1b)	DMF	5	25	24	0.4	5200	2.1
12	P(N1b)	DMF	10	25	24	0.4	3400	1.5
13	P(N1b)	$CHCl_3$	5	25	24	0.4	5900	1.3
14	P(N1b)	$CHCl_3$	10	25	24	0.4	6700	1.2
15	P(N1c)	DMF	5	25	24	0.5	**11800**	1.5
16	P(N1c)	DMF	10	25	24	0.5	9900	1.4
17	P(N1c)	$CHCl_3$	5	25	24	0.5	**16600**	1.5
18	P(N1c)	$CHCl_3$	10	25	24	0.5	**11700**	1.4

[a] Determined by GPC in THF with PMMA calibration

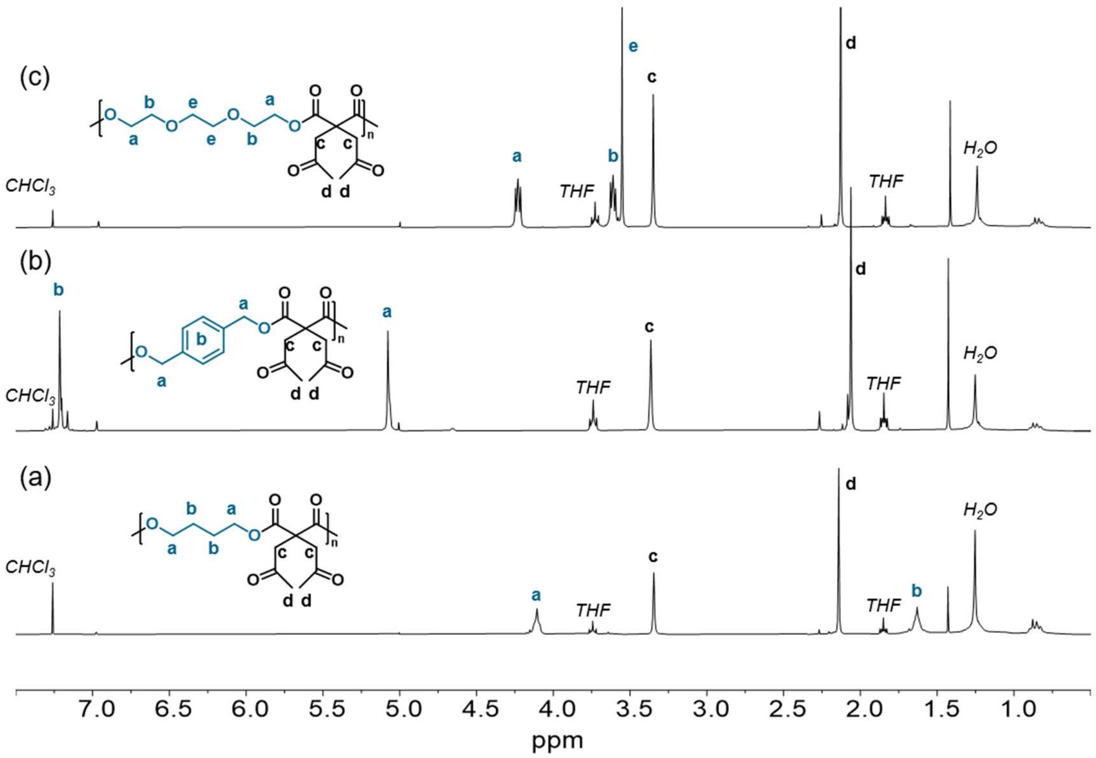

Figure S7. Stacked ^{1}H NMR spectra of P(N1a-c) in $CDCl_3$, purified by precipitation.

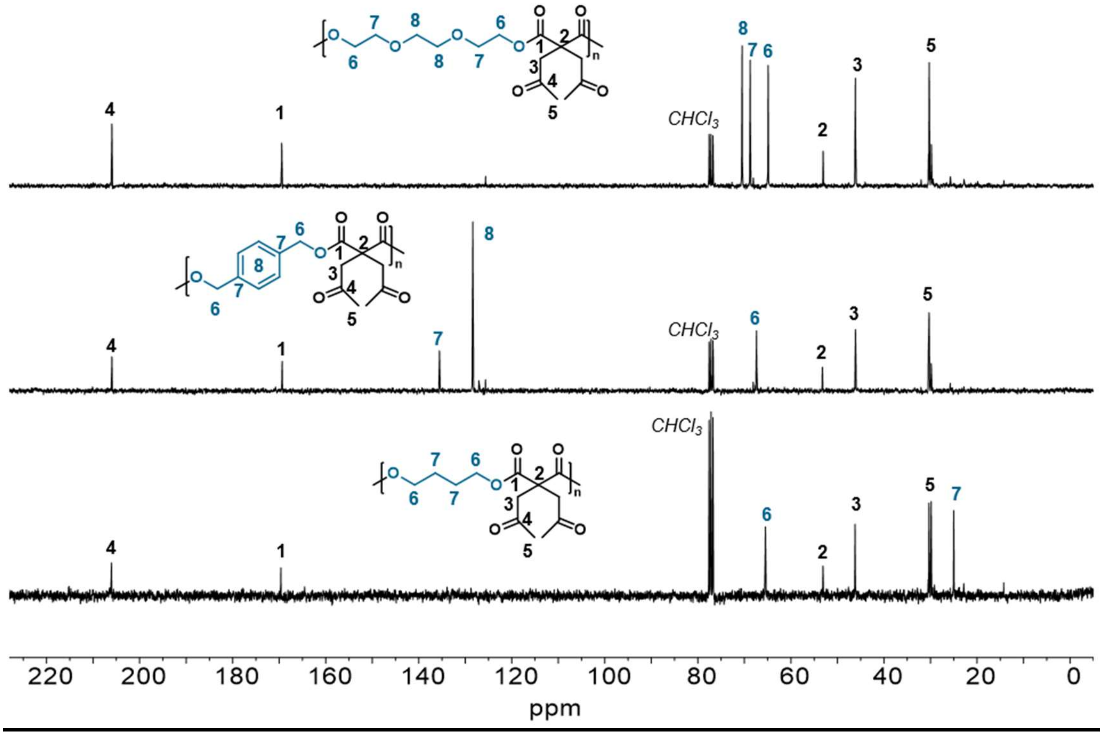

Figure S8. Stacked ^{13}C NMR spectra of P(N1a-c) in $CDCl_3$, purified by precipitation.

3.4 Model reactions with dilactone monomer (γSL) and monofunctional alcohols

The model reaction was carried out at 25 °C in DMF-d_7 and monitored by ^{1}H NMR spectroscopy. In a glovebox, 0.6 mmol of **γSL** and 1.2 mmol of the monoalcohol (i.e. *n*-butanol) were added in a reaction tube. The reactants were solubilized in 0.5 ml of anhydrous DMF and 0.1 ml of a DBU solution in DMF (C = 0.6 M) was added to the reaction medium (0.03 mmol, 5 %) and spectra were recorded at different reaction times.

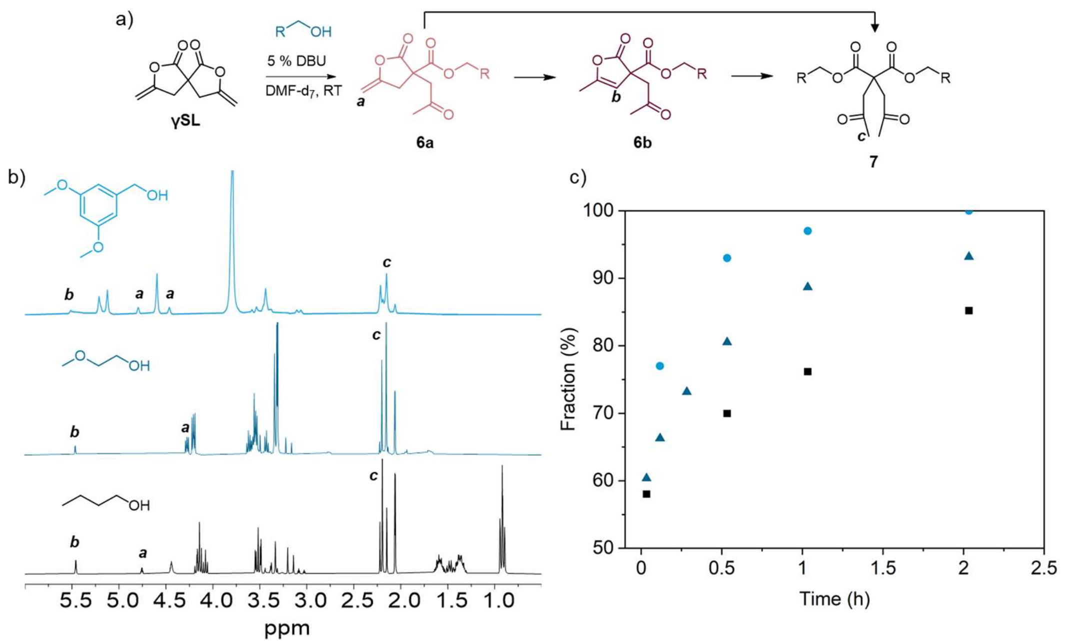

Figure S9. a) Scheme of the ring-opening of 1 M **γSL** with 2 equivalents of different monotopic alcohols using 5 % DBU at 25 ºC in DMF-d_7. b) Representative example of ^{1}H-NMR spectra of the reaction recorded at t = 3 min, and corresponding assignation of the key signals for each reaction product. c) Time-conversion curves for the reaction of **γSL** with each alcohol (Fracton of final adduct **7**). Magnification from 50-100 % of conversion to final product in the first 2.5 h of reaction time (● 2-methoxyethanol; ▲ 3,5-dimethoxybenzyl alcohol; ■ *n*-butanol).

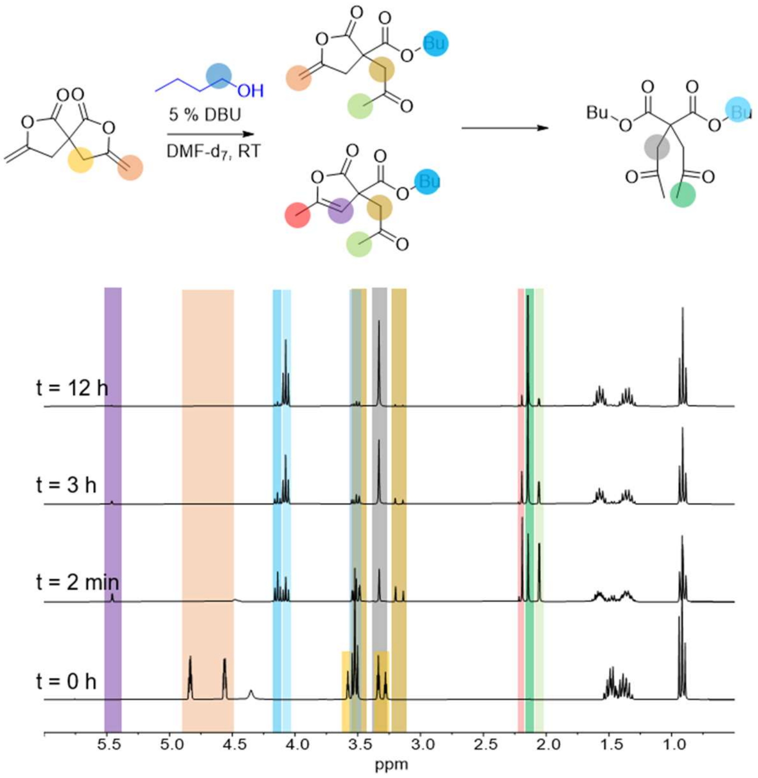

Figure S10. ^{1}H-NMR evolution over time of alcoholysis of 1 M **γSL** with *n*-butanol (25 ºC). *For t = 0 no catalyst is added.

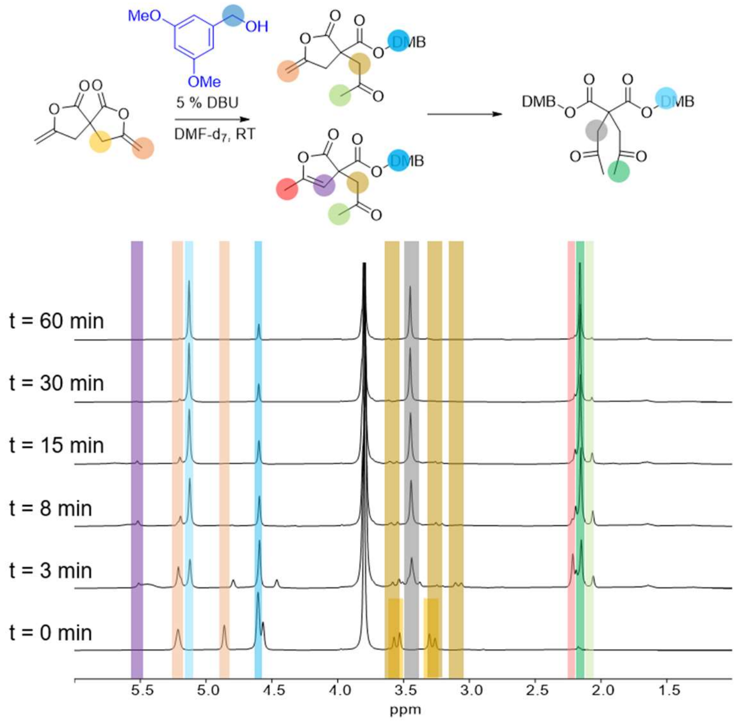

Figure S11. ^{1}H-NMR evolution over time of alcoholysis of 1 M **γSL** with DMBA (25 ºC). *For t = 0 no catalyst is added.

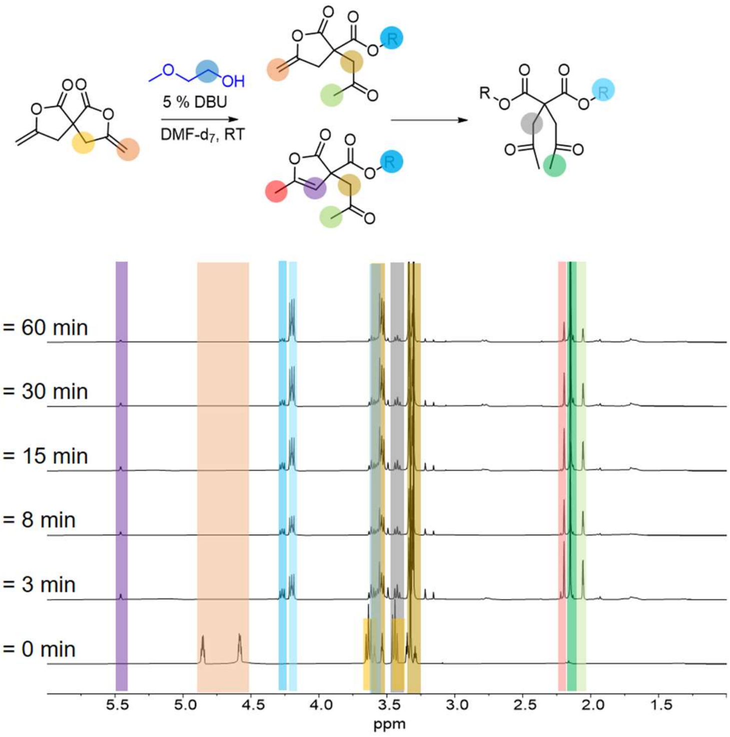

Figure S12. ^{1}H-NMR evolution over time of alcoholysis of 1 M **γSL** with 2-methoxyethanol (25 ºC). *For t = 0 no catalyst is added.

General procedure for the stoichiometic Reactions with 3,5-dimethoxybenzyl alcohol. In a glovebox, **1** or **γSL** (1 equiv.) and alcohol (3,5-dimethoxybenzyl alcohol) (1 or 2 equiv.) were added in a schlenk tube. The reactants were solubilized anhydrous $CDCl_3$ ([C] = 0.2 M) and a DBU solution (2.5 or 5 %mol) in $CDCl_3$ (C = 0.063 M) was added to the reaction medium and the reaction was stirred at 25 °C.

1-(3,5-dimethoxybenzyl) 3-methyl 2-methyl-2-(2-oxopropyl)malonate (8)

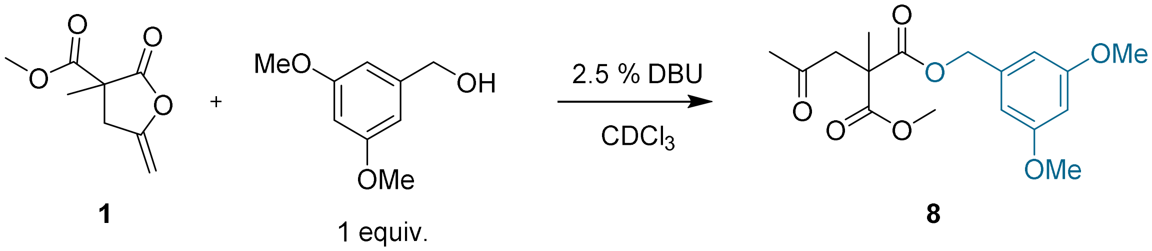

1 **8**

Scheme S3. Reaction of lactone **1** with 3,5-dimethoxybenzyl alcohol

The reaction was monitored by ^{1}H NMR and after 2 h, full conversion was observed. Then, the reaction mixture was concentrated and purified via flash chromatography (Pentane: Ethyl acetate: 80/20) to afford 96 mg of a colorless oil (96% Yield).

^{1}H NMR (300 MHz, $CDCl_3$) δ 6.45 (d, *J* = 2.3 Hz, 2H), 6.40 (d, *J* = 2.3 Hz, 1H), 5.10 (bs, 2H), 3.78 (s, 6H), 3.70 (s, 3H), 3.12 (s, 2H), 2.14 (s, 3H), 1.55 (s, 3H).
^{13}C NMR (75.5 MHz, $CDCl_3$) δ 204.0, 171.9, 170.6, 161.0, 138.0, 105.7, 100.2, 67.1, 55.5, 52.9, 51.6, 48.9, 30.4, 20.6.
HRMS: (ESI+) calculated for $C_{17}H_{23}O_7$: 339.1444; found: 339.1448.

3,5-dimethoxybenzyl-5-methyl-2-oxo-3-(2-oxopropyl)-2,3-dihydrofuran-3-carboxylate (9b)

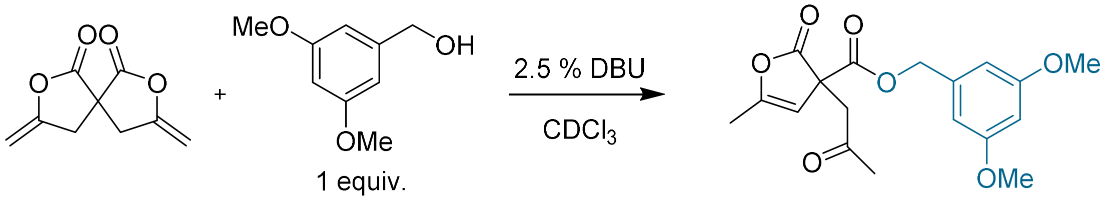

γSL **9b**

Scheme S4. Reaction of lactone **γSL** with 1 equivalent of 3,5-dimethoxybenzyl alcohol

The reaction was monitored by ^{1}H NMR and after 3 h, full conversion was observed. Then, the reaction mixture was concentrated and purified via flash chromatography (Pentane: Ethyl acetate: 80/20) to afford 93 mg of a colorless oil (96% Yield).

^{1}H NMR (300 MHz, $CDCl_3$) δ 6.42 (m, 2H), 6.41 – 6.37 (m, 1H), 5.38 (q, *J* = 1.5 Hz, 1H), 5.10 (bs, 2H), 3.78 (s, 6H), 3.48 (d, *J* = 18.2 Hz, 1H), 2.90 (d, *J* = 18.2 Hz, 1H), 2.16 (s, 3H), 2.05 (d, *J* = 1.5 Hz, 3H).
^{13}C NMR (75.5 MHz, $CDCl_3$) δ 202.7, 174.2, 164.2, 161.1, 154.2, 138.1, 105.2, 105.6, 100.5, 67.7, 56.9, 55.5, 46.5, 30.0, 14.1.
HRMS: (DCI, CH_4) calculated for $C_{18}H_{20}O_7$: 348.1209; found: 348.1210.

bis(3,5-dimethoxybenzyl) 2,2-bis(2-oxopropyl)malonate (10)

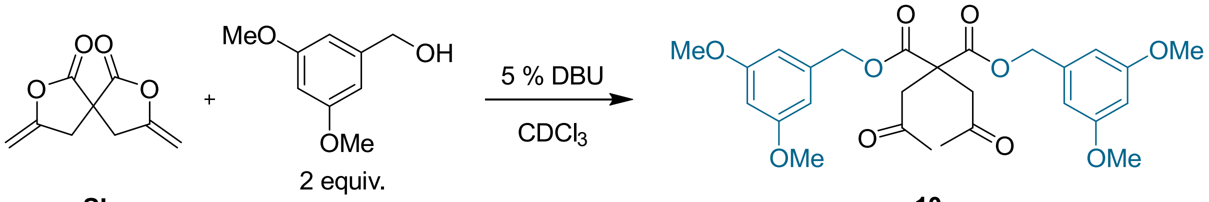

γSL

10

Scheme S5. Reaction of lactone **γSL** with 2 equivalent of 3,5-dimethoxybenzyl alcohol

The reaction was monitored by ^{1}H NMR and after 2 h, full conversion was observed. Then, the reaction mixture was concentrated and purified via flash chromatography (Pentane: Ethyl acetate: 80/20) to afford 143 mg of a colorless oil (99% Yield).

10 could also be obtained by addition of one equivalent of 3,5-dimethoxybenzyl alcohol to **9** in the presence of 2.5% DBU.

^{1}H NMR (300 MHz, $CDCl_3$) δ δ 6.44 – 6.30 (m, 6H), 5.05 (s, 4H), 3.75 (s, 12H), 3.41 (s, 4H), 2.09 (s, 6H).

^{13}C NMR (75.5 MHz, $CDCl_3$) δ 206.0, 169.3, 161.0, 137.7, 105.7, 100.3, 67.6, 55.4, 53.3, 16.0, 30.3.

HRMS: (DCI, CH_4) calculated for $C_{27}H_{32}O_{10}$: 516.1995; found: 516.2000.

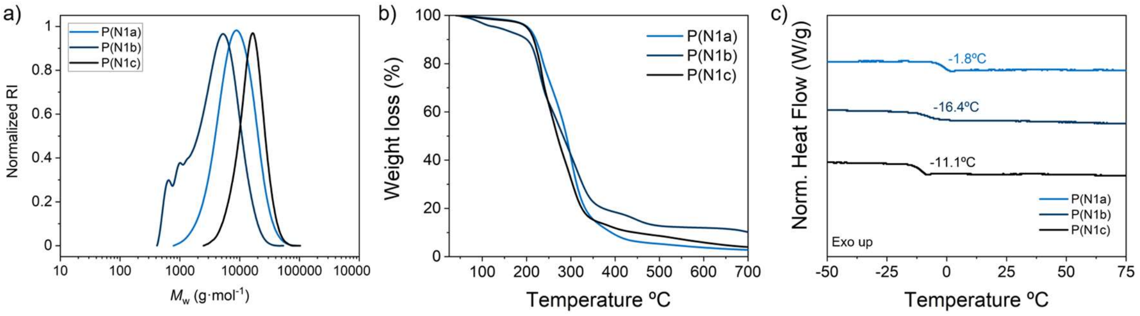

Figure S13. a) SEC traces b) TGA measurements and c) DSC curves for representative P(N1a-c) samples (Table S1, entries 6, 11 and 15, respectively).

3.5 Model reactions with 1 or γSL and monofunctional thiols and amines

General prodedure for the reactions with benzyl mercaptan in $CDCl_3$. In a glovebox, **1** or **γSL** (1 equiv.) and Benzyl mercaptan (1 or 2 equiv.) were added in a schlenk tube. The reactants were solubilized anhydrous $CDCl_3$ ([C] = 0.2 M) and a DBU solution (2.5 or 5 %mol) in $CDCl_3$ (C = 0.063 M) was added to the reaction medium and the reaction was stirred at 25 °C.

*Model lactone **1** with benzyl mercaptan.* In the absence of DBU (0.2 M in $CDCl_3$, 2h), no reaction occurred. However, under the same reaction conditions and the addition of 2.5 % of DBU, full and clean conversion of the lactone in the corresponding β-oxo-thioester **11** was observed in less than 25 minutes (as demonstrated by the disappearance on the

^{1}H NMR spectrum of the signal corresponding to the C=C moiety at 4.38 and 4.82 ppm and appearance of a new *s* signal at 2.13 ppm attributed to the methyl ketone group). The product could be isolated and characterized, but when the reaction was allowed to continue, slow evolution of this kinetic product could be observed over 48 h towards the β-thioether lactone **12** as a mixture of diastereomers (disappearance of the signal corresponding to the methyl ketone group at 2.13 ppm and the apparition of a new *s* signal at 1.85/1.80 ppm attributed to the methyl group). Indeed, it is the thermodynamic product, resulting from the nucleophilic addition of the thiol to the conjugated C=C moiety rather than to the ester group.[7]

methyl 2-((benzylthio)carbonyl)-2-methyl-4-oxopentanoate (11)

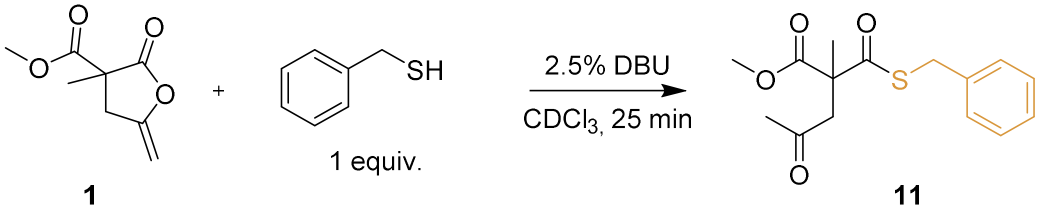

Scheme S6. Reaction of lactone **1** with benzyl mercaptan

The reaction was monitored by ^{1}H NMR and after 25 min, full conversion was observed. Then, the reaction mixture was concentrated and purified via flash chromatography (Pentane: Ethyl acetate: 80/20) to afford 79 mg of a colorless oil (91% Yield).

^{1}H NMR (300 MHz, $CDCl_3$) δ 7.37 – 7.10 (m, 5H), 4.11 (s, 2H), 3.69 (s, 3H), 3.26 (d, *J* = 18.2 Hz, 1H), 3.03 (d, *J* = 18.2 Hz, 1H), 2.13 (s, 3H), 1.58 (s, 3H).

^{13}C NMR (75.5 MHz, $CDCl_3$) δ 204.7, 171.3, 136.8, 129.0, 128.7, 127.5, 58.6, 53.0, 49.0, 33.7, 30.4, 20.8.

methyl 5-(benzylthio)-3,5-dimethyl-2-oxotetrahydrofuran-3-carboxylate (12)

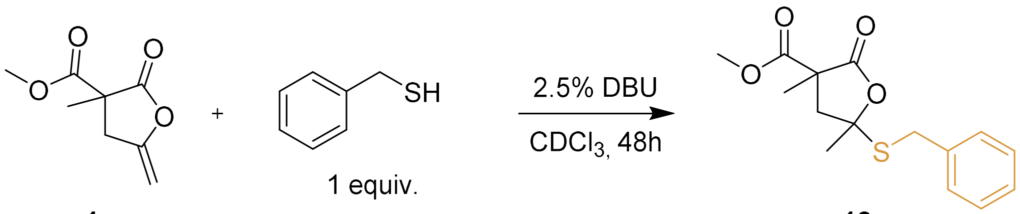

Scheme S7. Reaction of lactone **1** with benzyl mercaptan for 48h

The reaction was monitored by ^{1}H NMR and after 48 h, full conversion was observed. Then, the reaction mixture was concentrated and purified via flash chromatography (Pentane: Ethyl acetate: 80/20) to afford 85 mg of a colorless oil (90% Yield).

Major dias

[7] F. Ouhib, B. Grignard, E. Van Den Broeck, A. Luxen, K. Robeyns, V. Van Speybroeck, C. Jerome, C. Detrembleur, *Angew. Chem. Int. Ed.* **2019**, *58*, 11768–11773.

¹H NMR (300 MHz, CDCl₃) δ 7.46 – 7.09 (m, 5H), 4.00 (s, 2H), 3.79 (s, 3H), 2.94 (d, J = 14.2 Hz, 1H), 2.26 (d, J = 14.3 Hz, 1H), 1.85 (s, 3H), 1.66 (s, 3H).
¹³C NMR (75.5 MHz, CDCl₃) δ 174.3, 171.2, 136.5, 129.1, 128.7, 127.4, 90.1, 53.4, 52.1, 47.4, 34.2, 29.3, 21.8.

Minor dias

¹H NMR (300 MHz, CDCl₃) δ 7.46 – 7.09 (m, 5H), 3.94 (s, 1H), 3.93 (s, 1H), 3.72 (s, 3H), 3.07 (d, *J* = 15 Hz, 1H), 2.20 (d, *J* = 15 Hz, 1H), 1.80 (s, 3H), 1.58 (s, 3H).
¹³C NMR (75.5 MHz, CDCl₃) δ 174.1, 171.6, 136.9, 128.9, 128.6, 127.3, 89.5, 53.3, 52.4, 48.0, 33.8, 29.5, 22.5.

HRMS: (DCI, CH_4) calculated for $C_{15}H_{19}O_4S$: 295.1004; found: 295.0999.

3,8-bis(benzylthio)-3,8-dimethyl-2,7-dioxaspiro[4.4]nonane-1,6-dione (13)

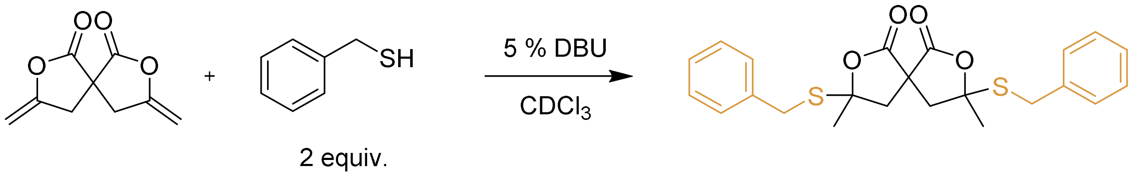

γSL 13

Scheme S8. Reaction of lactone **γSL** with 2 equivalents of benzyl mercaptan

The reaction was monitored by ¹H NMR and after 4 h, full conversion was observed. Then, the reaction mixture was concentrated and purified via flash chromatography (Pentane: Ethyl acetate: 80/20) to afford 115 mg of a colorless oil (97% Yield).

¹H NMR (300 MHz, CDCl₃) δ 7.36 – 7.20 (m, 12H), 3.97 (d, *J* = 2.5 Hz, 4H), 2.96 (d, *J* = 14.5 Hz, 2H), 2.56 (d, *J* = 14.5 Hz, 2H), 1.92 (s, 6H).
¹³C NMR (75.5 MHz, CDCl₃) δ 173.5, 136.5, 129.1, 128.9, 127.6, 91.8, 55.3, 48.4, 34.1, 28.8.
HRMS: (DCI, CH_4) calculated for $C_{23}H_{25}O_4S_2$: 429.1194; found: 429.1189.

General prodedure for the reactions with Benzylamine in CDCl₃. In a glovebox, **1** or **γSL** (1 equiv.) and Benzylamine (1 or 2 equiv.) were added in a schlenk tube. The reactants were solubilized anhydrous $CDCl_3$ ([C] = 0.2 M) and the reaction was stirred at 25 °C.

*Model lactone **1** with benzylamine* does not require the presence of DBU, although the reaction is rather slow at rt (full conversion is observed after 24h in 0.2 M $CDCl_3$). Clean formation of the corresponding β-oxo-amide was observed (supported by disappearance on the ¹H NMR spectrum of the signal corresponding to the C=C moiety at 4.38 and 4.82 ppm and the appearance of a new *s* signal at 2.17 ppm attributed to the methyl ketone group). The addition of DBU (2.5%) at this stage of the reaction, or passing the reaction media through a silica pad, led to the rapid formation of the β-hydroxy-lactame as a

mixture of diastereomers, resulting from the intramolecular nucleophilic addition of the secondary amide moiety of the β-oxo-amide to the ketone group (disappearance on the ^{1}H NMR spectrum of the signal corresponding to the methyl ketone group and apparition of a new s signal at 1.57/1.53 ppm attributed to the methyl groups).

methyl 2-(benzylcarbamoyl)-2-methyl-4-oxopentanoate (14)

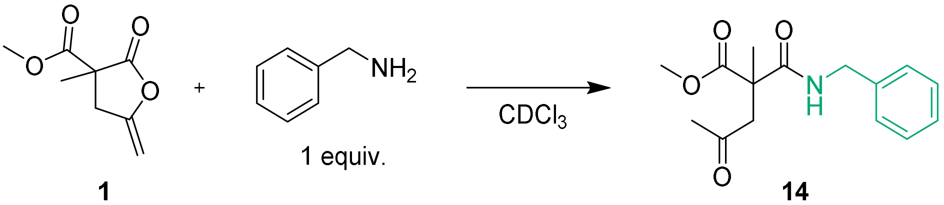

1 14

Scheme S9. Reaction of lactone **1** with benzyl amine

The reaction was monitored by ^{1}H NMR and after 24 h, full conversion was observed. Then, the reaction mixture was concentrated and the product was characterized without any purification (76 mg, 93% Yield).

^{1}H NMR (300 MHz, CDCl$_3$) δ 8.23 (bs, 1H), 7.40 – 7.16 (m, 5H), 4.55 – 4.43 (m, 2H), 3.70 (s, 3H), 3.45 (d, *J* = 18.3 Hz, 1H), 3.04 (d, *J* = 18.3 Hz, 1H), 2.17 (s, 3H), 1.51 (s, 3H).

^{13}C NMR (75.5 MHz, CDCl$_3$) δ 175.5, 171.2, 183.3, 128.6, 127.3, 127.2, 89.9, 52.7, 50.0, 49.8, 43.6, 29.6, 20.7.

HRMS: (ESI+) calculated for $C_{15}H_{20}NO_4$: 278.1392; found: 278.1400.

methyl 1-benzyl-5-hydroxy-3,5-dimethyl-2-oxopyrrolidine-3-carboxylate (15)

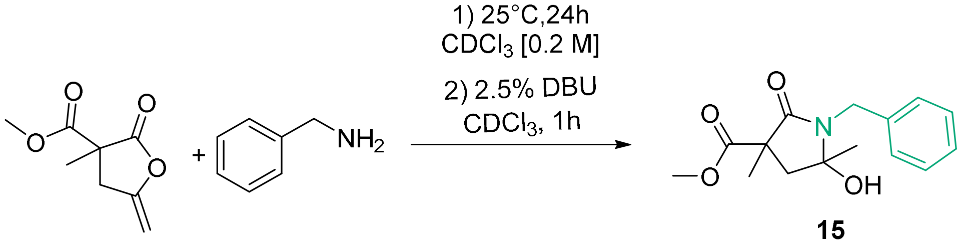

15

1

Scheme S10. Reaction of lactone **1** with benzyl amine and DBU

The lactone and benzylamine was charged in a schlenk tube and solubilized with $CDCl_3$ ([C] = 0.2 M). The reaction was monitored by ^{1}H NMR and after 24 h, full conversion to the opened product **14** was observed. Then, a solution of DBU in $CDCl_3$ (2.5%, [C] = 0.0063) was added and the full conversion to the hemiaminal product was monitored by ^{1}H NMR in 1h. The reaction mixture was concentrated and purified via flash chromatography (Pentane: Ethyl acetate: 80/20) to afford 79 mg of a colorless oil (97% Yield).

<u>Major dias</u>

^{1}H NMR (300 MHz, CDCl$_3$) δ 7.32-7.18 (m, 5H), 4.75 (bs, 1H), 4.72 (d, *J* = 15 Hz, 1H), 4.33 (d, *J* = 15 Hz, 1H), 3.79 (s, 3H), 2.59 (d, *J* = 15 Hz, 1H), 2.00 (d, *J* = 15 Hz, 1H), 1.53 (s, 3H), 1.35 (s, 3H).
^{13}C NMR (75.5 MHz, CDCl$_3$) δ 175.3, 172.7, 138.3, 128.5, 127.6, 127.2, 87.5, 53.7, 51.4, 47.2, 43.1, 26.9, 20.6.

Minor dias

^{1}H NMR (300 MHz, CDCl$_3$) δ 7.32-7.18 (m, 5H), 4. (bs, 1H), 4.63 (d, *J* = 15 Hz, 1H), 4.40 (d, *J* = 15 Hz, 1H), 3.75 (s, 3H), 2.67 (d, *J* = 15 Hz, 1H), 2.06 (d, *J* = 15 Hz, 1H), 1.57 (s, 3H), 1.40 (s, 3H).
^{13}C NMR (75.5 MHz, CDCl$_3$) δ 173.5, 173.2, 138.2, 128.6, 127.5, 127.2, 88.4, 52.8, 51.2, 47.4, 42.6, 27.6, 21.6.
HRMS: (ESI+) calculated for $C_{15}H_{20}NO_4$: 278.1392; found: 278.1379.

2,7-dibenzyl-3,8-dihydroxy-3,8-dimethyl-2,7-diazaspiro[4.4]nonane-1,6-dione (16)

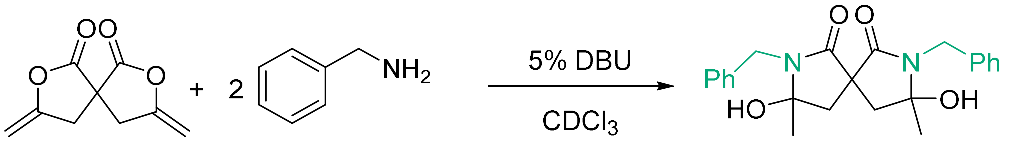

γSL　　　16

Scheme S11. Reaction of lactone **γSL** with 2 equivalents of benzyl amine and DBU

The reaction was monitored by ^{1}H NMR and after 5 min, full conversion was observed. Then, the reaction mixture was concentrated and purified via flash chromatography (Pentane: Ethyl acetate: 80/20) to afford 104 mg of a colorless oil (95% Yield).

^{1}H NMR (300 MHz, CDCl$_3$) δ 7.49 – 7.08 (m, 10H), 6.15 (s, 2H), 4.77 (d, *J* = 15.5 Hz, 2H), 4.39 (d, *J* = 15.5 Hz, 2H), 2.56 (d, *J* = 13.6 Hz, 2H), 2.19 (d, *J* = 13.5 Hz, 2H), 1.47 (s, 6H).
^{13}C NMR (75.5 MHz, CDCl$_3$) δ 174.7, 137.7, 128.7, 127.6, 127.5, 89.1, 54.1, 43.5, 43.0, 26.2
HRMS: (DCI, CH$_4$) calculated for $C_{23}H_{25}N_2O_3$ (MH^+-H_2O) : 377.1865; found: 377.1857.

3.6 Synthesis and characterization of the poly(spiro-bis(β-thioether lactone))s and poly(spiro-bis (β-hydroxy-lactame))s.

General procedure for the preparation of the poly(β-oxo-thioester)s and poly(spiro-bis(β-thioether lactone))s.

40 mg (0.22 mmol, 1 eq.) of **γSL** and the corresponding dithiol were added to a glass vial. Next, 50 μL of a DBU solution (36.6 mg/mL, DMF) was added, and DMF was added until a final volume of 0.11 mL (2.0 M, 5% DBU) of DMF. After stirring for 24 h at 25 °C, an aliquot was taken out for conversion calculation and then precipitated in H_2O. The polymers were precipitated two times, isolated by centrifugation and dried under a vacuum.

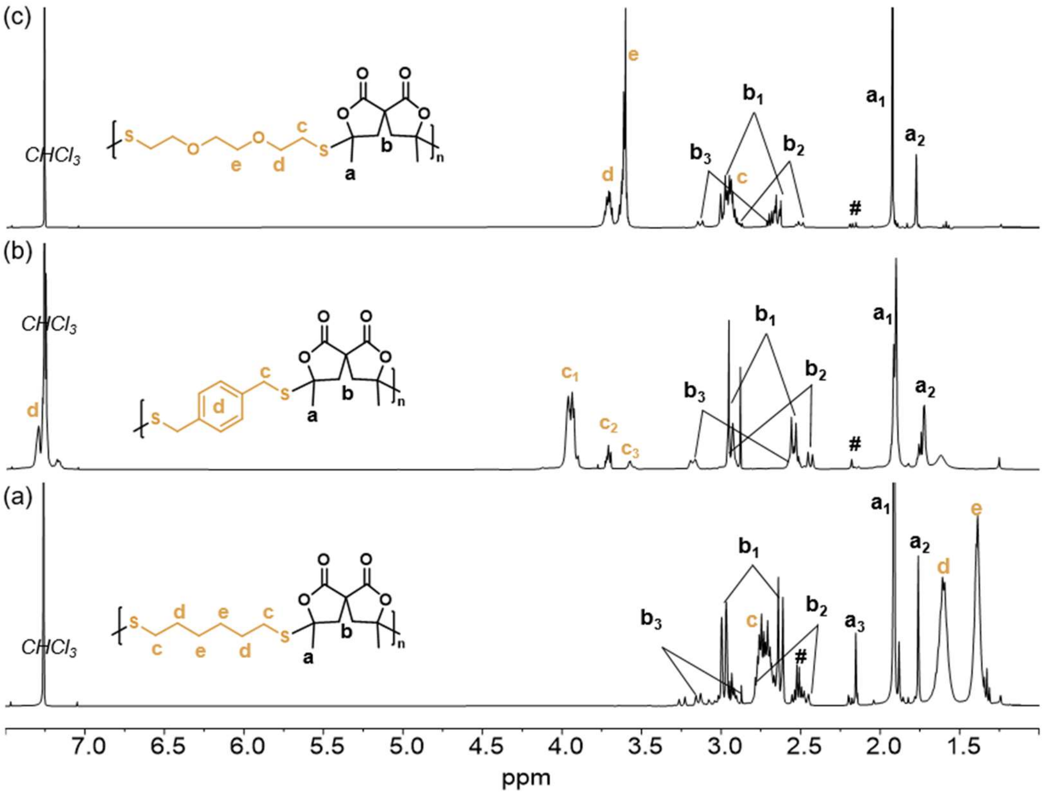

Figure S14. Stacked ^{1}H NMR spectra of P(N2a-c) in $CDCl_3$, purified by precipitation. # corresponds to the kinetic product, thioester structure.

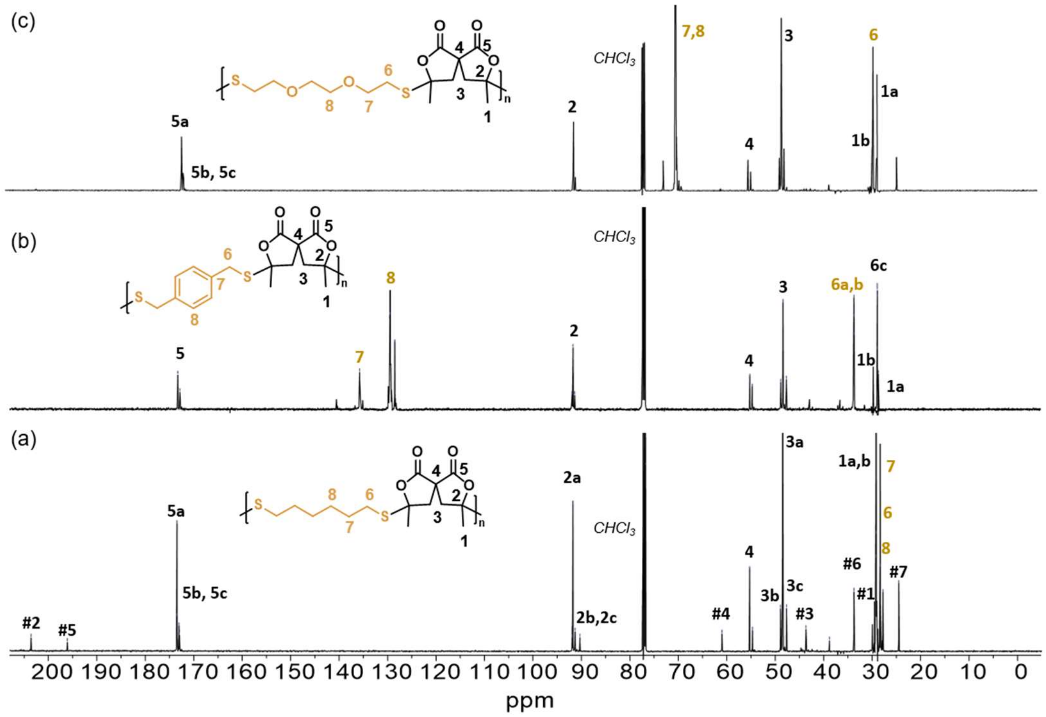

Figure S15. Stacked ^{13}C NMR spectra of P(N2a-c) in $CDCl_3$, purified by precipitation. Different stereoisomers are indicated as a,b,c and # corresponds to the kinetic product, thioester structure.

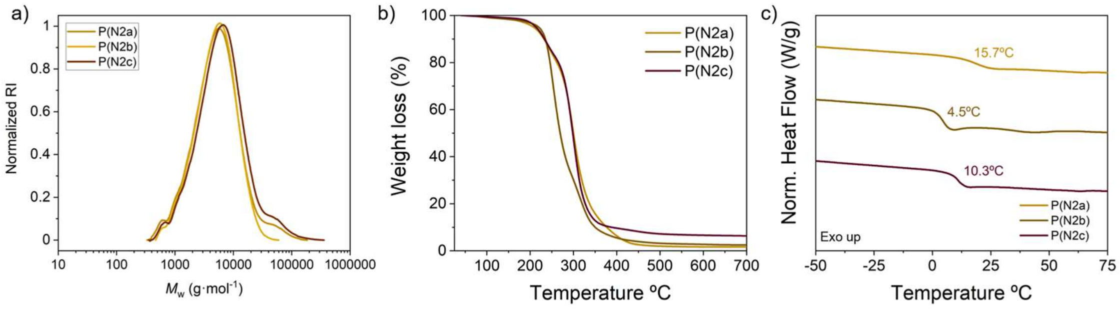

Figure S16. a) SEC traces b) TGA measurements and c) DSC curves of P(N2a-c) (Table 2).

<u>General procedure for the preparation of the poly(β-oxo-amide) and poly(spiro-bis(β-hydroxy-lactame)).</u>

40 mg (0.22 mmol, 1 eq.) of **γSL** and the corresponding diamine were added to a glass vial. Next, 50 µL of a DBU solution (36.6 mg/mL, DMF) were added, and DMF was added until a final volume of 0.11 mL (2.0 M, 5% DBU) of DMF. After stirring for 24 h at 25 °C, an aliquot was taken out for conversion calculation and then precipitated in H_2O. The polymers were precipitated two times, isolated by centrifugation and dried under a vacuum.

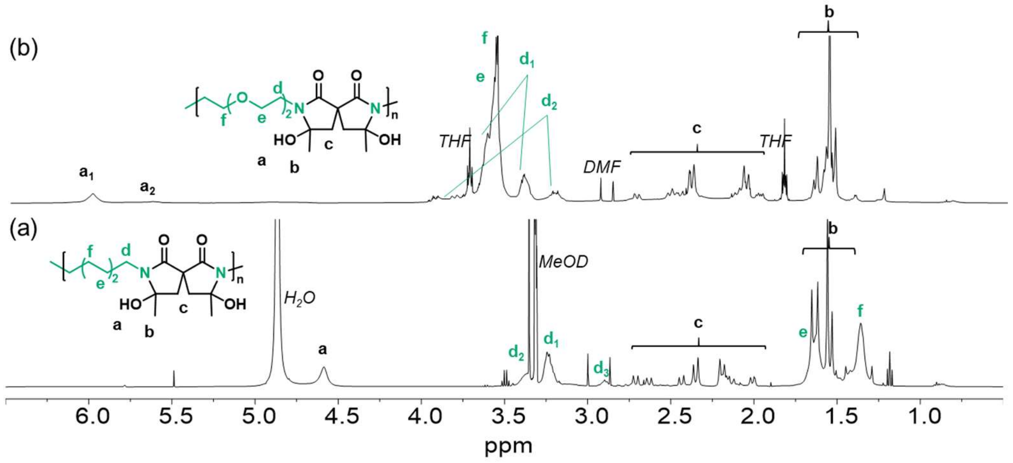

Figure S17. Stacked ^{1}H NMR spectra of P(N3a,b) in CD_3OD (P(N3a)) or $CDCl_3$ (P(N3b)), purified by precipitation.

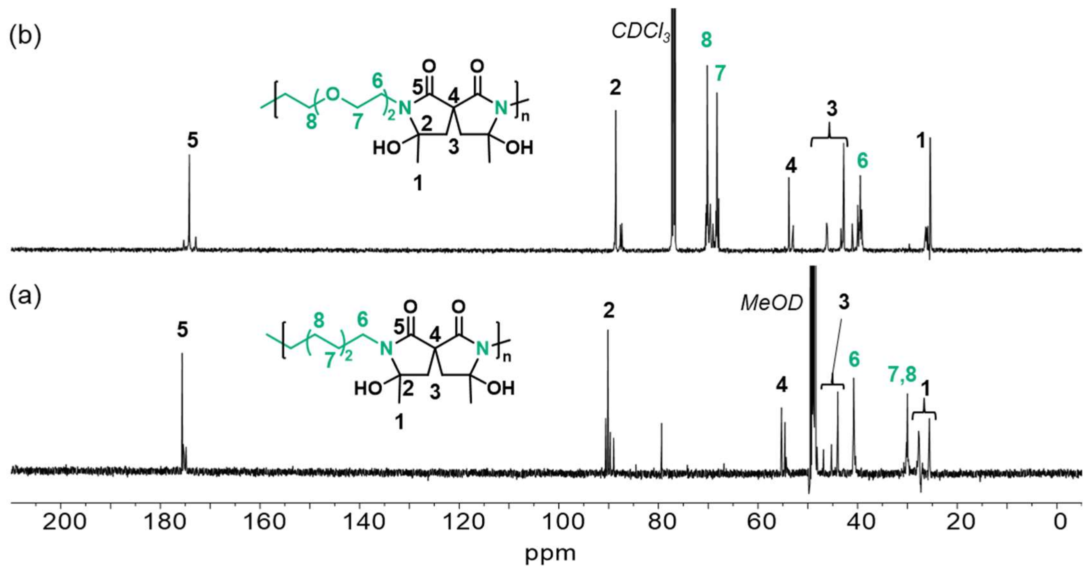

Figure S18. Stacked ^{13}CNMR spectra of P(N3a,b) in $CDCl_3$, purified by precipitation.

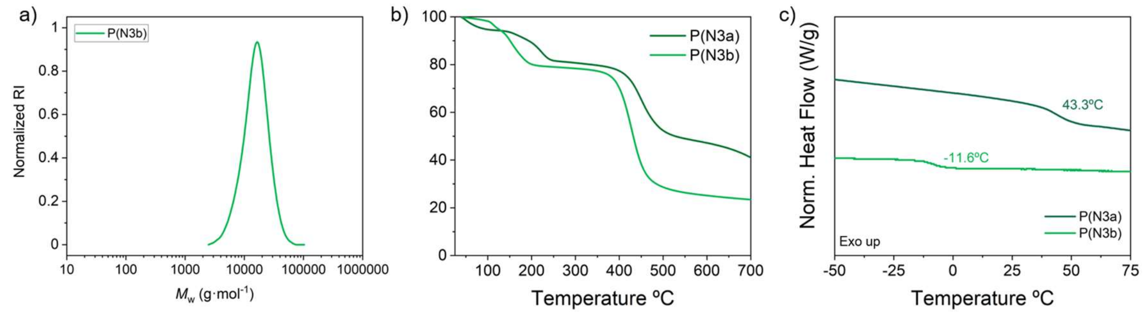

Figure S19. a) SEC trace of P(N3b) b) TGA measurements of P(N3a,b) and c) DSC curves of P(N3a,b) (Table 2, samples prepared without DBU).

Table S2. TGA analysis of the polymers prepared.

Entry	Polymer	T_d10% (ºC)
1	P(N1a)	220
2	P(N1b)	200
3	P(N1c)	216
4	P(N2a)	232
5	P(N2b)	235
6	P(N2c)	233
7	P(N3a)	192
8	P(N3b)	150

3.7 Synthesis and characterization of an $(ABAC)_n$ terpolymer.

1,4-phenylene*bis*(methylene)*bis*(5-methyl-2-oxo-3-(2-oxopropyl)-2,3-dihydrofuran-3-carboxylate) **17**

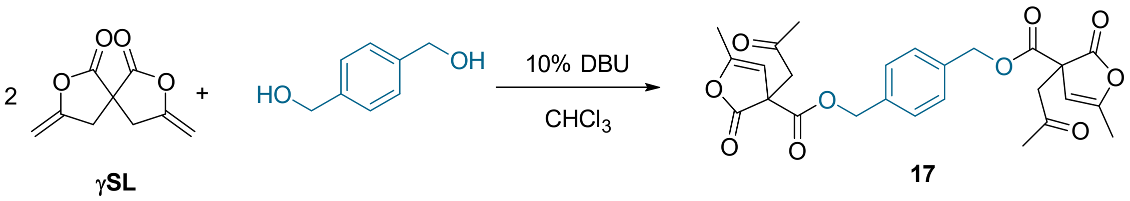

Scheme S12. Reaction of lactone **γSL** with 0.5 equivalent **N1a**

A dry schlenk was charged with 300 mg (1.66 mmol, 2 equiv.) of **γSL**, 115 mg (0.83 mmol, 1 equiv.) of 1,4-benzenedimethanol and 8.5 ml of chloroform under argon. Then, 25 µL of DBU was added (0.16 mmol). After stirring overnight at 25 °C, ^{1}H NMR control of th reaction media showed full conversion of **γSL** and the formation of **17** as the major product (> 90 % NMR yield). The reaction mixture was evaporated and purified by flash chromatography (eluent: Pentane/AcOEt: 50:50 to 30:70) to afford **17** as a colorless oil (108 mg, 26% yield).

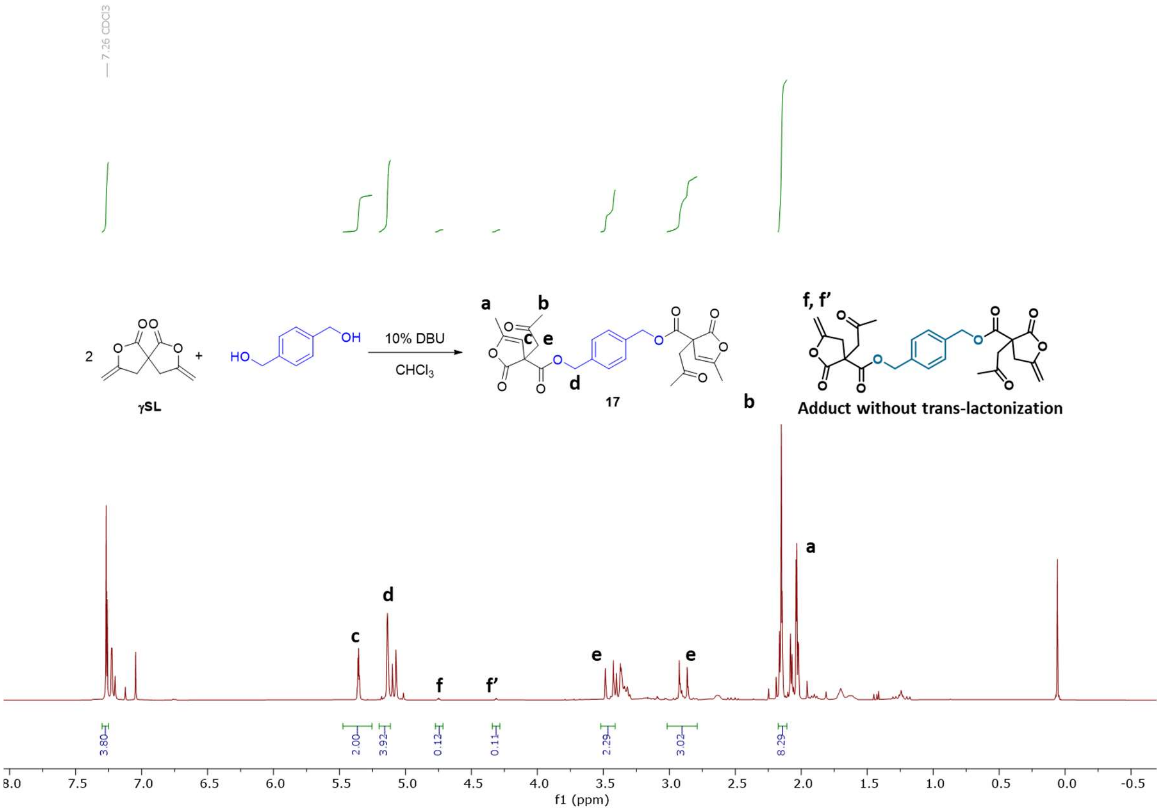

Figure S20. ^{1}H NMR spectrum of the reaction crude of the reaction of **γSL** with 0.5 equiv. of 1,4-benzenedimethanol.

^{1}H NMR (300 MHz, $CDCl_3$) δ δ 7.27 (s, 4H), 5.36 (q, *J*= 1.5Hz, 2H), 5.14 (m, 4H), 3.46

(d, J= 18.0 Hz, 4H), 2.90 (d, J= 18.0 Hz, 2H), 2.16 (s, 6H), 2.04 (d, J= 1.5 Hz, 6H).
^{13}C NMR (75.5 MHz, $CDCl_3$) δ 203.7, 174.1, 167.3, 155.3, 153.3, 135.3, 128.1, 104.5, 67.6, 56.9, 47.1, 38.9, 30.4, 30.0, 19.1, 14.1.

Terpolymerisation procedure:

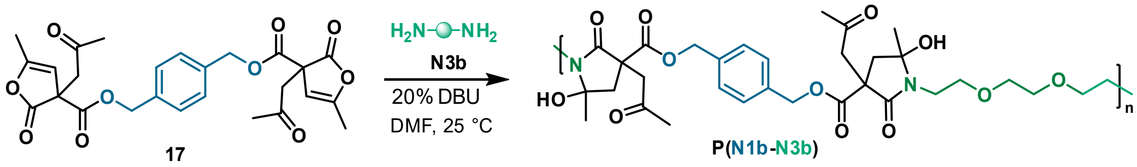

60 mg (0.12 mmol, 1 eq.) of **17** and 2,2'-(Ethylenedioxy)bis(ethylamine) (17.6 µl, 0.12 mmol) were added to a glass vial. Next, 3.6 µL DBU (0.023 mmol) were added, followed by 60 µL of DMF. After stirring for 24 h at 25 °C, the reaction mixture was precipitated in H_2O and 44 mg of polymer was isolated by centrifugation and dried under a vacuum (M_W = 17 850 g.mol^{-1} and $Đ$ = 2.66, SEC in DMF/10 mM LiBr, PMMA calibration).

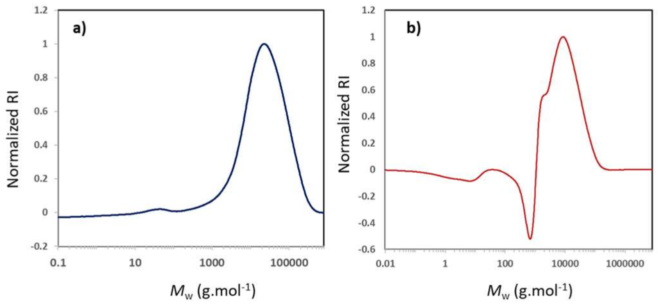

Figure S21. SEC traces (UVdetection (a) and RI detection (b)) for the termpolymer .

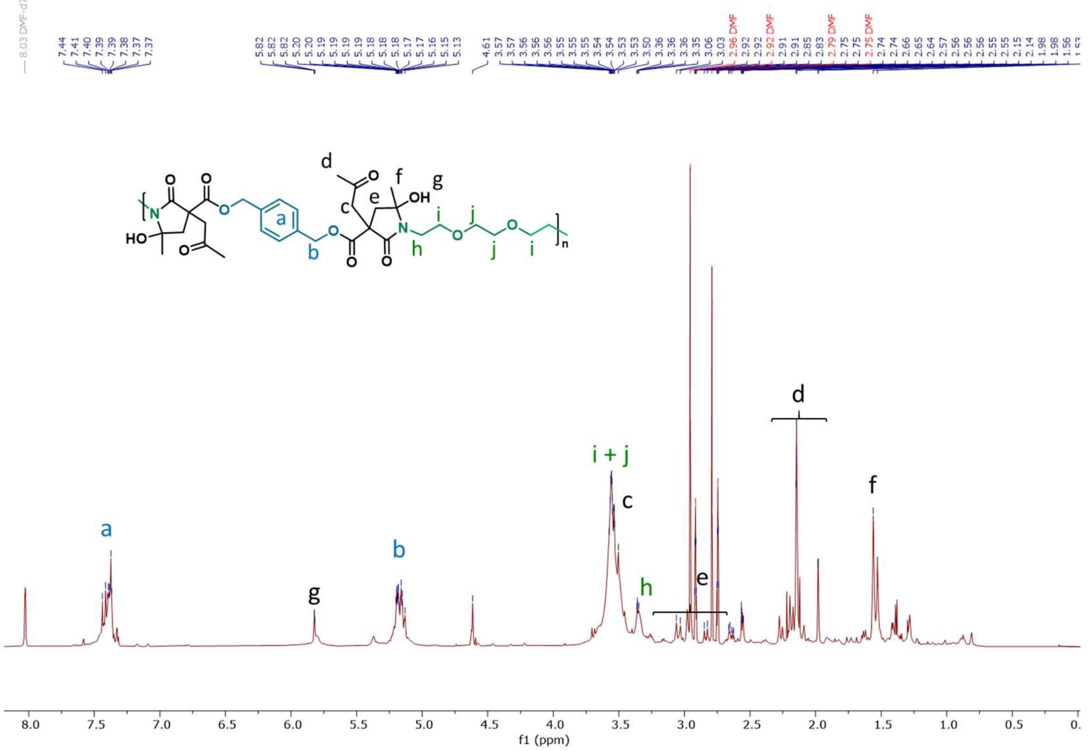

Figure S22.^{1}H NMR spectrum (600 MHz, DMF-d_7) of theterpolymer.

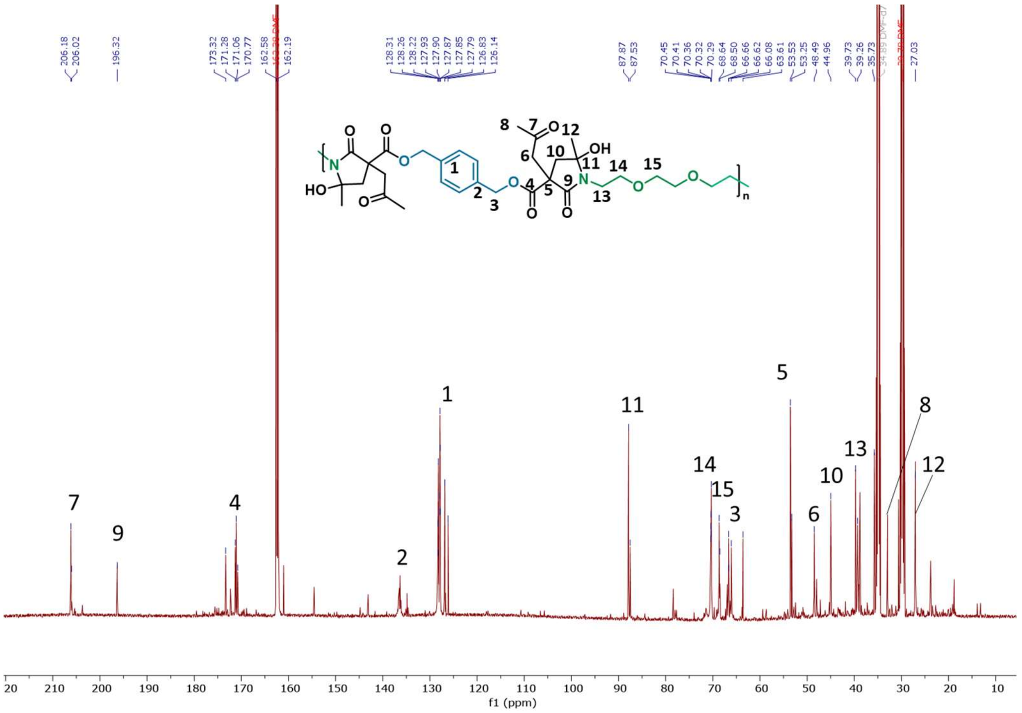

Figure S23. ^{13}C NMR spectrum (151 MHz, DMF-d_7) of the terpolymer.

4 NMR spectra of the molecular compounds

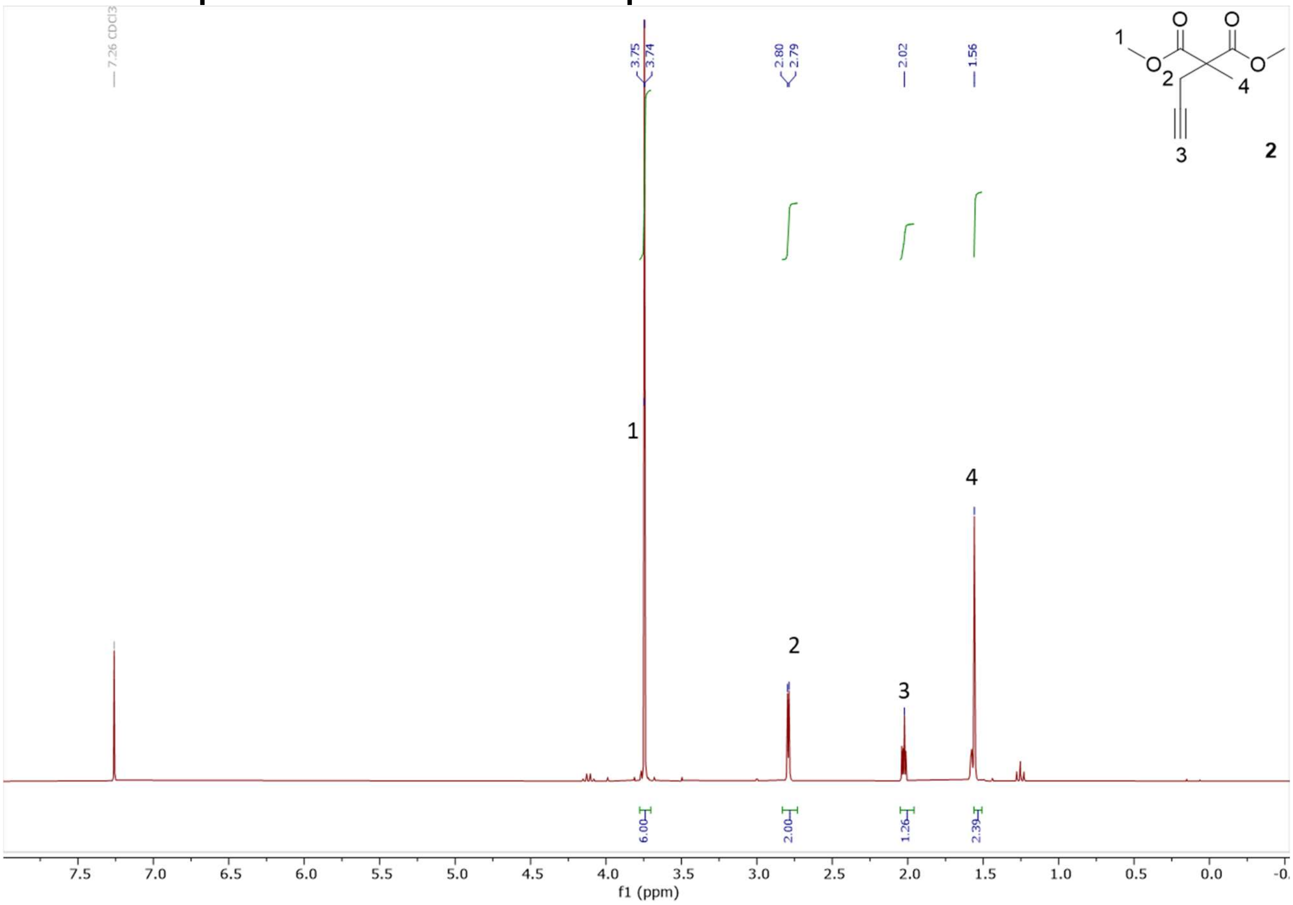

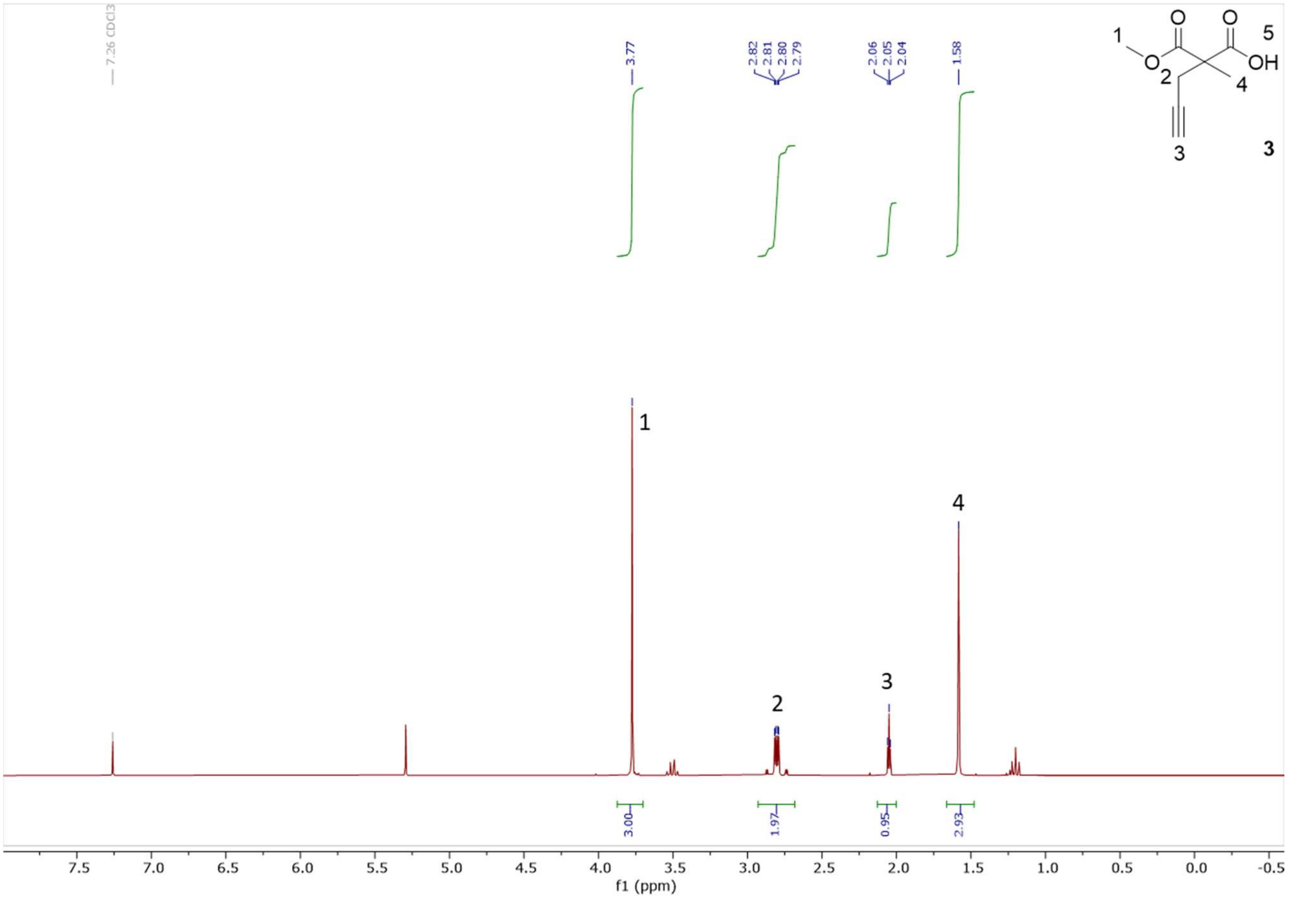

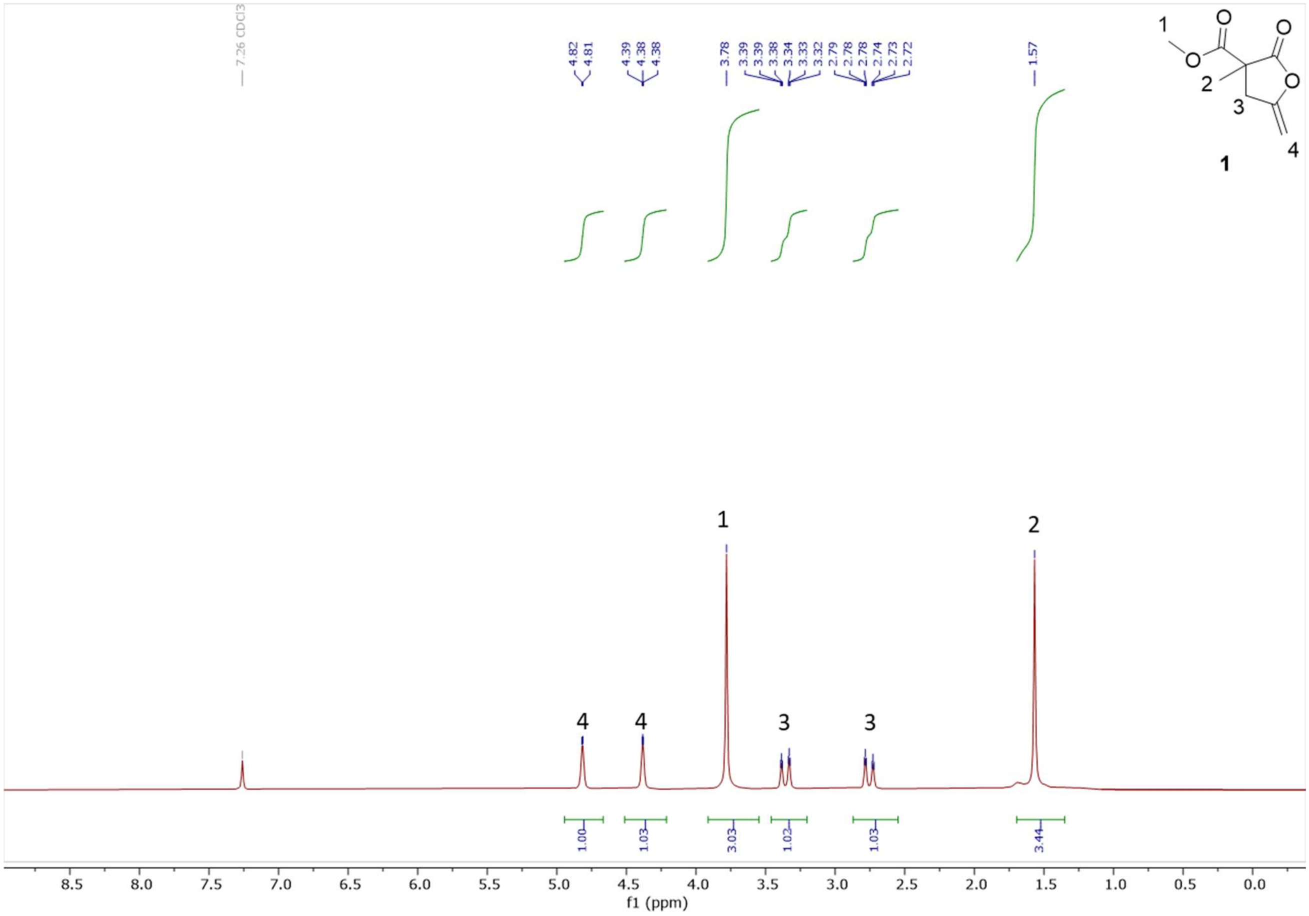
7.26 CDCl3
4.82
4.81
4.39
4.38
4.38
3.78
3.39
3.39
3.38
3.34
3.33
3.32
2.79
2.78
2.78
2.74
2.73
2.72
1.57
1
2
3
4
1
4
4
1
3
3
2
1.00
1.03
3.03
1.02
1.03
3.44
f1 (ppm)

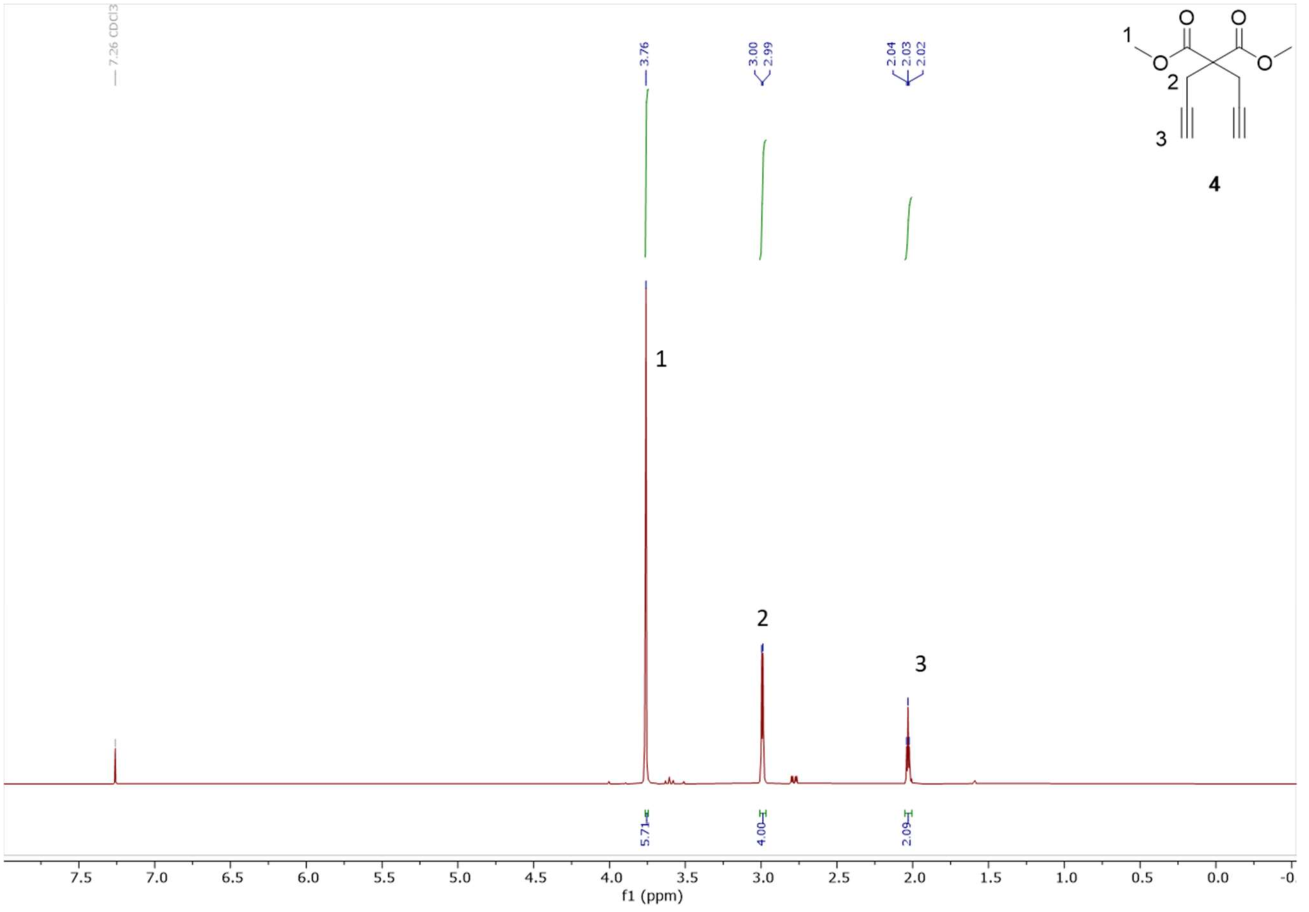
7.26 CDCl3
3.76
3.00
2.99
2.04
2.03
2.02
1
2
3
4
1
2
3
5.71
4.00
2.09
f1 (ppm)

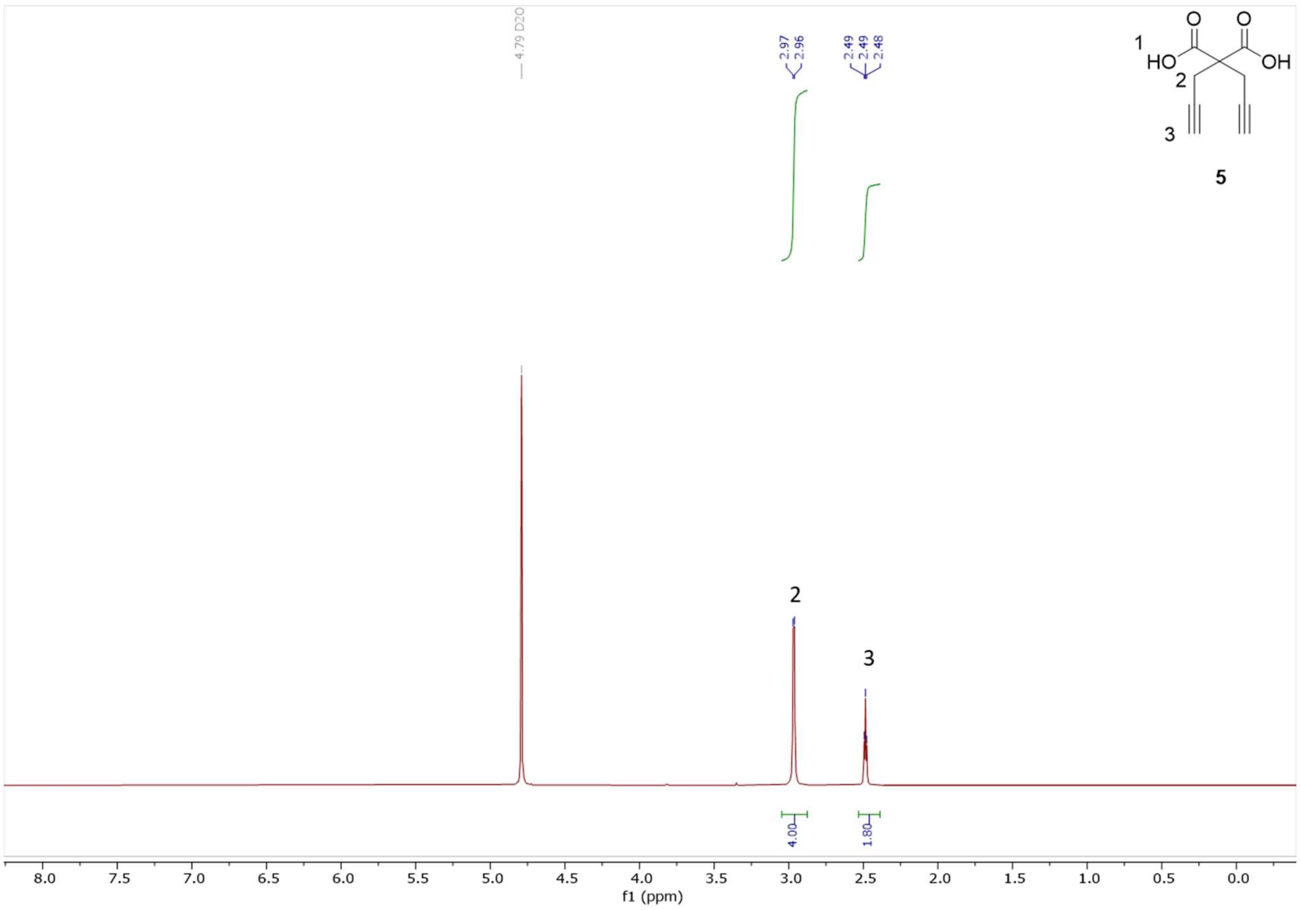
4.79 D2O
2.97
2.96
2.49
2.49
2.48
HO
OH
1
2
3
5
4.00
1.80
8.0 7.5 7.0 6.5 6.0 5.5 5.0 4.5 4.0 3.5 3.0 2.5 2.0 1.5 1.0 0.5 0.0
f1 (ppm)

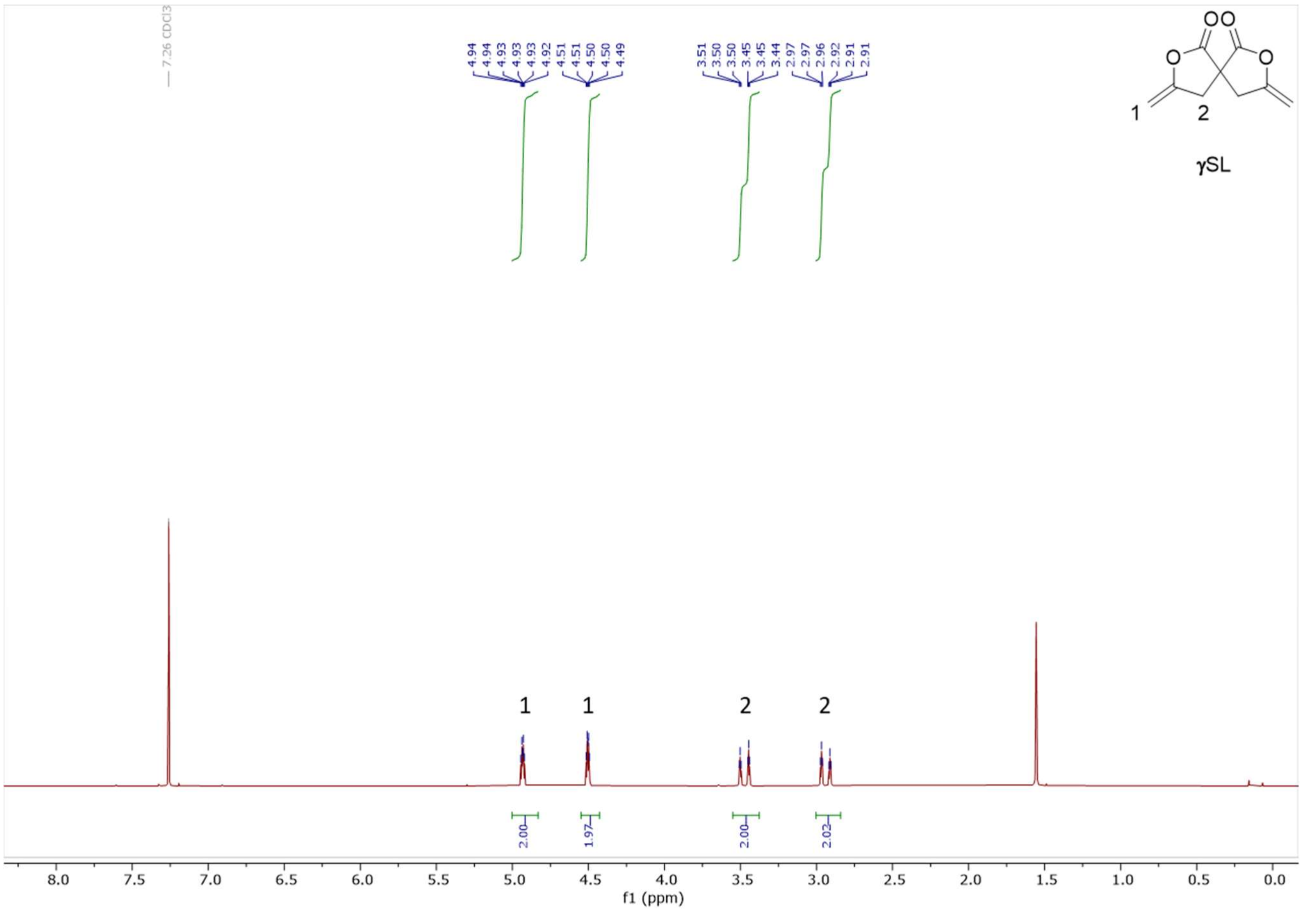
7.26 CDCl3
4.94
4.94
4.93
4.93
4.93
4.92
4.51
4.51
4.50
4.50
4.49
3.51
3.50
3.50
3.45
3.45
3.44
2.97
2.97
2.96
2.92
2.91
2.91
1
2
γSL
2.00
1.97
2.00
2.03
8.0 7.5 7.0 6.5 6.0 5.5 5.0 4.5 4.0 3.5 3.0 2.5 2.0 1.5 1.0 0.5 0.0
f1 (ppm)

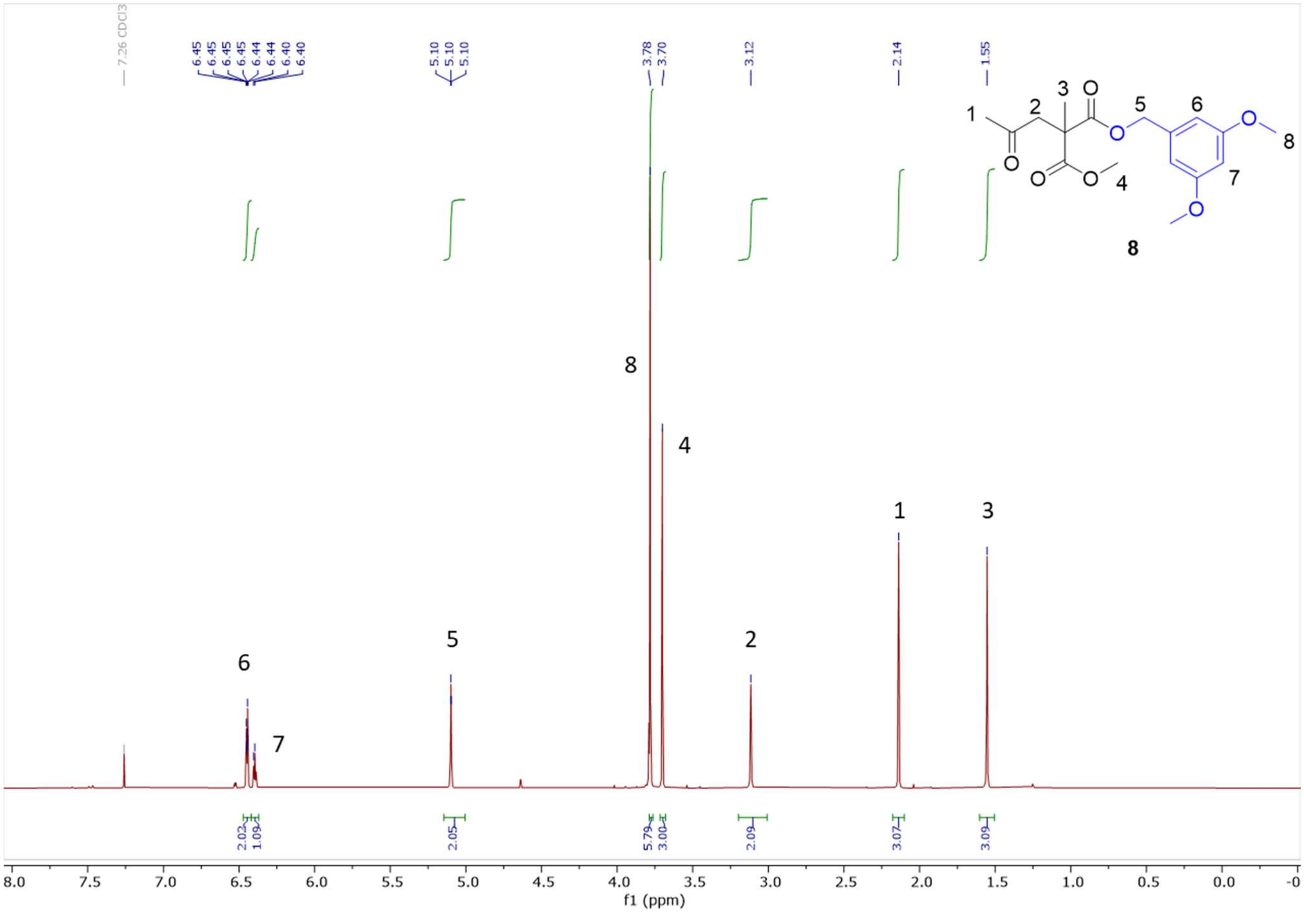
7.26 CDCl3
6.45
6.45
6.45
6.45
6.44
6.44
6.40
6.40
6.40
5.10
5.10
5.10
3.78
3.70
3.12
2.14
1.55
8
2.02
1.09
2.05
5.79
3.00
2.09
3.07
3.09
f1 (ppm)

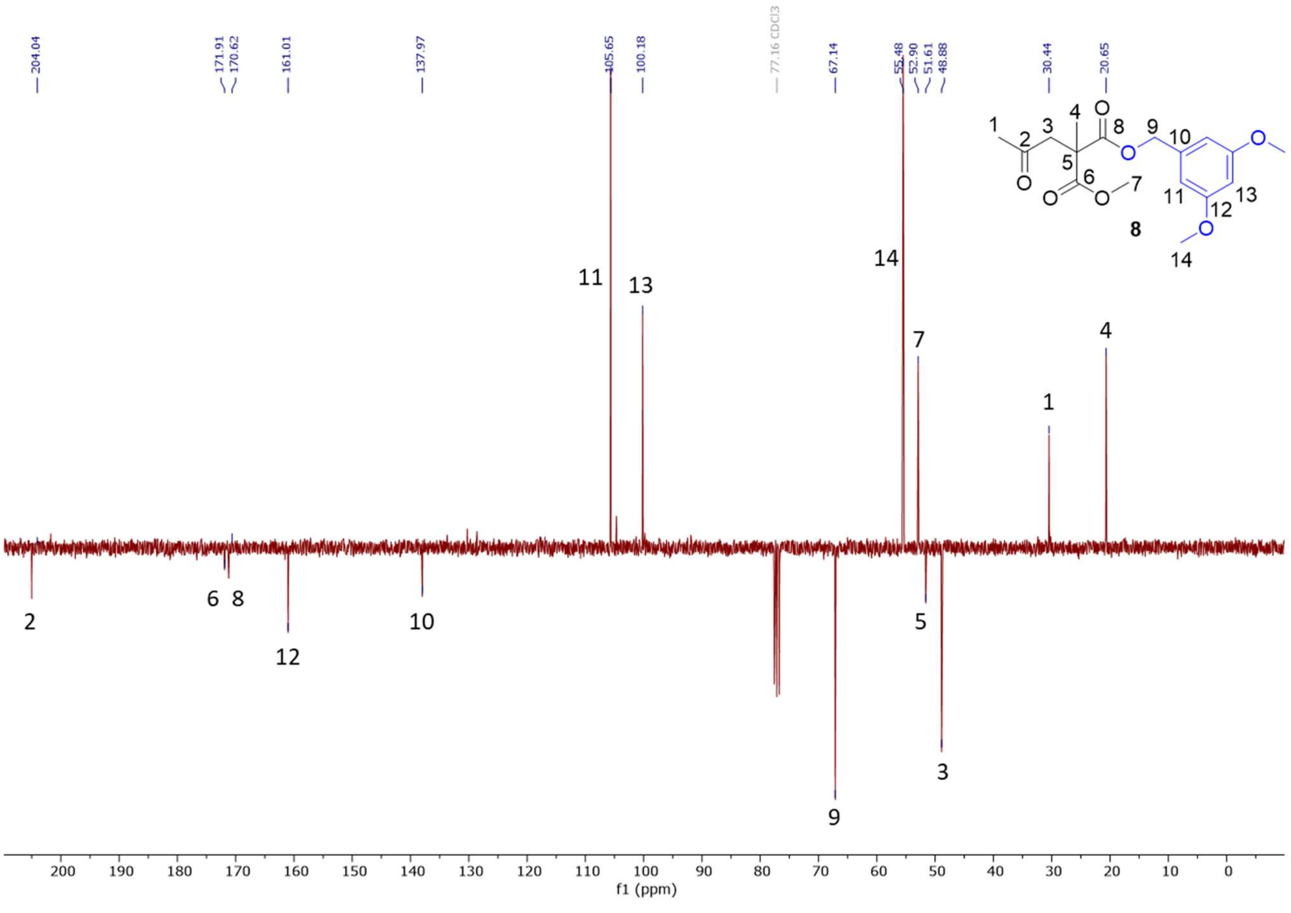
204.04
171.91
170.62
161.01
137.97
105.65
100.18
77.16 CDCl3
67.14
55.48
52.90
51.61
48.88
30.44
20.65
8
f1 (ppm)

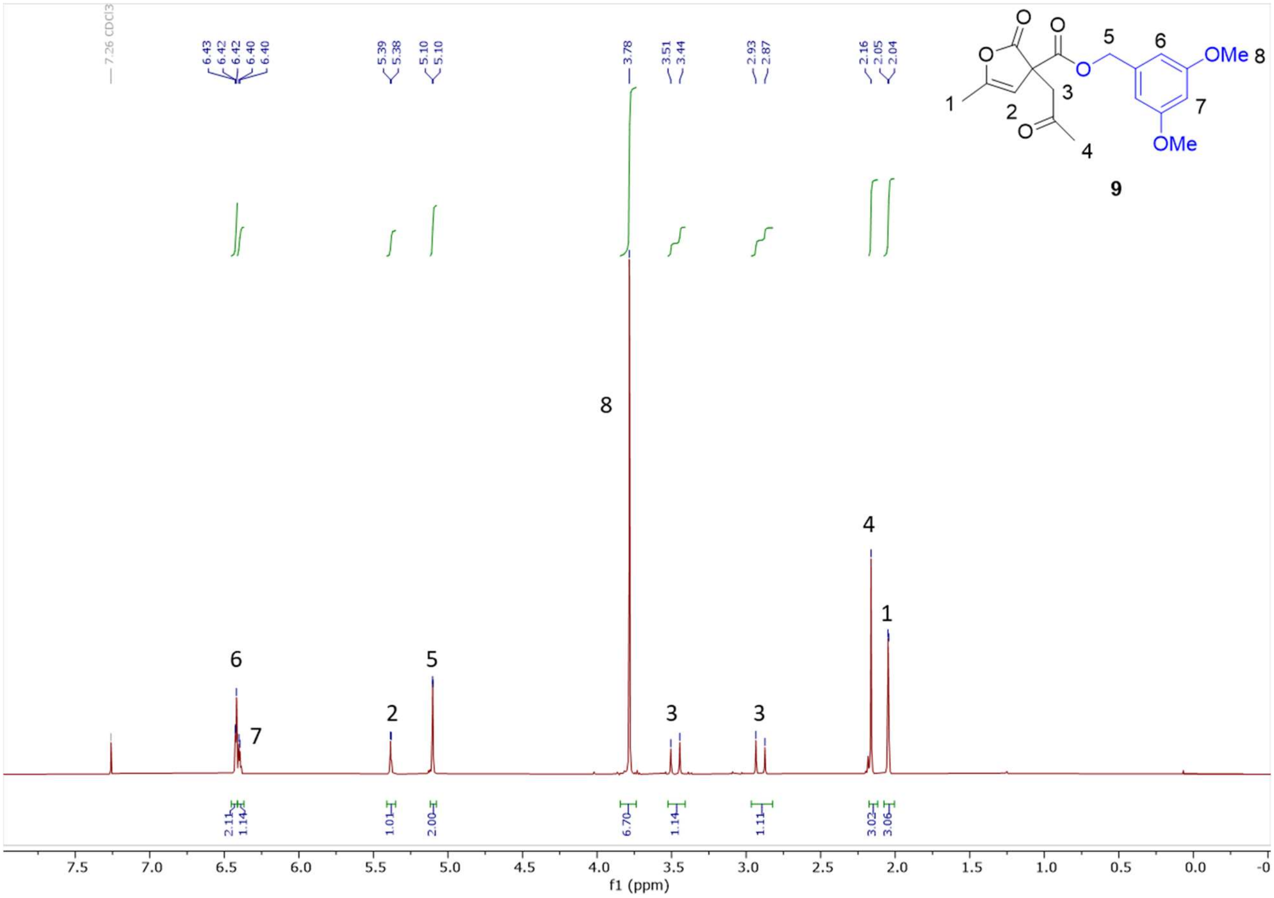
7.26 CDCl3
6.43
6.42
6.42
6.40
6.40
5.39
5.38
5.10
5.10
3.78
3.51
3.44
2.93
2.87
2.16
2.05
2.04
9
8
6
7
5
2
3
3
4
1
2.11
1.14
1.01
2.00
6.70
1.14
1.11
3.02
3.06
f1 (ppm)

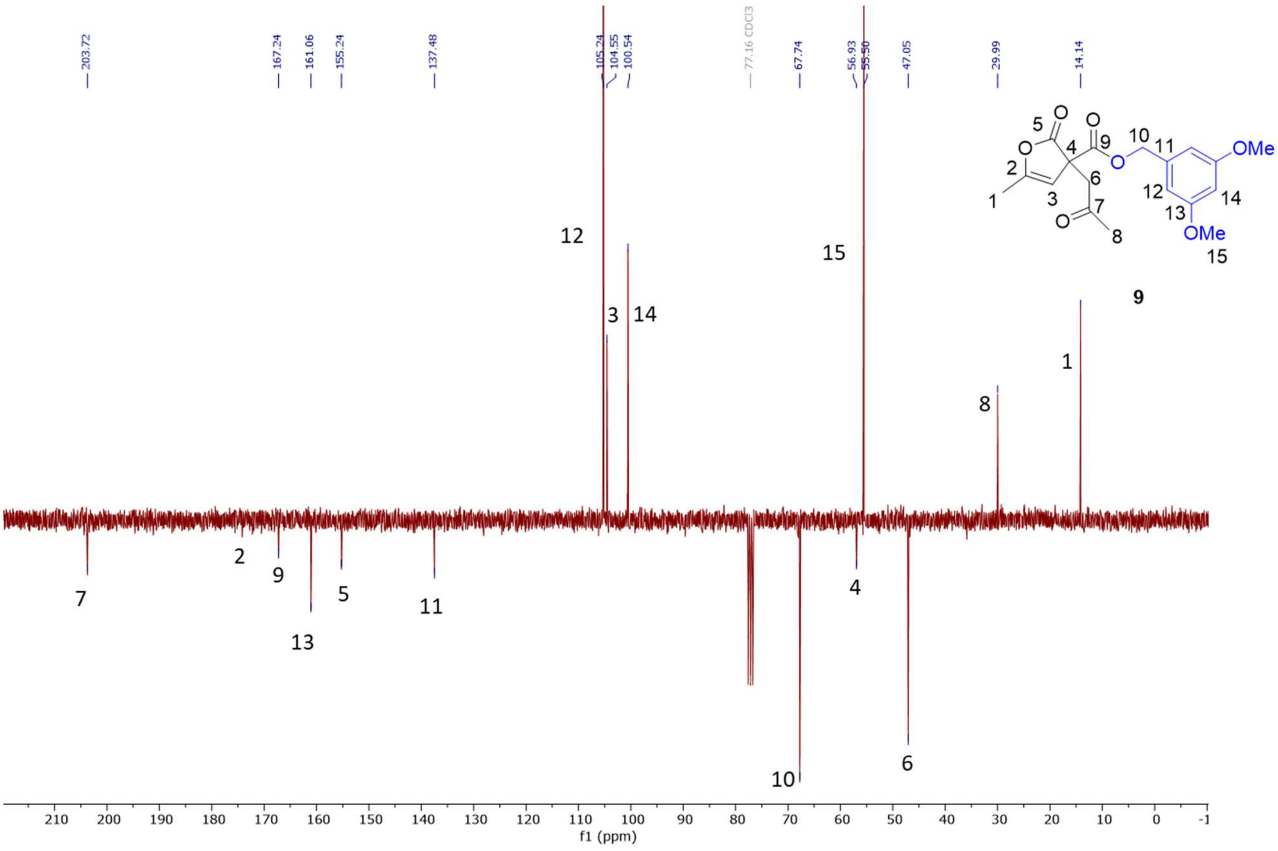
203.72
167.24
161.06
155.24
137.48
105.24
104.55
100.54
77.16 CDCl3
67.74
56.93
55.50
47.05
29.99
14.14
9
12
3
14
15
1
8
7
2
9
13
5
11
4
10
6
f1 (ppm)

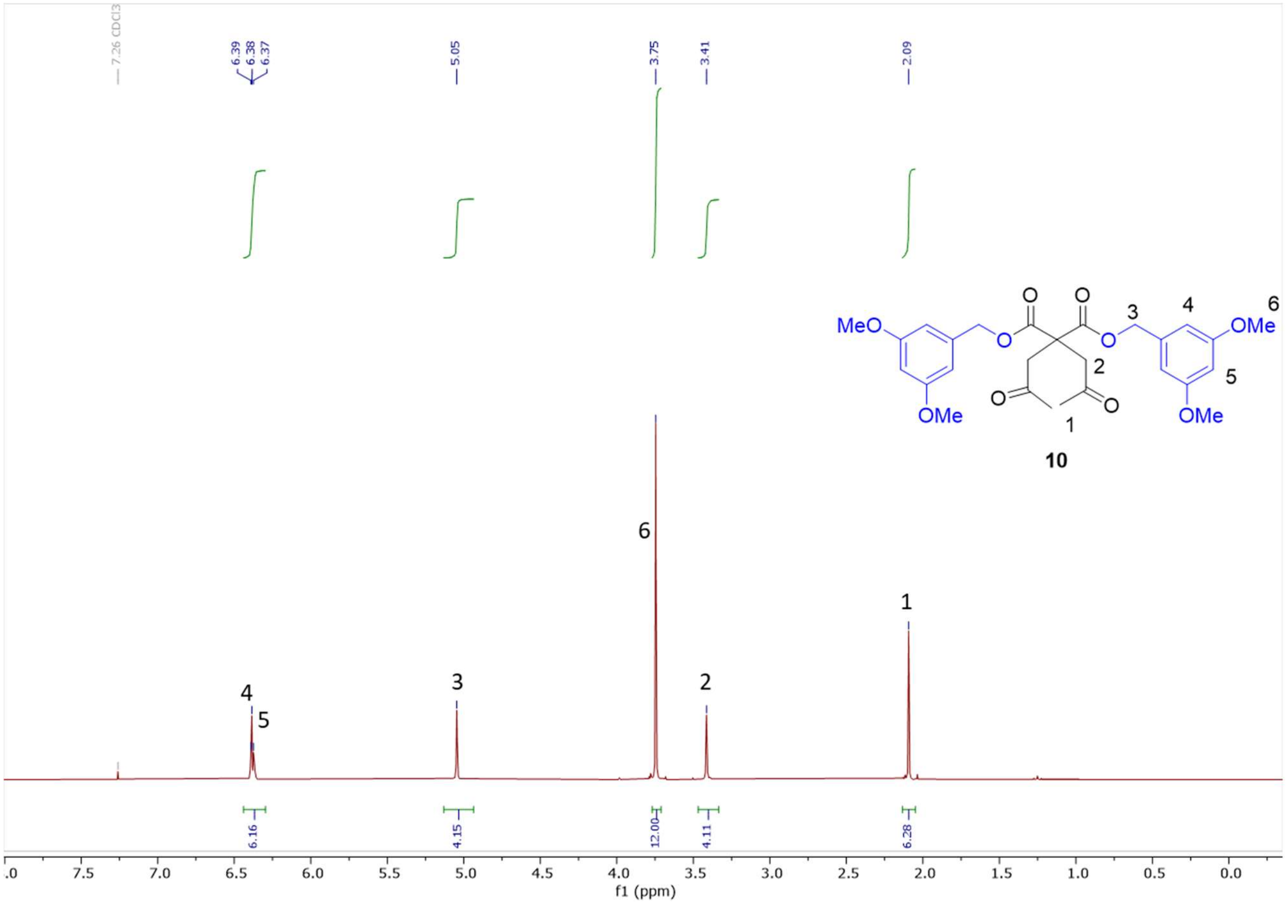

7.26 CDCl3
6.39
6.38
6.37
5.05
3.75
3.41
2.09
MeO
OMe
OMe
OMe
10
6.16
4.15
12.00
4.11
6.28
f1 (ppm)

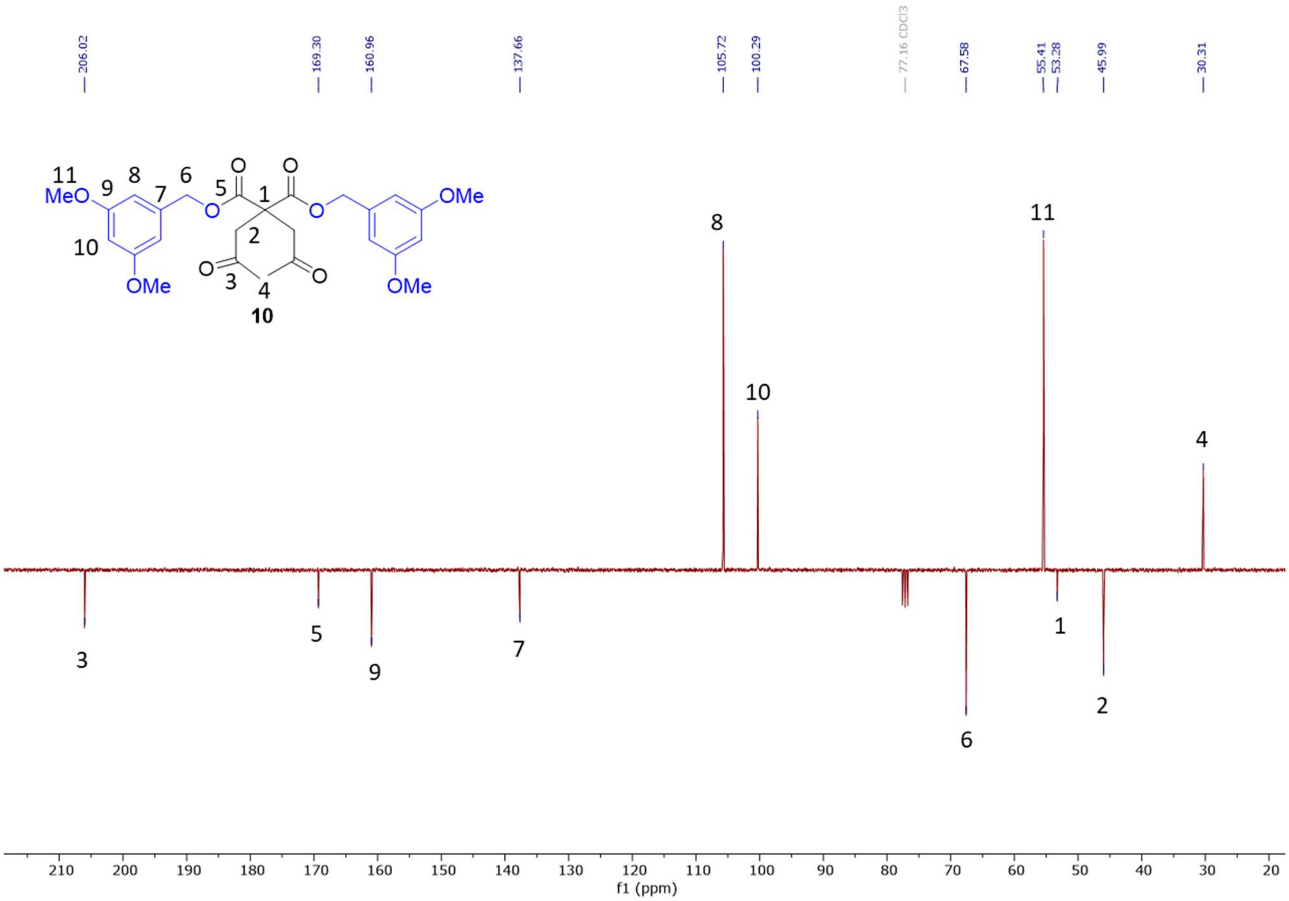

206.02
169.30
160.96
137.66
105.72
100.29
77.16 CDCl3
67.58
55.41
53.28
45.99
30.31
MeO
OMe
OMe
OMe
10
f1 (ppm)

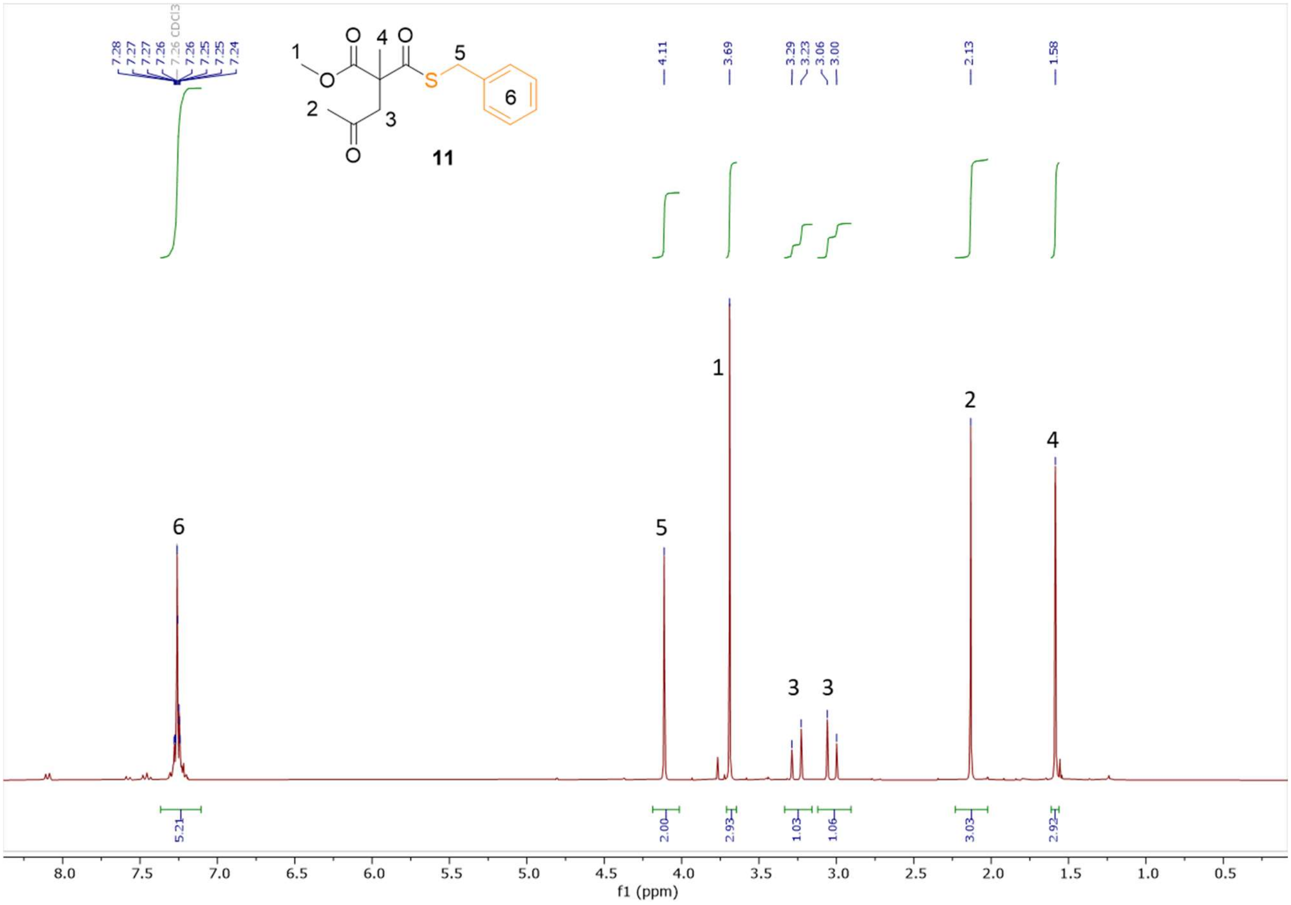

7.28
7.27
7.27
7.26
7.26 CDCl3
7.26
7.26
7.25
7.25
7.24
4.11
3.69
3.29
3.23
3.06
3.00
2.13
1.58
11
6
5
1
3
3
2
4
5.21
2.00
2.93
1.03
1.06
3.03
2.92
8.0
7.5
7.0
6.5
6.0
5.5
5.0
4.5
4.0
3.5
3.0
2.5
2.0
1.5
1.0
0.5
f1 (ppm)

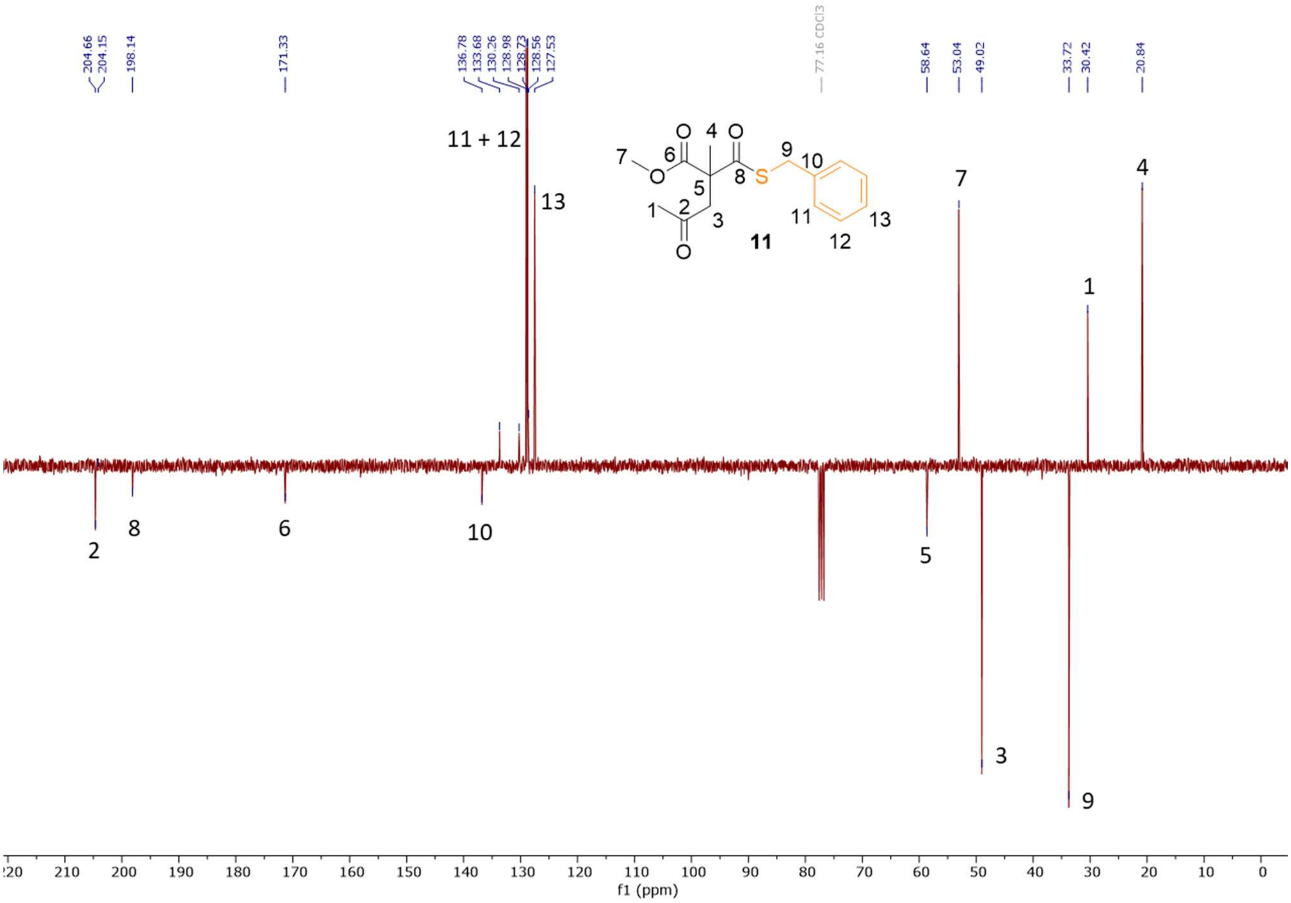

204.66
204.15
198.14
171.33
136.78
133.68
130.26
128.98
128.73
128.56
127.53
77.16 CDCl3
58.64
53.04
49.02
33.72
30.42
20.84
11 + 12
13
11
7
4
1
2
8
6
10
5
3
9
220
210
200
190
180
170
160
150
140
130
120
110
100
90
80
70
60
50
40
30
20
10
0
f1 (ppm)

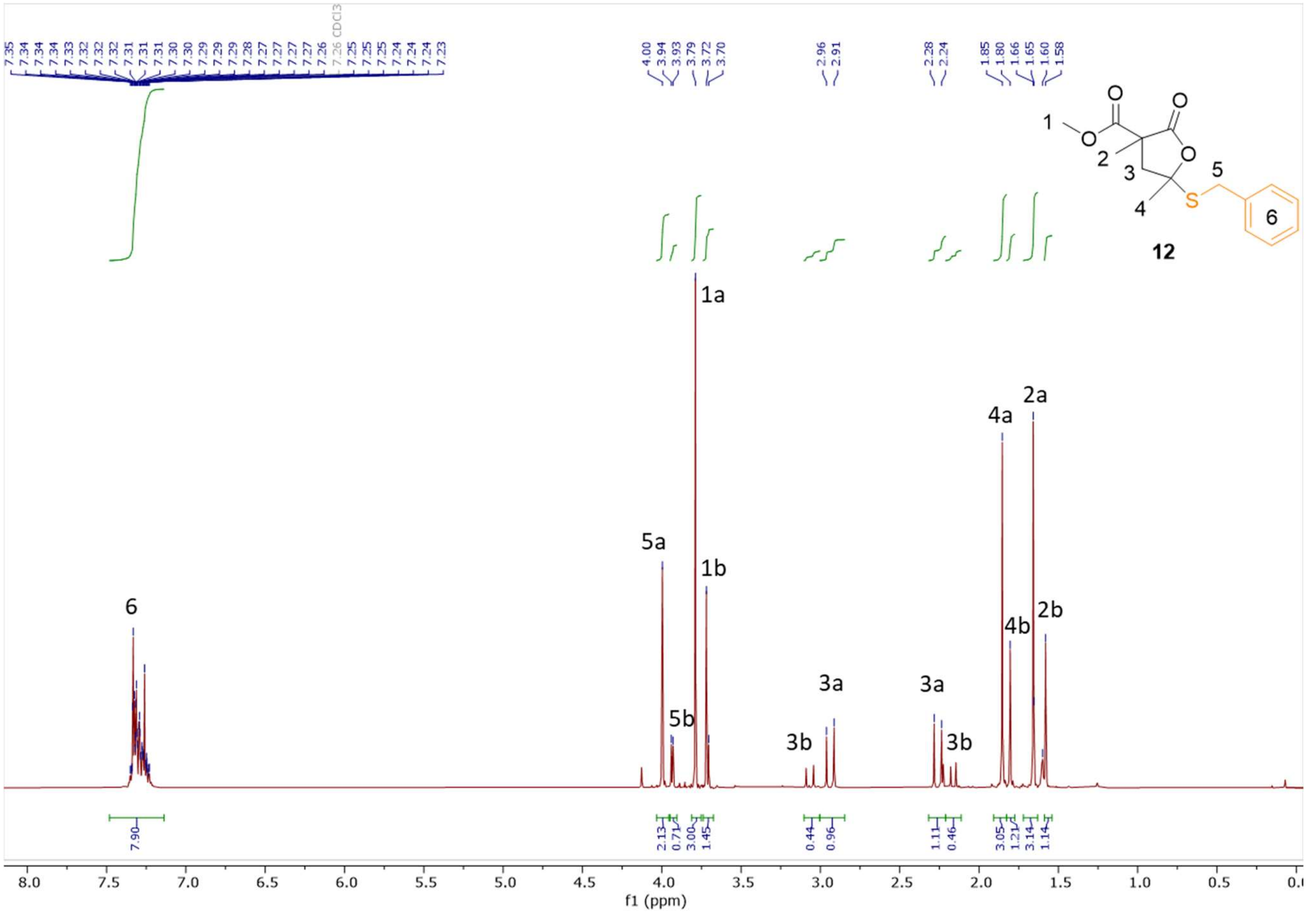

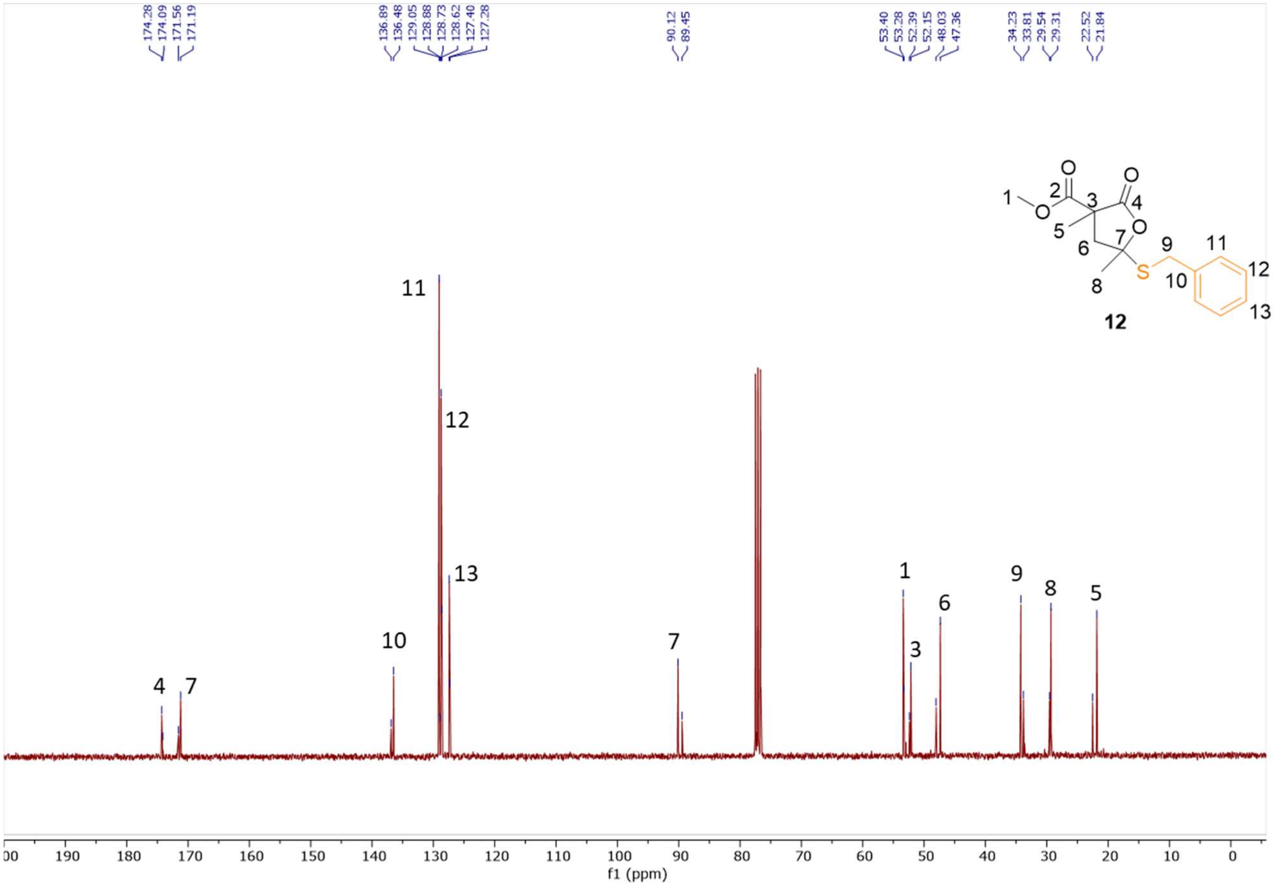

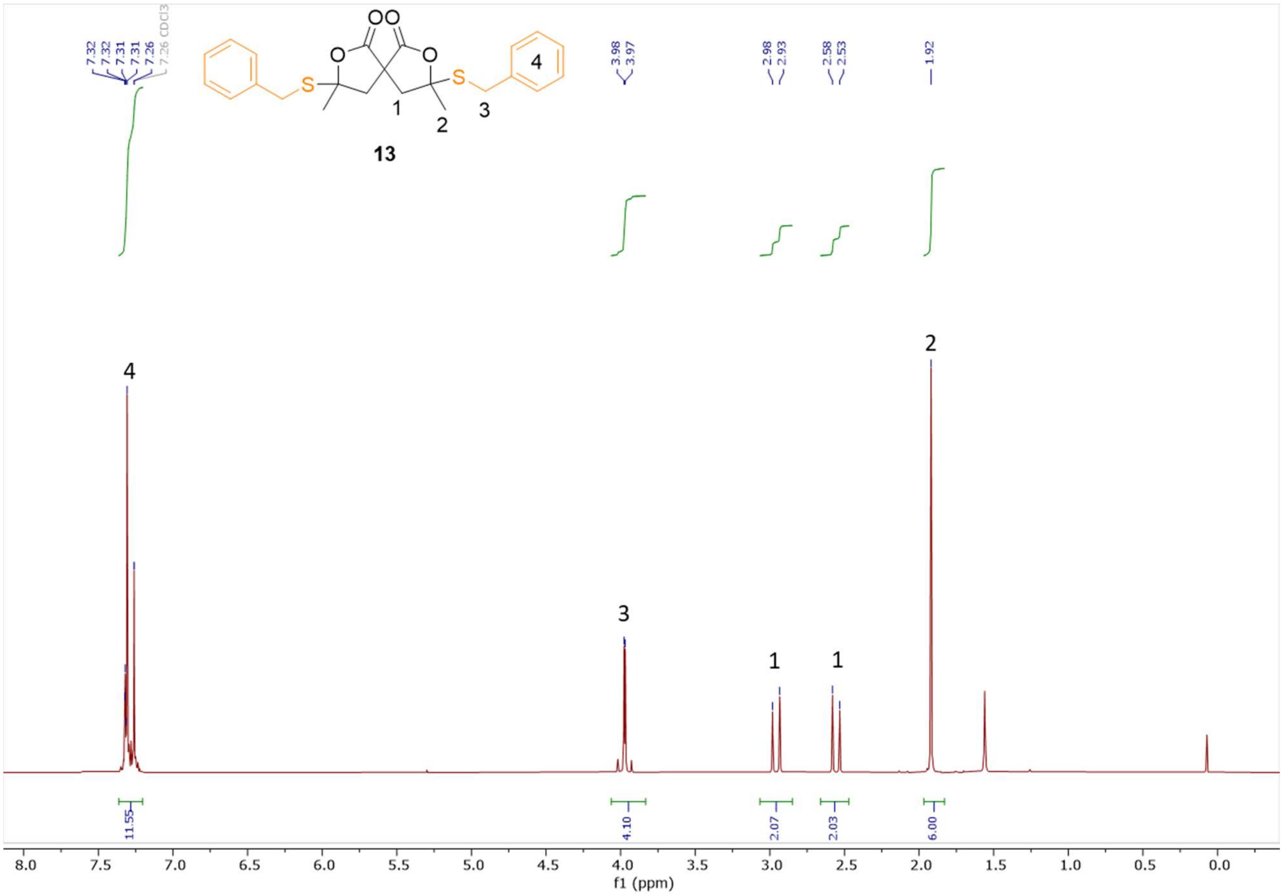
7.32
7.32
7.31
7.31
7.26 CDCl3
3.98
3.97
2.98
2.93
2.58
2.53
1.92
13
4
3
1
2
11.55
4.10
2.07
2.03
6.00
8.0 7.5 7.0 6.5 6.0 5.5 5.0 4.5 4.0 3.5 3.0 2.5 2.0 1.5 1.0 0.5 0.0
f1 (ppm)

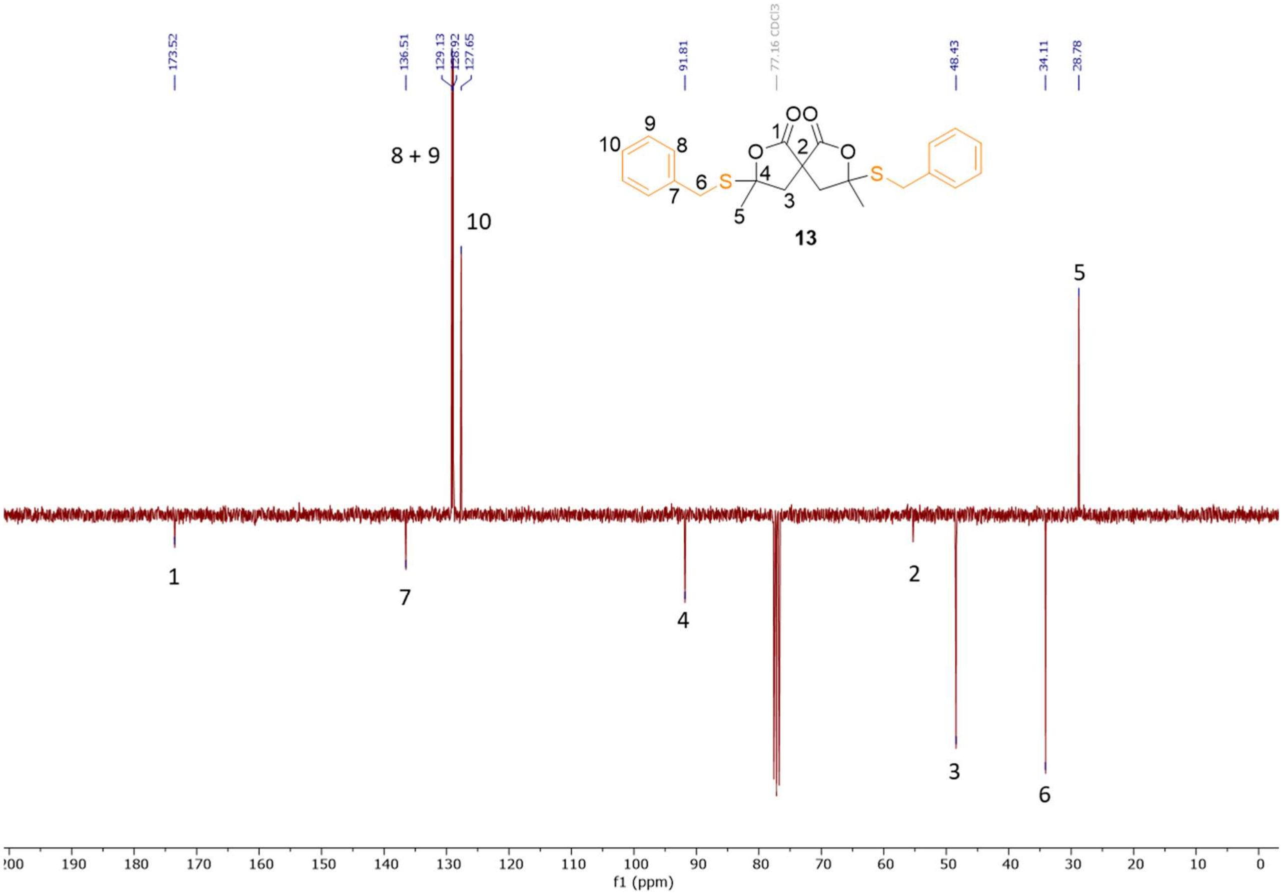
173.52
136.51
129.13
128.92
127.65
91.81
77.16 CDCl3
48.43
34.11
28.78
8 + 9
10
13
5
1
7
4
2
3
6
f1 (ppm)

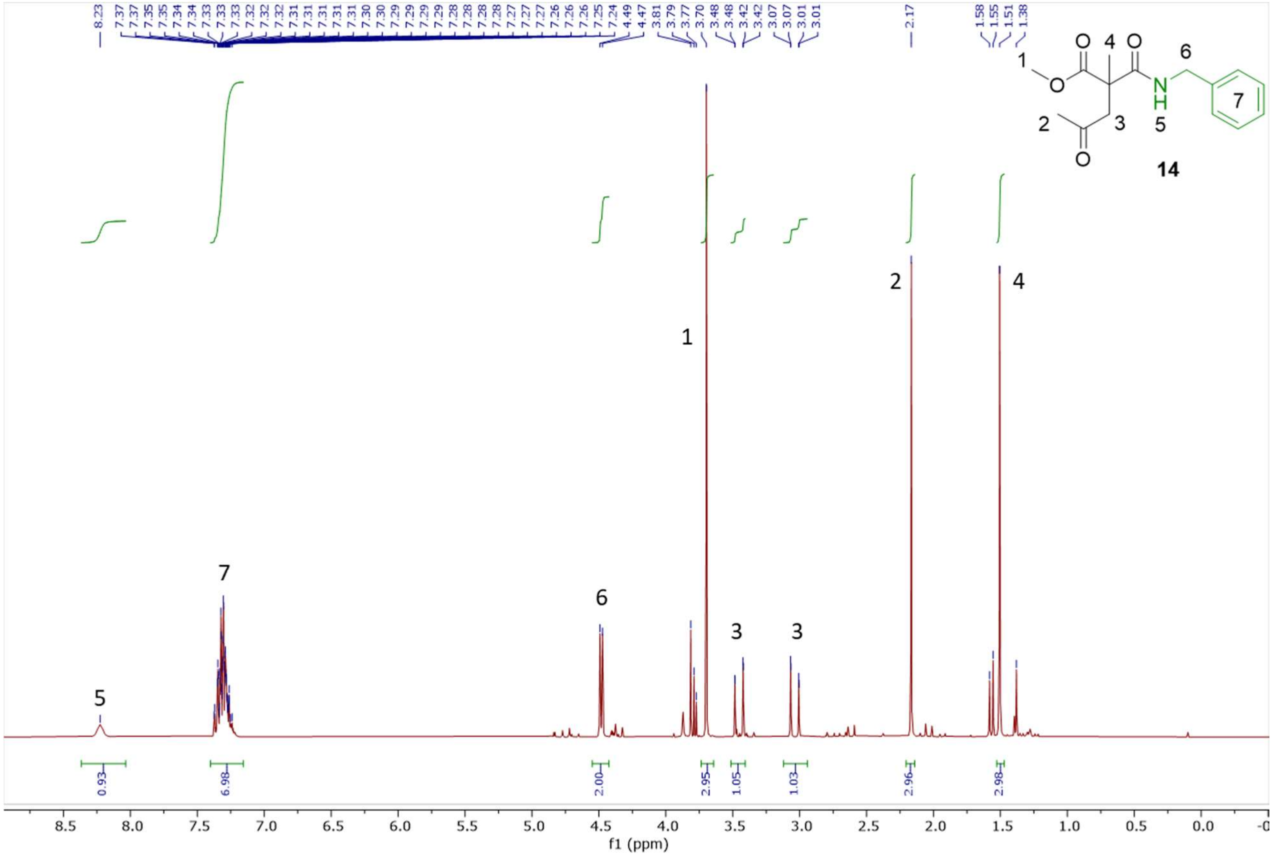
14
1
2
3
4
5
6
7
0.93
6.98
2.00
2.95
1.05
1.03
2.96
2.98
f1 (ppm)

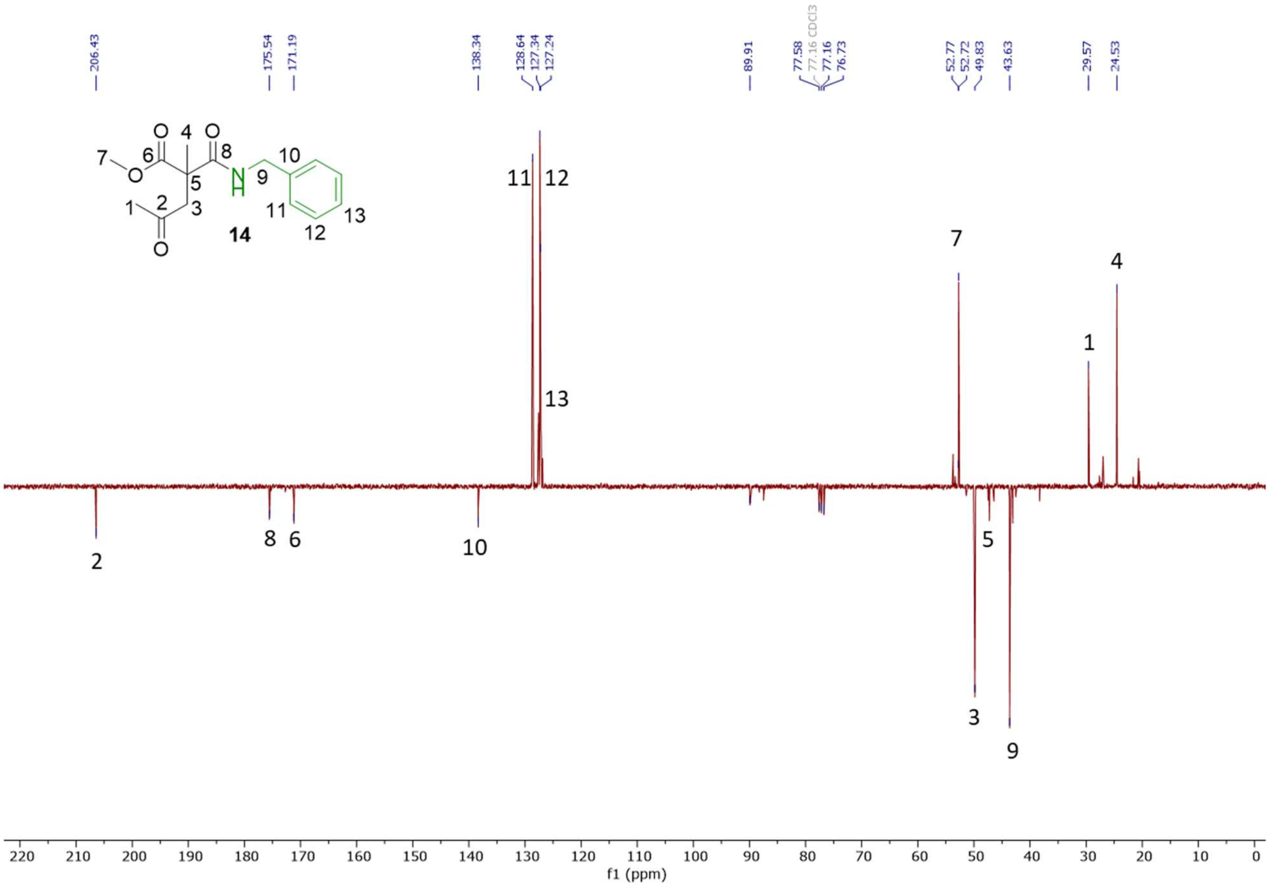
206.43
175.54
171.19
138.34
128.64
127.34
127.24
89.91
77.58
77.16 CDCl3
77.16
76.73
52.77
52.72
49.83
43.63
29.57
24.53
14
f1 (ppm)

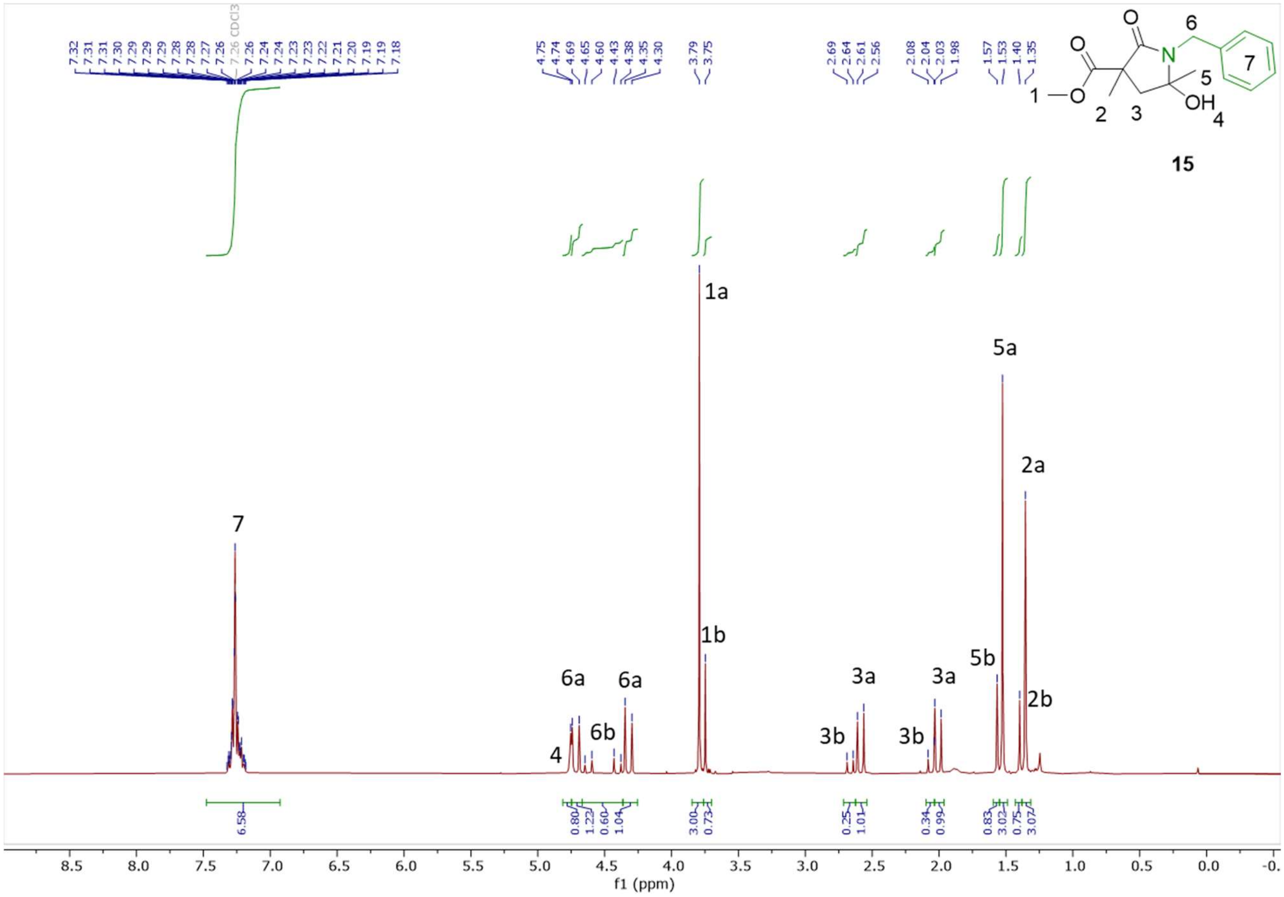
7.26 CDCl3
15
1a
1b
5a
5b
2a
2b
6a
6b
3a
3b
f1 (ppm)

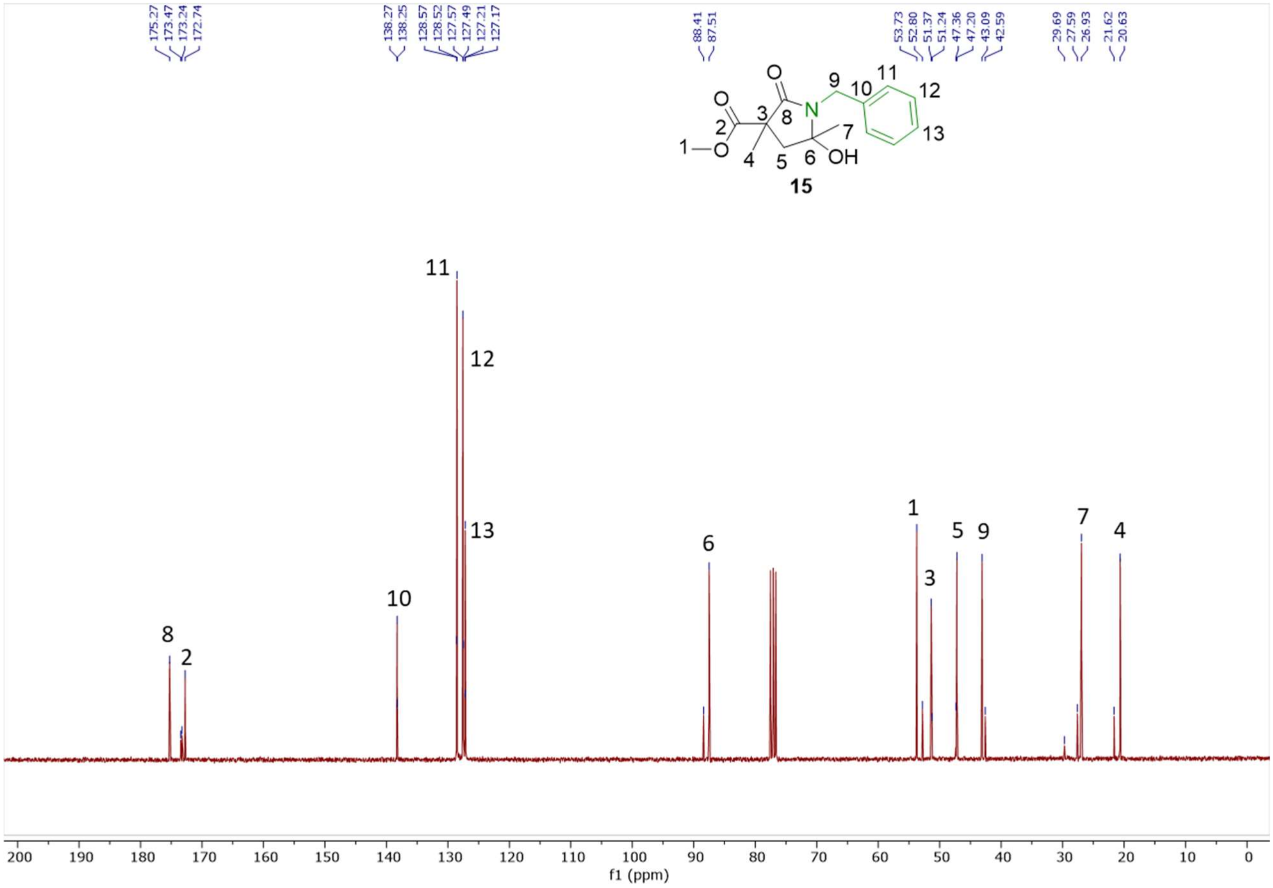
175.27
173.47
173.24
172.74
138.27
138.25
128.57
128.52
127.57
127.49
127.21
127.17
88.41
87.51
53.73
52.80
51.37
51.24
47.36
47.20
43.09
42.59
29.69
27.59
26.93
21.62
20.63
15
f1 (ppm)

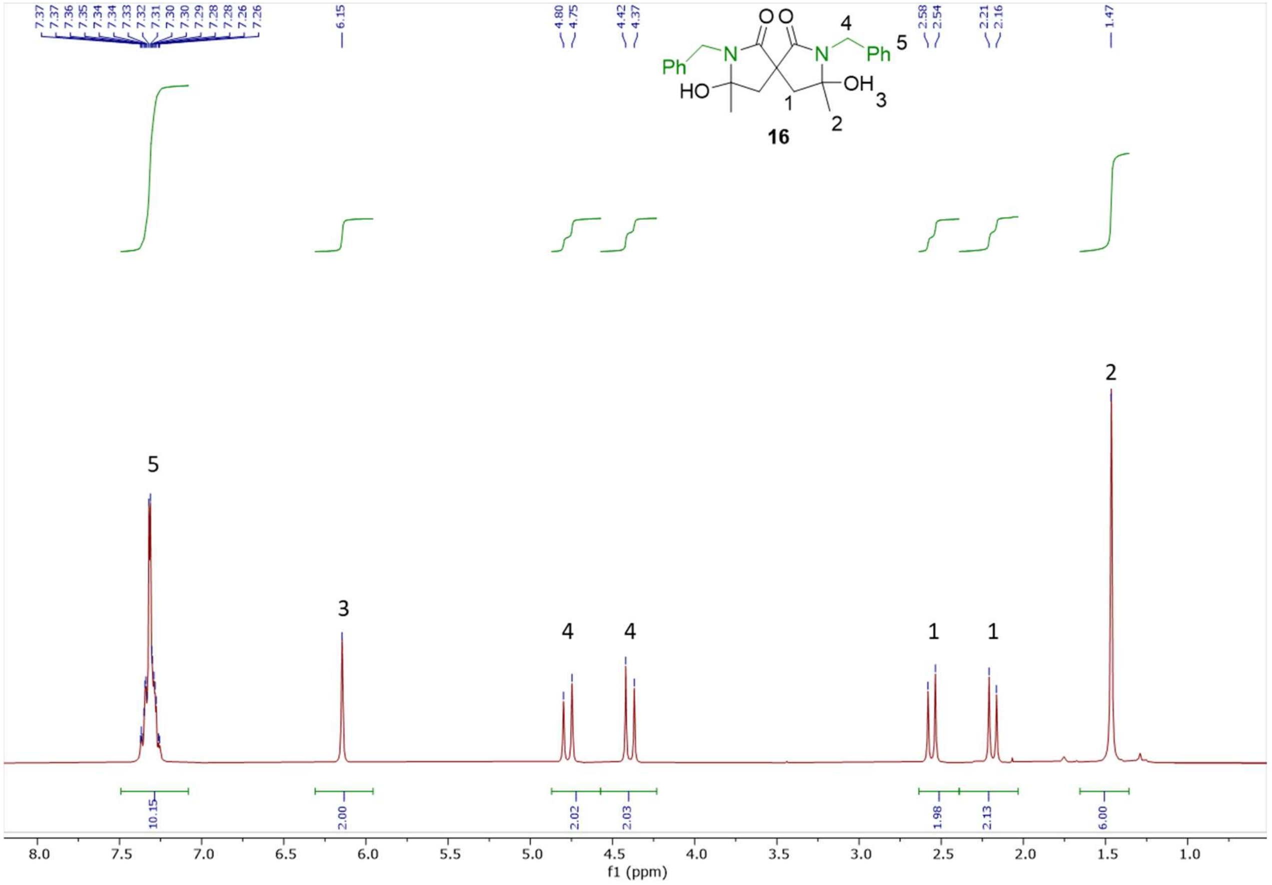
7.37
7.37
7.36
7.35
7.34
7.34
7.33
7.32
7.31
7.30
7.30
7.29
7.28
7.28
7.26
7.26
6.15
4.80
4.75
4.42
4.37
2.58
2.54
2.21
2.16
1.47
Ph
HO
OH
16
10.15
2.00
2.02
2.03
1.98
2.13
6.00
f1 (ppm)

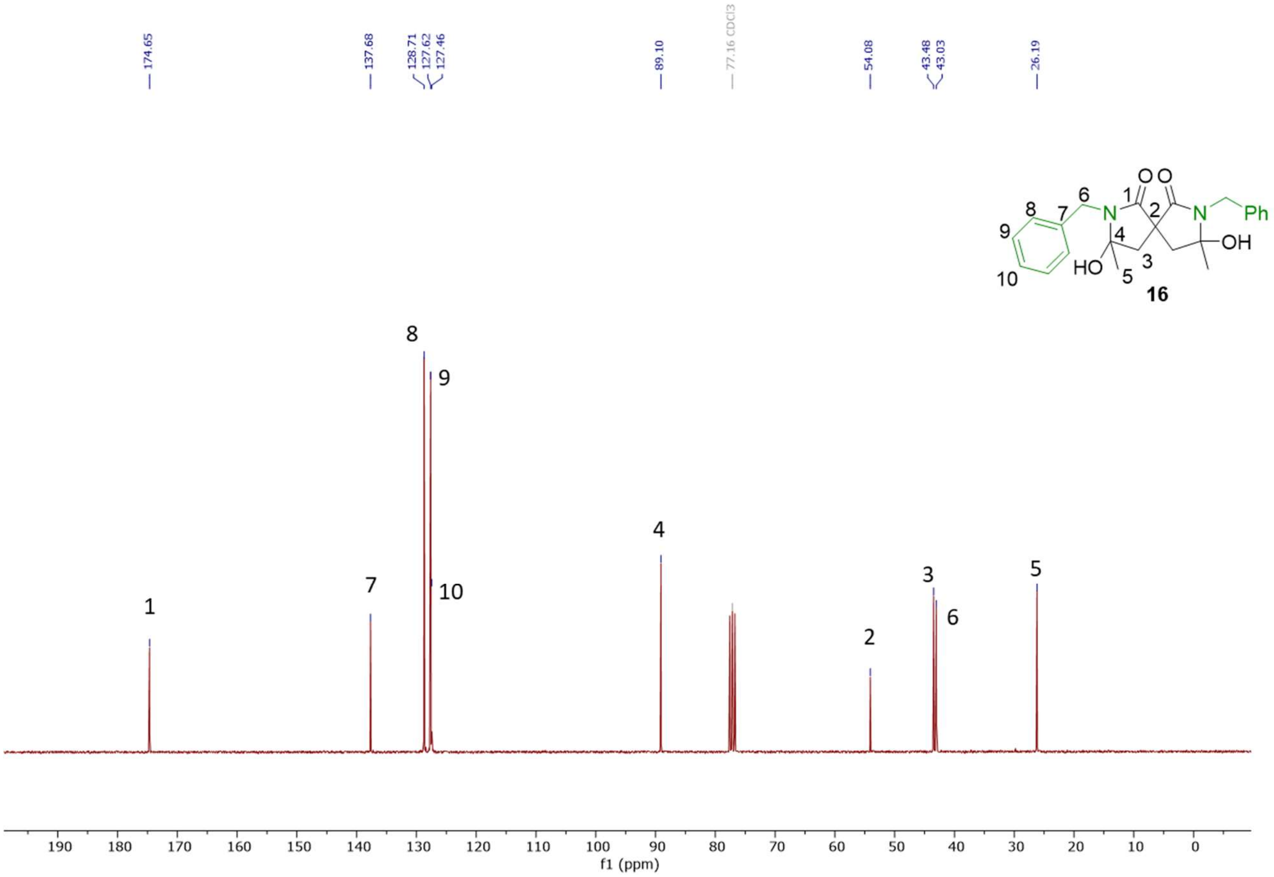
174.65
137.68
128.71
127.62
127.46
89.10
77.16 CDCl3
54.08
43.48
43.03
26.19
16
f1 (ppm)

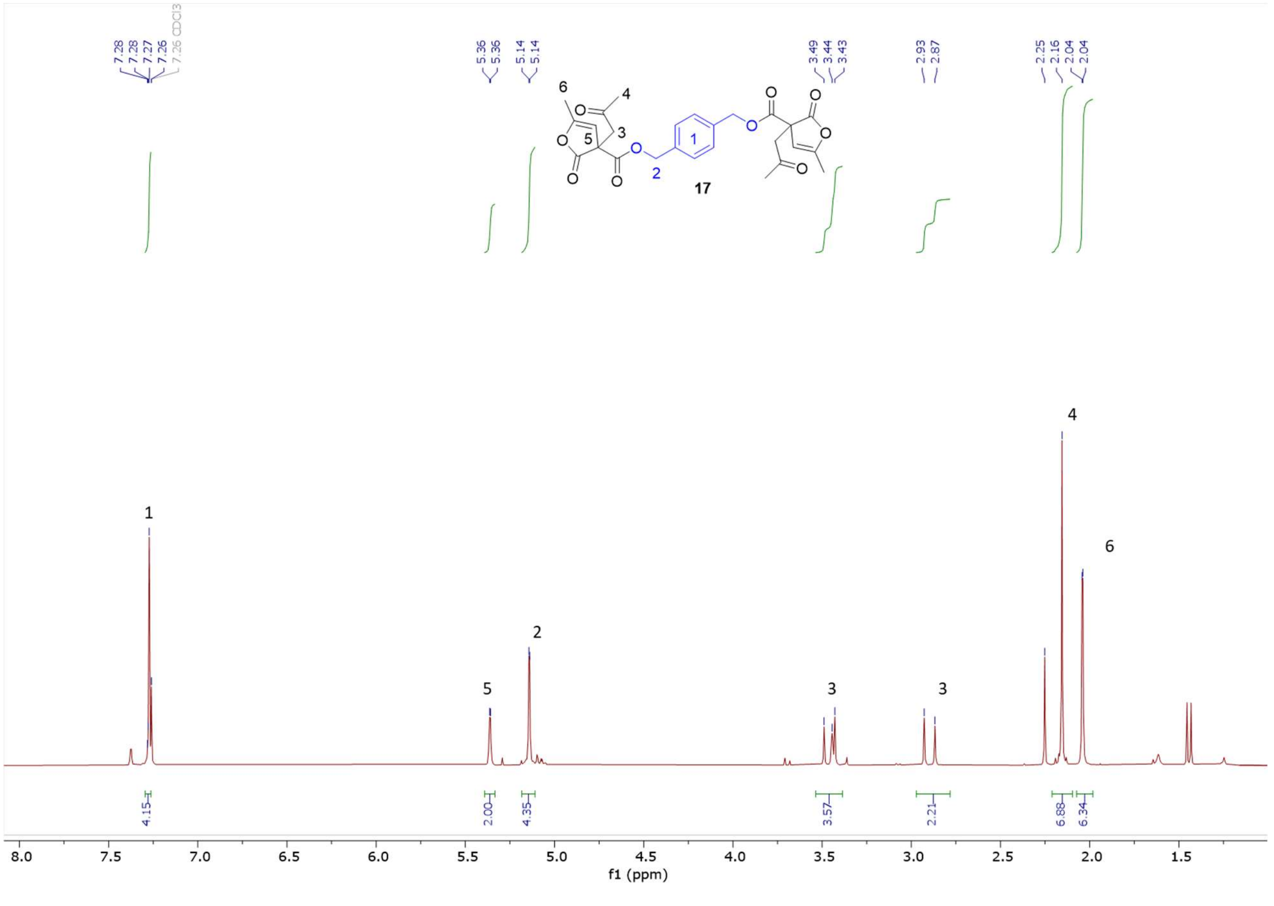
7.28
7.28
7.27
7.26
7.26 CDCl3
5.36
5.36
5.14
5.14
3.49
3.44
3.43
2.93
2.87
2.25
2.16
2.04
2.04
17
4.15
2.00
4.35
3.57
2.21
6.88
6.34
f1 (ppm)

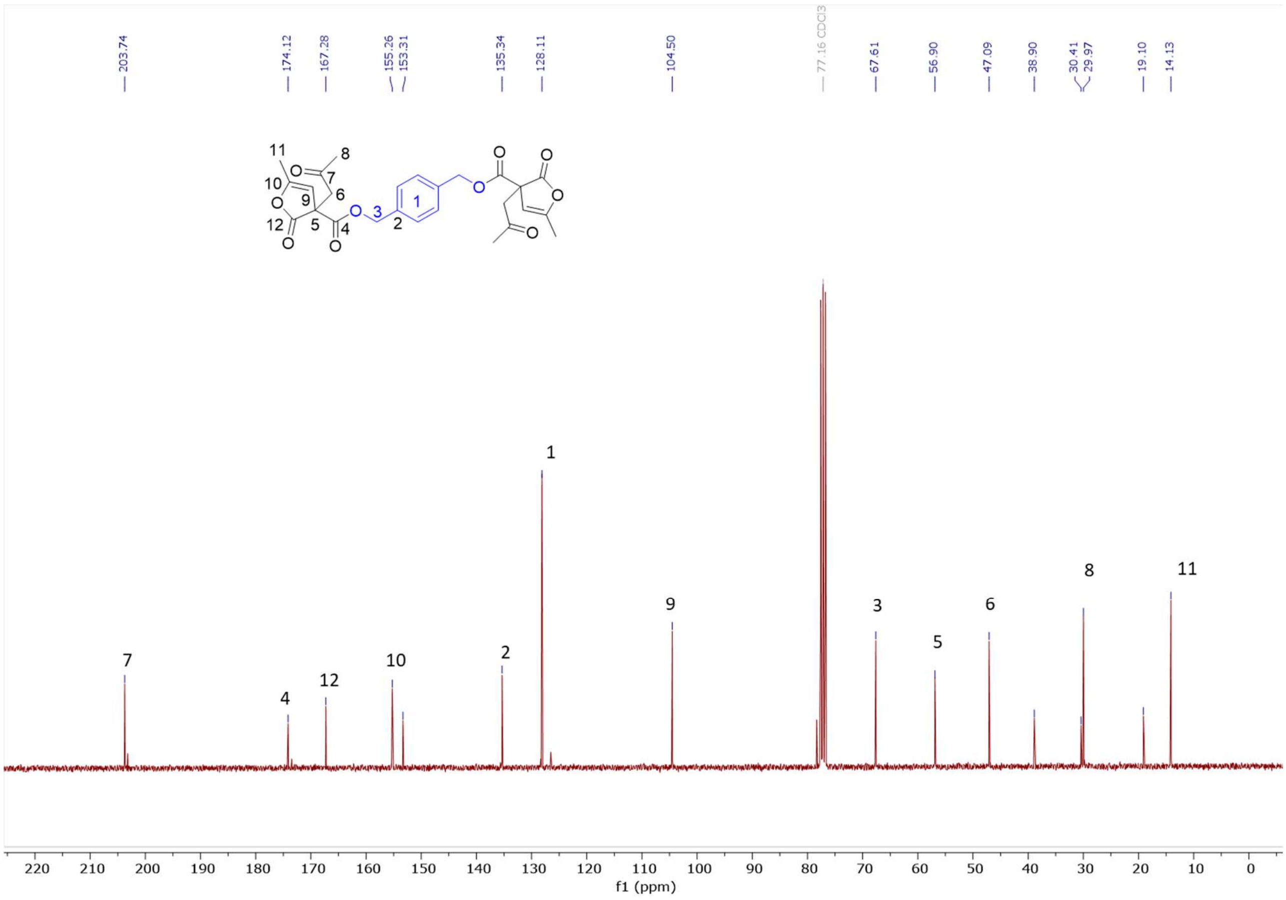
203.74
174.12
167.28
155.26
153.31
135.34
128.11
104.50
77.16 CDCl3
67.61
56.90
47.09
38.90
30.41
29.97
19.10
14.13
f1 (ppm)
